# Turnover and replication analysis by isotope labeling (TRAIL) reveals the influence of tissue context on protein and organelle lifetimes

**DOI:** 10.15252/msb.202211393

**Published:** 2023-03-17

**Authors:** John Hasper, Kevin Welle, Jennifer Hryhorenko, Sina Ghaemmaghami, Abigail Buchwalter

**Affiliations:** ^1^ Cardiovascular Research Institute University of California, San Francisco San Francisco CA USA; ^2^ University of Rochester Mass Spectrometry Resource Laboratory Rochester NY USA; ^3^ Department of Biology University of Rochester Rochester NY USA; ^4^ Department of Physiology University of California, San Francisco San Francisco CA USA; ^5^ Chan Zuckerberg Biohub San Francisco CA USA

**Keywords:** metabolic labeling, protein lifetime, proteostasis, tissue homeostasis, tool development, Proteomics

## Abstract

The lifespans of proteins range from minutes to years within mammalian tissues. Protein lifespan is relevant to organismal aging, as long‐lived proteins accrue damage over time. It is unclear how protein lifetime is shaped by tissue context, where both cell turnover and proteolytic degradation contribute to protein turnover. We develop turnover and replication analysis by ^15^N isotope labeling (TRAIL) to quantify protein and cell lifetimes with high precision and demonstrate that cell turnover, sequence‐encoded features, and environmental factors modulate protein lifespan across tissues*.* Cell and protein turnover flux are comparable in proliferative tissues, while protein turnover outpaces cell turnover in slowly proliferative tissues. Physicochemical features such as hydrophobicity, charge, and disorder influence protein turnover in slowly proliferative tissues, but protein turnover is much less sequence‐selective in highly proliferative tissues. Protein lifetimes vary nonrandomly across tissues after correcting for cell turnover. Multiprotein complexes such as the ribosome have consistent lifetimes across tissues, while mitochondria, peroxisomes, and lipid droplets have variable lifetimes. TRAIL can be used to explore how environment, aging, and disease affect tissue homeostasis.

## Graphical abstract

A new ^15^N metabolic labeling method for parallel quantification of cell and protein turnover rates in mouse tissues illustrates the variable influence of “nature” versus “nurture” on protein turnover rates *in vivo*.

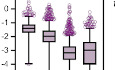

## Introduction

The cellular proteome undergoes constant cycles of synthesis, folding, and degradation. Proteostasis (protein homeostasis) is achieved by the balance of these processes. When these systems function properly, the health of the proteome is ensured by the efficient degradation of misfolded or damaged proteins and replacement with properly folded and functional copies. When proteostasis breaks down due to aging or disease, proteome disruptions including accumulation of oxidative damage, misfolding, and aggregation result (Taylor & Dillin, 2011; Koyuncu *et al*, 2021). Measurements of protein turnover have revealed that protein lifetimes range from minutes to years within mammalian tissues (Price *et al*, 2010; Savas *et al*, 2012; Toyama *et al*, 2013; Fornasiero *et al*, 2018; Mathieson *et al*, 2018). The functional consequences of age‐linked proteostasis collapse are most evident for extremely long‐lived proteins in postmitotic tissues. For instance, crystallin proteins of the eye lens misfold and aggregate over decades, causing cataracts (Taylor & Davies, 1987), while the extremely long‐lived nuclear pore complex becomes leaky and dysfunctional in the aging brain (D'Angelo *et al*, 2009). These striking examples raise several questions, including: what factors control protein lifetime in healthy tissues? What is the relationship between protein longevity and cellular longevity? Why do age‐linked declines in long‐lived protein function manifest only in some tissues?

Protein lifetime can be influenced by both sequence‐encoded features and environmental factors (Marrero & Barrio‐Hernandez, 2021). For instance, proteins with long disordered segments are generally more short‐lived than proteins that adopt a stable structure (van der Lee *et al*, 2014; Fishbain *et al*, 2015). Posttranslational modifications have varied effects on protein stability (Wu *et al*, 2021; Zecha *et al*, 2022), while higher buried surface area correlates with longer lifetime (Mallik & Kundu, 2018). There are many exceptions that break these rules, however, and it is unclear to what extent physicochemical features predict protein lifetime *in vivo*. Additionally, the cellular, tissue, and organismal environments of proteins can strongly influence their degradation rates. For instance, the same protein sequence can have dramatically different lifetimes when expressed in different cell types, tissues, or organisms (Toyama *et al*, 2013; Dörrbaum *et al*, 2018; Matsuda *et al*, 2020; Swovick *et al*, 2020; Rolfs *et al*, 2021).

**Figure 1:**
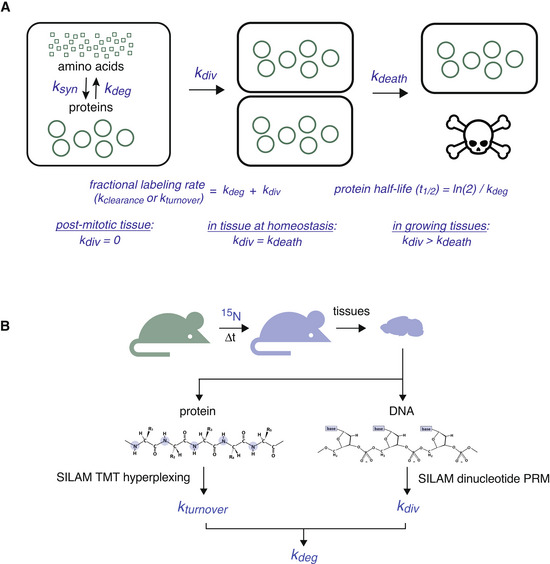
Schematic of turnover and replication analysis by isotope labeling (TRAIL) approach The fractional rate by which a protein population is turned over within a cell (*k*_t_) can be determined by measuring the fractional rate of isotope incorporation in continuous labeling experiments. This rate is established by the additive effects of protein degradation (*k*_deg_) and cell division (*k*_div_). In postmitotic cells, *k*_div_ is negligible and does not contribute to protein turnover. In proliferating tissues, the rate of cell division is balanced by the rate of cell death (*k*_death_).Diagram of TRAIL approach for simultaneously quantifying *k*_t_ and *k*_div_. These two rates are measured by quantifying rates of ^15^N incorporation into proteins and DNA within the same measurement. Together, these two measurements can be used to accurately measure *k*_deg_.

One important environmental parameter that can strongly influence the observed turnover rate of a protein is the proliferative capacity of the tissue where it is expressed. Protein clearance (on a per cell basis) is influenced by the additive effects of its degradation kinetics as well as cellular dilution due to cell division (Price *et al*, 2010; Toyama *et al*, 2013; Fig 1A). Thus, in general, protein clearance rates are expected to be faster within proliferative tissues in comparison to nonproliferative tissues. However, in typical dynamic metabolic labeling experiments employed for measurements of *in vivo* protein turnover, potential differences in tissue proliferation rates are unknown, making it impossible to deconvolute the effects of protein degradation and dilution. Thus, with currently available methodology, it is not possible to account for differences in tissue proliferation when comparing protein turnover rates across tissues.

To accurately measure *in vivo* protein turnover rates within multiple tissues, we sought to develop a mass spectrometry‐based method capable of simultaneously quantifying *in vivo* protein degradation and cell division rates within a single labeling experiment. While metabolic labeling with ^13^C or ^15^N isotopes has become the gold standard for the quantification of protein turnover rates (McClatchy *et al*, 2007; Price *et al*, 2010), this methodology has not been integrated with cell turnover measurements. Instead, cell turnover rates are frequently measured by partial labeling with nucleotide analogs (e.g., ^3^H‐thymidine or BrdU), an approach that is often limited by label toxicity (Reome *et al*, 2000; Asher *et al*, 2009). Alternatively, D_2_O labeling has been used to quantify cell turnover (Neese *et al*, 2002) or to measure bulk rates of protein turnover and nucleic acid turnover (Drake *et al*, 2013; Thompson *et al*, 2016). However, only low levels of D_2_O can be tolerated *in vivo*, and the small mass shifts that are achieved by partial labeling require specialized analysis methods for quantitation (Miller *et al*, 2020). Here, we describe methods to measure both protein degradation and cell division within mammalian tissues using a single source of label: the stable isotope ^15^N (Fig 1B). We name this suite of methods “turnover and replication analysis by isotope labeling”, or turnover and replication analysis by isotope labeling (TRAIL). We apply TRAIL to proliferative and nonproliferative tissues and generate a rich dataset that reveals tissue‐specific features of proteostasis. We find evidence for sequence‐based selectivity in protein turnover in tissues that undergo slow cell proliferation, while protein turnover is much less selective in highly proliferative tissues. Furthermore, protein and organelle lifetimes vary widely across healthy tissues even after correcting for cell proliferation rates. These observations illustrate the variable influence of “nature” (sequence‐encoded features) versus “nurture” (environmental factors) on proteostasis *in vivo*. In the future, TRAIL can be used to explore how environment, aging, and disease affect tissue homeostasis.

## Results

### Increasing throughput of stable isotope labeling time courses by tandem mass tag multiplexing

Proteome‐wide quantification of protein stability can be achieved *in vivo* by feeding mice a food source containing ~ 100% abundance of the stable, nontoxic isotope ^15^N, a method referred to as stable isotope labeling in mammals (SILAM; McClatchy *et al*, 2007; Price *et al*, 2010). Labeled tissues are then analyzed by tandem mass spectrometry (LC–MS/MS) to quantify the incorporation of labeled amino acids into the proteome over time (McClatchy *et al*, 2007; Price *et al*, 2010). Broader application of SILAM has been limited by the investment of resources and time required to complete these types of analyses. One major bottleneck is mass spectrometer run time, which rapidly multiplies when each sample must be analyzed in a separate LC–MS/MS run. Furthermore, protein “dropout” due to missing values limits the number of proteins whose turnover kinetics can be precisely determined. We have previously used tandem mass tagging (TMT) to “hyperplex” pools of isotope‐labeled samples in a single LC–MS/MS run, which decreases cost while increasing speed and sensitivity (Welle *et al*, 2016). Here, we adapt this approach to ^15^N‐labeled samples from mouse tissue (TMT‐SILAM, Appendix Fig S1). Hyperplexed analysis of ^15^N‐ labeled samples presents a unique challenge due to the high complexity of the labeled peptide spectra. The gradual labeling of the *in vivo* amino acid precursor pool by ^15^N results in broadened MS1 spectra whose average mass to charge ratios increase as a function of labeling time (Price *et al*, 2010), creating a challenging analysis problem that requires specialized data analysis workflows (Guan *et al*, 2011, 2012). However, we previously demonstrated that hyperplexed sample analysis can be simplified by quantifying the relative decay of unlabeled peaks as a function of time rather than the fractional population of unlabeled and labeled peaks (Welle *et al*, 2016). Here, by quantifying the fractional rate of loss of ^14^N peptides (as newly synthesized ^15^N‐labeled peptides accumulate), we were able to directly measure the turnover rate of pre‐existing unlabeled proteins (Appendix Fig S1).

We performed a 32‐day TMT‐SILAM time course on young adult (9‐week‐old) mice. Animal weights remained stable through the labeling period (Appendix Fig S2), indicating that protein levels are at a steady‐state and fractional labeling rates can be equated with protein turnover rates (Ross *et al*, 2021; see Materials and Methods). We focused our analyses on selected tissues that are thought to be either highly proliferative or largely postmitotic (Sender & Milo, 2021): the large intestine (a proliferative tissue); the liver (a quiescent tissue that can proliferate in response to injury); and the heart and white adipose tissue, which are mostly postmitotic. We analyzed the labeling kinetics of thousands of proteins per tissue (Appendix Fig S3) and filtered these data at several levels to compile high‐quality datasets. Experimental replicates were first filtered based on coverage: only proteins that were detected with a minimum of three peptide spectral matches (PSMs) in all channels were retained for further analysis. Second, aggregated replicate data were used to determine the rate constant for protein turnover (*k*_t_) by least squares fitting to a first‐order kinetic model (Note that *k*_t_ values refer to protein turnover rate constants that have not been corrected for the dilution effects of cell division as described below). Only *k*_t_ values that were measured by fitting data arising from at least two replicates with a high goodness of fit (*t*‐statistic > 3; Appendix Fig S4; see Materials and Methods) were considered in downstream analyses.

### Features of protein turnover across tissues

**Figure 2:**
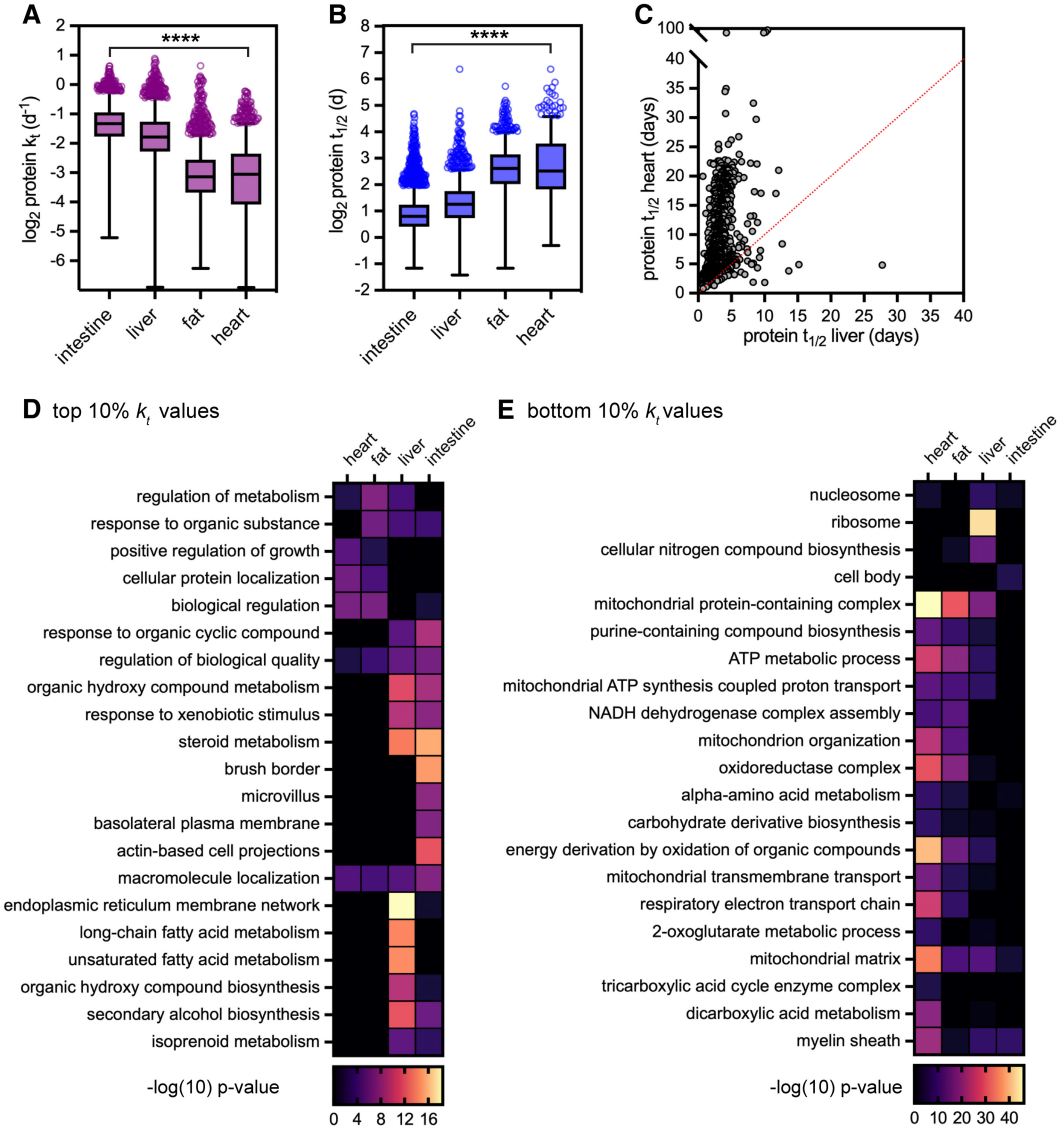
Proteome turnover measurements for four reference tissues A, BTurnover rates (*k*_t_) (A) and predicted half‐lives (*t*_1/2_) (B) determined by TMT‐SILAM of proteins extracted from intestine (*n* = 2,719; median *t*_1/2_ 1.7 days), liver (*n* = 2099, median *t*_1/2_ 2.4 days), fat (*n* = 1,610, median *t*_1/2_ 6.1 days), and heart (*n* = 1,635, median *t*_1/2_ 5.7 days). Box (Tukey) plot center line indicates median; box limits indicate 25^th^ to 75^th^ percentiles; whiskers indicate 1.5× interquartile range; points indicate outlier values. ****indicates that all medians are significantly different (*P* < 0.0001, Kruskal–Wallis test). See also Dataset EV1 for full dataset.CPredicted half‐lives (*t*_1/2_) for 1,102 intracellular proteins in the heart versus the liver.D, EHeatmaps of GO term enrichment in the top 10% (least stable, D) and the bottom 10% (most stable, E) of *k*_t_ values for intracellular proteins detected in each tissue.

Altogether, we defined high‐confidence *k*_t_ values (Fig 2A) and corresponding predicted half‐life (*t*_1/2_) values (Fig 2B) for thousands of proteins per tissue: 2,719 in the large intestine, 2,099 in liver, 1,610 in white adipose tissue, and 1,635 in the heart (Appendix Fig S3; Dataset EV1). Protein abundance and protein lifetime were generally not correlated with each other (Appendix Fig S5). The distributions of *k*_t_ values were unique for each tissue; proteins were more short‐lived in the intestine (median *t*_1/2_ 1.7 days) and liver (median *t*_1/2_ 2.4 days), but more long‐lived in the fat (median *t*_1/2_ 6.1 days) and heart (median *t*_1/2_ 5.7 days). Differences in protein stability have been previously reported between mammalian tissues, such as the brain, liver, and muscle (Price *et al*, 2010; Toyama *et al*, 2013; Rolfs *et al*, 2021). We compared our datasets to previous analyses of proteome turnover in the liver (Price *et al*, 2010) and heart (Lau *et al*, 2016) and found high concordance in both cases (Appendix Fig S6).

We explored and excluded several potential explanations for tissue‐specific differences in protein stability. (i) These differences persist among the subset of proteins whose lifetimes were quantified across all four tissues (902 proteins total; Appendix Fig S7A and B; Dataset EV2), indicating that differences in protein lifetime are not due simply to differences in proteome composition. Furthermore, a large proportion of protein lifetime differences are statistically significant in pairwise comparisons between tissues, while 282 proteins (31%) have significantly different *k*_t_ values across all four tissues (Appendix Fig S7C; Dataset EV2). (ii) These differences also persist if secreted proteins are excluded from analyses (Appendix Fig S7D–F); secreted proteins are a unique class of proteins whose turnover is difficult to accurately profile *in vivo*, as their sites of synthesis, function, and degradation can be quite disparate. Even after controlling for these factors, differences in protein lifetimes across tissues are readily apparent. For example, many intracellular proteins have days‐long lifetimes in the liver but weeks‐long lifetimes in the heart (Fig 2C), with 85% of these proteins having significantly different *k*_t_ values between these two tissues (Appendix Fig S7C).

We looked more closely at intracellular proteins at the extremes of stability by identifying gene ontology (GO) terms that were overrepresented in either the top decile (Fig 2D) or bottom decile (Fig 2E) of *k*_t_ values in each tissue. The identity of the most short‐lived and most long‐lived proteins varies widely. For instance, proteins involved in intestinal nutrient absorption are enriched among the most short‐lived proteins of the large intestine, while proteins involved in alcohol and fatty acid metabolism are found among the most short‐lived proteins of the liver (Fig 2D). At the other extreme, components of chromatin are enriched in the most long‐lived proteins of the liver and intestine, while proteins involved in various mitochondrial functions are enriched in the most long‐lived proteins of the heart and adipose tissue (Fig 2E). Interestingly, while components of chromatin and mitochondria have been found to be long‐lived in other protein turnover studies (Price *et al*, 2010; Toyama *et al*, 2013; Bomba‐Warczak *et al*, 2021; Krishna *et al*, 2021), our data suggest that their relative stability varies from tissue to tissue.

**Figure 3:**
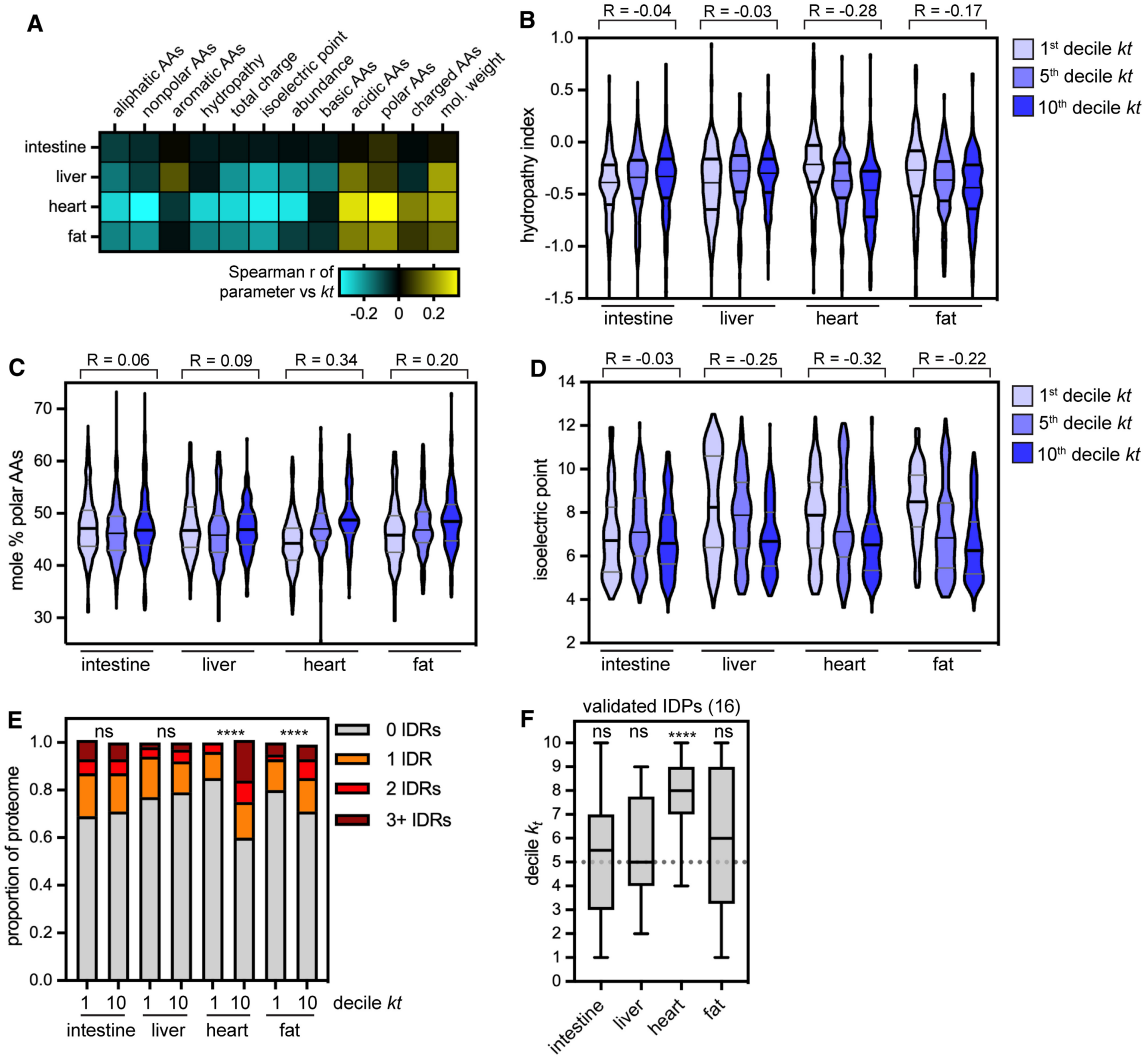
Analysis of correlations between protein sequence features and protein turnover rate across tissues Heatmap of Spearman correlation coefficient between *k*_t_ and protein sequence features (see Materials and Methods).GRAVY hydropathy index of proteins in the 1^st^, 5^th^, and 10^th^ *k*_t_ deciles in each tissue; hydropathy is significantly anticorrelated with *k*_t_ in heart and fat proteomes, but not in intestine or liver proteomes.Isoelectric point of proteins in the 1^st^, 5^th^, and 10^th^ *k*_t_ deciles in each tissue; pI is significantly anticorrelated with *k*_t_ in the liver, heart, and fat proteomes, but not in the intestine proteome.Abundance of polar amino acids (mole% of D, E, H, K, N, Q, R, S, T) in the 1^st^, 5^th^, and 10^th^ *k*_t_ deciles in each tissue; polar/charged residue abundance is significantly positively correlated with *k*_t_ in the heart and fat proteomes, but not in the intestine or liver proteomes.Incidence of proteins containing long (> 40 AA) intrinsically disordered regions (IDRs) in the 1^st^ and 10^th^ *k*_t_ deciles in each tissue. IDR‐containing proteins are significantly overrepresented in the most short‐lived proteins in the heart and fat proteomes, but not in the intestine or liver proteomes. ****indicates *P* < 0.0001; significance determined by χ ^2^ test for between 161 and 272 proteins per decile (see Source Data for Fig 3).Turnover rates (*k*_t_ decile) of 16 experimentally IDPs are significantly faster than the proteome median in the heart, but not in other tissues. IDP annotations from the DisProt database. See also Source Data for Fig 3 and Dataset EV3 for full table of protein sequence features. Box (Tukey) plot center line indicates median; box limits indicate 25^th^ to 75^th^ percentiles; whiskers indicate 1.5× interquartile range; points indicate outlier values. ****indicates *P* < 0.0001 by one‐sample *t*‐test. Source data are available online for this figure.

We next explored how physicochemical properties such as amino acid composition, hydrophobicity, charge and intrinsic disorder correlate with protein turnover (Fig 3; Dataset EV3). While these relationships have been explored within protein turnover datasets acquired in yeast (Martin‐Perez & Villén, 2017) and in cultured cells (van der Lee *et al*, 2014; Fishbain *et al*, 2015; Marrero *et al*, 2017), they have not to our knowledge been evaluated across mammalian tissues. Overall, we did not find a single protein feature that correlated significantly with protein turnover rate across all tissues. Rather, we found features that showed significant relationships to protein turnover in a subset of tissues (Fig 3A). For instance, hydrophobicity decreases as turnover rate increases in the heart and fat proteomes, but not in the liver or intestine proteomes (Fig 3A and B). In these same tissues, polar amino acids are more abundant in short‐lived proteins than in long‐lived proteins (Fig 3A and C). Protein isoelectric point is strongly anticorrelated with *k*_t_, such that long‐lived proteins are more basic (pI > 7) while short‐lived proteins are more acidic (pI < 7) in the heart, liver, and fat (Fig 3A and D). Consistently, acidic amino acids are overrepresented in short‐lived proteins in these tissues (Fig 3A). Finally, we evaluated relationships between protein disorder and protein turnover by quantifying the frequency of intrinsically disordered regions (IDRs) in the most stable and least stable proteins (Fig 3E). IDRs of at least 40 amino acids in length are correlated with significantly accelerated protein turnover across eukaryotes (van der Lee *et al*, 2014; Fishbain *et al*, 2015). While IDRs are overrepresented in unstable heart and fat proteins, there is no relationship between disorder and protein lifetime in the intestine or liver (Fig 3E). In an orthogonal approach, we evaluated the turnover rates of an experimentally validated list of disordered proteins from the DisProt database (Quaglia *et al*, 2021) and found that this validated group of disordered proteins turned over significantly faster than the proteome median within the heart, but not in any other tissue (Fig 3F; Appendix Fig S7G and H). Instead, many of these disordered proteins are rapidly degraded in one tissue but relatively stable in another. Taken together, our analyses reveal greater sequence‐based selectivity of turnover of the heart and fat proteome than of the liver and intestine proteome.

We speculate that the interplay between sequence features and environmental factors influences protein lifetime *in vivo*. Within a living tissue, the rates of protein turnover are influenced both by proteolytic degradation of proteins and by dilution of proteins during cell turnover (Fig 1A). Variations in the extent of cell turnover could, at least in part, underlie the observed differences in protein turnover rates across tissues (Fig 2). Differences in cell turnover may also influence the observed differences in distributions of protein turnover rates within a given tissue (Fig 3) as cell turnover nonselectively accelerates apparent protein turnover rates among all proteins within a given tissue. To accurately define the relationship between protein lifetime and cellular lifetime, a method that can quantify both of these parameters in parallel is needed.

### TRAIL to profile cell and protein turnover

We sought to develop a method to quantify cell turnover rates in parallel with protein turnover measurements. It is well appreciated that ^15^N‐labeled nutrients supplied via SILAM chow can efficiently label proteins in mice. However, diet‐supplied ^15^N can also be robustly incorporated into genomic DNA via nitrogen‐containing nucleobases (Drigo *et al*, 2019; Appendix Fig S8). We, therefore, reasoned that tracking the rate of ^15^N incorporation into the genome via DNA replication would yield cell division rates (*k*_div_), which when conducted in conjunction with analyses of protein labeling could be used to determine corrected protein degradation rates (*k*_deg_; Ross *et al*, 2021; Fig 1B, Appendix Fig S9). We refer to this method as TRAIL.

To develop this approach, we first needed to address the technical barrier imposed by the prevalence of *in vivo* nucleotide recycling. The fractional labeling of genomic DNA during an isotope labeling experiment is influenced both by the rate of replication and by the relative isotope abundance (RIA) of the precursor nucleotide pool (Appendix Fig S9). The latter is strongly influenced by precursor uptake from the diet, nucleotide biosynthesis, and nucleotide recycling *in vivo* (Neese *et al*, 2002). Incomplete labeling of the precursor pool due to low precursor uptake, slow *de novo* biosynthesis, or extensive recycling of pre‐existing nucleic acids would decrease the extent of ^15^N incorporation into replicating genomic DNA^32^. Furthermore, the relative contributions of each of these factors may vary across tissues. Thus, it is important to define the RIA of the precursor nucleotide pool in each tissue in order to accurately determine the rate of replication by measuring the fractional labeling of genomic DNA. We reasoned that we could deconvolute the RIA of the precursor nucleotide pool by analyzing the combinatorics of labeling in contiguous stretches of *dinucleotides* obtained from the same strand of genomic DNA (Appendix Figs S9 and S10; see Materials and Methods). A strand of DNA that has been synthesized in the presence of label will contain labeled nucleotides at a frequency that is contingent on the RIA of the precursor pool. Analysis of the isotopologue distribution within dinucleotides enables the calculation of the prevalence of recycled unlabeled nucleotides within the precursor pool. Thus, we can determine what fraction of the observed fully unlabeled dinucleotide population was derived from pre‐existing unlabeled DNA strands, and what fraction was derived from newly synthesized strands that incorporated unlabeled recycled nucleotides. Through this deconvolution, we can measure the relative ratio of old and newly synthesized DNA and determine the rate of cell proliferation. We digested genomic DNA from SILAM‐labeled mouse tissue to short oligonucleotides using the enzyme benzonase (Liao *et al*, 2007), then quantified ^15^N/^14^N isotope abundance ratios in dinucleotides by mass spectrometry. We found that the ^15^N RIA of the precursor pool was very high in all tissues, and that diet‐derived ^15^N‐labeled nucleic acids were preferentially incorporated into newly synthesized genomic DNA (Appendix Fig S10C). This is in line with the fact that nucleotide salvage pathways are repressed in S phase while *de novo* nucleotide synthesis is upregulated, so that cells primarily rely on the latter source of nucleotides for DNA replication (Reichard, 1988). This outcome is also consistent with observations from other modes of DNA labeling (Macallan *et al*, 1998).

Our finding that diet‐derived and *de novo* synthesized nucleic acids are preferred for DNA replication implies that we can make an accurate measurement of DNA replication rates by tracking ^15^N incorporation into either mononucleosides or dinucleotides isolated from genomic DNA. We tested this by isolating free dA, dC, dT, and dG mononucleosides from genomic DNA by digestion with a cocktail of benzonase, phosphodiesterase, and alkaline phosphatase (Quinlivan & Gregory, 2008) and quantifying ^15^N/^14^N isotope ratios by mass spectrometry (see Materials and Methods). Decay curves for all four mononucleosides were in close alignment both with each other (Appendix Fig S11A) and with dinucleotide curves (Appendix Fig S10D), indicating that TRAIL is highly precise and reproducible.

To test how accurately TRAIL reports cell division rates, we devised a benchmarking experiment as follows. We isolated fibroblasts from the ear of a mouse that had undergone ^15^N labeling for a total of 256 days. Because mouse fibroblasts renew within weeks, the genomic DNA from these cells was highly labeled with ^15^N. We then subcultured these fibroblasts *ex vivo* and collected genomic DNA at three timepoints over the course of several days. In parallel, we quantified cell numbers. We then compared the doubling times determined by TRAIL versus direct measurement of population doublings. These data were highly consistent (Appendix Fig S12), indicating that TRAIL accurately tracks cell division rate.

**Figure 4:**
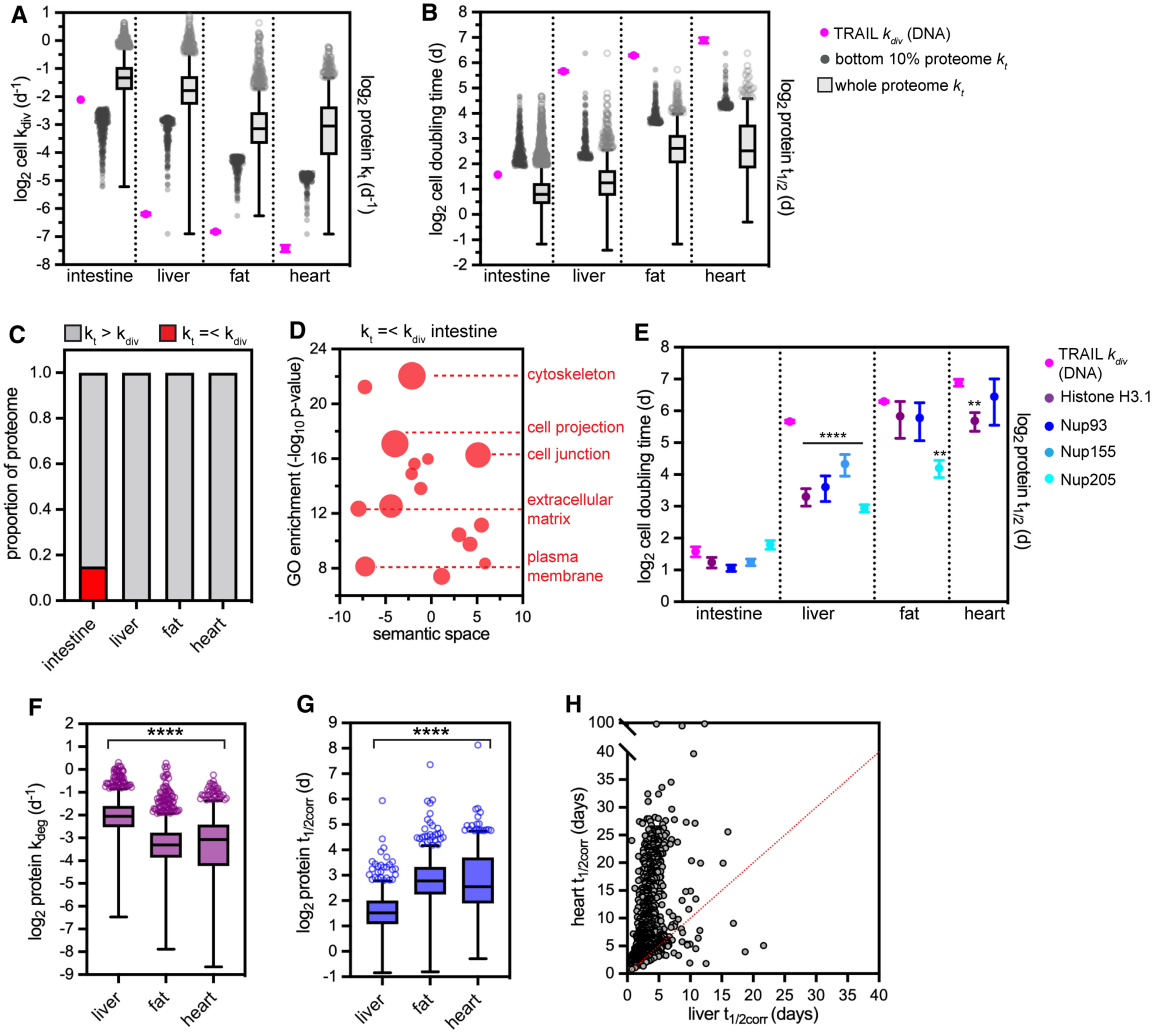
Analysis of relationships between cell turnover rate and protein turnover rate across tissues A, B(A) Comparison of cell division rates (*k*_div_) to protein turnover rates (*k*_t_) and (B) of cell doubling time to protein lifetime (*t*_1/2_) in intestine, liver, fat, and heart, determined by TRAIL. Cell turnover rates were determined from 6‐timepoint time courses with three tissue samples per timepoint. Proteome *k*_t_ and *t*_1/2_ values reproduced from Fig 3A and B. Bottom 10% of proteome corresponds to 271 proteins (intestine); 209 proteins (liver); 161 proteins (fat); and 163 proteins (heart). Box (Tukey) plot center line indicates median; box limits indicate 25^th^ to 75^th^ percentiles; whiskers indicate 1.5× interquartile range; points indicate outlier values. See also Dataset EV4.COnly the proliferative intestine has a significant number of proteins whose *k*_t_ is equal to or less than *k*_div_, suggesting that these proteins are diluted by cell division.DA subset of Gene Ontology (GO) terms (cellular component) overrepresented in proteins that are diluted out by cell division in the intestine are shown in a bubble plot. Redundant GO terms were removed and nonredundant GO terms were organized based on semantic similarity by REViGO. Bubble size corresponds to number of proteins associated with GO term, ranging in size from 20 to 120.EComparison of cell doubling time determined by TRAIL to the lifetime of the replication‐dependent histone H3.1 and to nucleoporins Nup93, Nup155, and Nup205. Error bars indicate SEM. ****indicates *P* < 0.0001 and **indicates *P* < 0.01, *t*‐test.F, GCell cycle‐corrected degradation rates (*k*_deg_) (F) and half‐lives (*t*_1/2corr_) (G) for 967 proteins detected in liver (median *t*_1/2corr_ 2.9 days), fat (median *t*_1/2corr_ 6.8 days), and heart (median *t*_1/2corr_ 5.9 days). Box (Tukey) plot center line indicates median; box limits indicate 25^th^ to 75^th^ percentiles; whiskers indicate 1.5× interquartile range; points indicate outlier values. ****indicates that all medians are significantly different (*P* < 0.0001, Kruskal–Wallis test).HPredicted cell cycle‐corrected half‐lives (*t*_1/2corr_) for 1,102 intracellular proteins in the heart versus the liver. See also Dataset EV5 for full dataset.

With these important controls established, we then applied TRAIL to the large intestine. We determined a doubling time of ~ 3 days for this proliferative tissue, in close agreement with previous analyses by orthogonal methods (Darwich *et al*, 2014; Sender & Milo, 2021; Fig 4A and B). We then applied TRAIL to the liver, fat, and heart. Each of these tissues have long average doubling times indicating low proliferative capacity (Fig 4A and B; Dataset EV4). These data are qualitatively consistent with previous reports of low proliferation in these tissues (MacDonald, 1961; Rigamonti *et al*, 2011; Malliaras *et al*, 2013). Importantly, bulk tissue measurements report a weighted average of the turnover rates of a tissue's major constituent cell types, such that both the relative abundance and the relative turnover rate of each cell type contribute to the overall turnover observed. We referred to single cell sequencing (scSeq)‐based tissue atlases to estimate the major cell types in each tissue analyzed, which are as follows (Appendix Fig S13). Intestine: epithelia, followed by enterocytes and goblet cells (Neff *et al*, 2018); liver: hepatocytes, followed by hepatobiliary cells and lymphocytes (Richter *et al*, 2021); fat: adipocyte precursors, mature adipocytes, and macrophages (Emont *et al*, 2022); and heart: cardiomyocytes, cardiac fibroblasts, and endothelial cells (Hu *et al*, 2018). Many of these cell types express unique sets of proteins. To understand whether cell type‐specific proteins are present in our datasets, we referred to cell type‐resolved proteome datasets, where available (Azimifar *et al*, 2014), or to cell type markers determined by scSeq (Hu *et al*, 2018). Hepatocytes are the majority cell type in the liver; we detected only 11 hepatocyte‐specific proteins (Azimifar *et al*, 2014) in our dataset of 2,099 proteins, and detected no proteins unique to rarer cell types such as Kupffer cells (Appendix Fig S13; Datasets EV1 and EV5). In the heart, cardiomyocytes and cardiac fibroblasts are the two most abundant cell types; relying on unique markers of these cell types identified by scSeq (Hu *et al*, 2018), we could detect only 35 cardiomyocyte‐specific proteins and eight cardiac fibroblast‐specific proteins in our dataset of 1,635 proteins (Appendix Fig S13; Datasets EV1 and EV5). We were not able to identify a high‐confidence list of proteins or transcripts specific to abundant intestinal or adipose tissue cell types. Altogether, these analyses indicate that while a small number of cell type‐specific proteins may be detectable from the most abundant cell types in a tissue, the vast majority of the proteins detected tend to be abundant and broadly expressed across cell types. It is thus reasonable to evaluate how tissue‐averaged cell turnover relates to tissue‐averaged protein turnover of these broadly expressed proteins.

### Protein degradation rates vary across tissues after cell cycle correction

A powerful feature of TRAIL is the ability to cocapture cell and protein turnover from the same tissues. Comparing these two metrics revealed that cell turnover and protein turnover flux are comparable to each other in the intestine (cell doubling time of 3 days vs. median protein lifetime of 1.7 days; Fig 4A and B). In striking contrast, cell turnover occurs orders of magnitude more slowly than protein turnover in the liver (cell doubling time 51 days; median protein lifetime 2.4 days), fat (cell doubling time 78 days; median protein lifetime 6.1 days), and heart (cell doubling time 118 days; median protein lifetime 5.7 days; Fig 4A and B). These observations indicate that dilution by cell division contributes significantly to protein turnover in highly proliferative tissues but not in slowly proliferative tissues. In the intestine, cellular *k*_div_ was roughly equivalent to or faster than protein *k*_t_ for approximately 15% of the proteome (Fig 4C; Appendix Fig S11B); these long‐lived proteins are components of cell surface and extracellular structures including the extracellular matrix and cell–cell junctions (Fig 4D). In the slowly proliferative liver, fat, and heart, in contrast, most long‐lived proteins turn over at a slow rate that exceeds the rate of cell division (Fig 4A–C). We were able to evaluate the turnover of two types of known long‐lived proteins: replication‐dependent histones and nuclear pore complex (NPC) components. The turnover of Histone H3.1 has been used as a proxy for cell division in protein turnover studies (Toyama *et al*, 2013; Dörrbaum *et al*, 2018) because Histone H3.1 is incorporated into nucleosomes as a heterodimer with Histone H4 solely after DNA replication (Wu *et al*, 1982), leading to the expectation that this protein's levels would decrease by dilution over successive cell divisions. If this assumption is correct, the lifetime of Histone H3.1 should be very similar to the DNA replication rate reported by TRAIL. This is the case in the intestine and fat, but the lifetime of Histone H3.1 is significantly shorter than the average cell doubling time in the slowly proliferative liver and heart (Fig 4E). We speculate that this difference reflects DNA replication‐independent processes that regulate the lifetime of H3.1. For instance, H3.1/H4 dimers can be evicted from DNA during transcription and are replaced with heterodimers of Histone H3.3 and Histone H4 (Ahmad & Henikoff, 2002). Separately, histones can also be found in cytosolic pools in complex with chaperones, where they may be more rapidly turned over (Cook *et al*, 2011). We speculate that each of these factors contributes to the turnover of this histone isoform in postmitotic tissues over long timescales. The NPC gates transport between the nucleus and cytoplasm; based on its crucial role in nuclear function and on the long lifetime of the core structural scaffold of the NPC in the brain (Toyama *et al*, 2013, 2019), it has been proposed that the NPC does not turn over for the lifetime of the cell. However, NPC components are not as long‐lived in the liver (Toyama *et al*, 2013) or in some cultured cell types (Mathieson *et al*, 2018). Differences in cell division rates were proposed to underlie the variability in lifetime of NPC components, a hypothesis that we can directly test with TRAIL. Our data indicate that components of the Nup93 subcomplex (Nup93, Nup155, and Nup205) turn over at rates similar to cell turnover in the intestine, fat, and heart, but turn over significantly more rapidly than the rate of cell turnover in the liver (Fig 4E). TRAIL thus reveals contextual variability in the rate of turnover of long‐lived proteins across tissues.

To determine to what extent cell turnover contributes to apparent protein turnover rate, *k*_div_ values can be subtracted from protein *k*_t_ values to extrapolate corrected protein degradation rates (*k*_deg_; Ross *et al*, 2021; Fig 1A). We examined *k*_deg_ values in the liver, heart, and fat, where protein *k*_t_ rates far outpace cell *k*_div_ rates. If variable cell turnover rates underlie the variability in protein *k*_t_ values across tissues, *k*_deg_ values should be largely invariant after correcting for *k*_div_. We did not observe this outcome. Instead, the range of *k*_deg_ values remained distinct from tissue to tissue even after correcting for cell turnover rates, and persisted when we restricted our analysis only to broadly expressed proteins that were detected in all tissues (967 proteins; Fig 4E and F; Datasets EV5 and EV6). Overall, the liver proteome (median *t*_1/2corr_ 2.9 days) turns over significantly more rapidly than the fat proteome (median *t*_1/2corr_ 6.8 days; 90% of *k*_deg_ values are significantly different) or heart proteome (median *t*_1/2corr_ 5.8 days; 84% of *k*_deg_ values are significantly different) after cell cycle correction (Fig 4G and H). These data indicate that protein lifetime is broadly influenced by other environmental factors beyond cell proliferation rate. Consistent with our findings, protein lifetimes have also been found to differ significantly between nondividing cell types in culture (Dörrbaum *et al*, 2018), as well as in the same cell type (fibroblasts) isolated from different mammals (Swovick *et al*, 2020). One potential explanation for these differences could be variation in the composition and activity of protein folding chaperones, the ubiquitin‐proteasome system, and/or the autophagy machinery across tissues (Mizushima *et al*, 2004; Jenkins *et al*, 2020; Vonk *et al*, 2020).

### Peroxisomes, lipid droplets, and mitochondria have highly variable lifetimes across tissues

**Figure 5:**
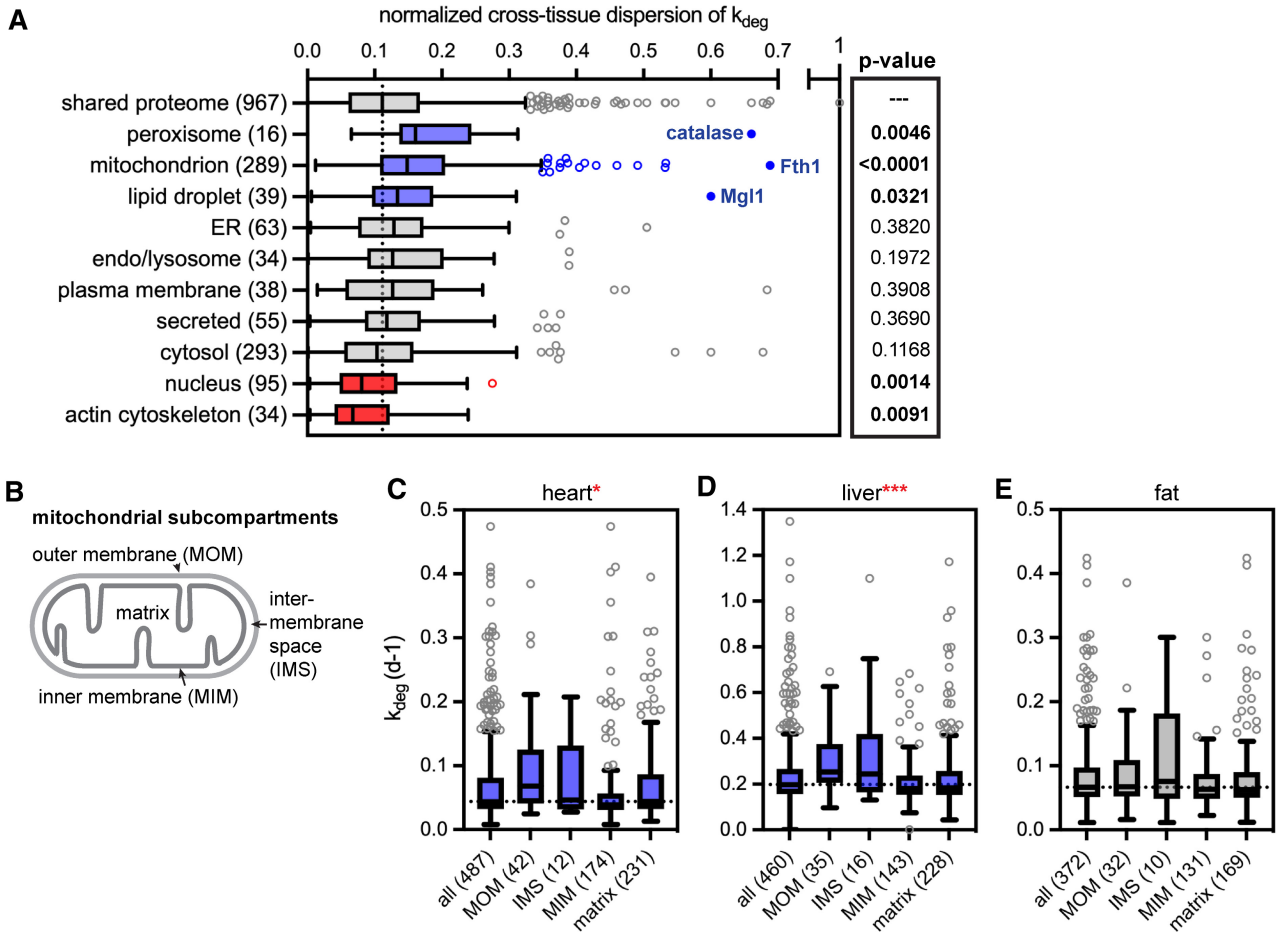
Cross‐tissue dispersion analysis of protein turnover rates indicates nonrandom variability in protein turnover ACross‐tissue dispersion analysis of *k*_deg_ values across heart, liver, and fat by cellular organelle. Box (Tukey) plot center line indicates median; box limits indicate 25^th^ to 75^th^ percentiles; whiskers indicate 1.5× interquartile range; points indicate outlier values. Blue indicates a subset has a significantly elevated median dispersion of *k*_deg_ values across tissues; red indicates a subset has a significantly decreased dispersion of *k*_deg_ values across tissues (Mann–Whitney test). The peroxisomal enzyme catalase, the mitochondrial resident protein Fth1, and the lipid droplet enzyme MGL1 are highlighted as residents of each organelle with the most extremely variable lifetimes across tissues.B–EAnalysis of *k*_deg_ rates across mitochondrial subcompartments. MOM: mitochondrial outer membrane; IMS: intermembrane space; MIM: mitochondrial inner membrane. Numbers indicate total proteins detected in each subcompartment. Proteins of the MOM and IMS turn over significantly faster than proteins of the MIM and matrix in the heart (C) and liver (D). Box (Tukey) plot center line indicates median; box limits indicate 25^th^ to 75^th^ percentiles; whiskers indicate 1.5× interquartile range; points indicate outlier values. *indicates *P* < 0.05; ***indicates *P* < 0.001. In contrast, all mitochondrial subcompartments turn over at similar rates in the white adipose tissue (E) (Kruskal–Wallis test). See also Dataset EV6.

**Figure 6:**
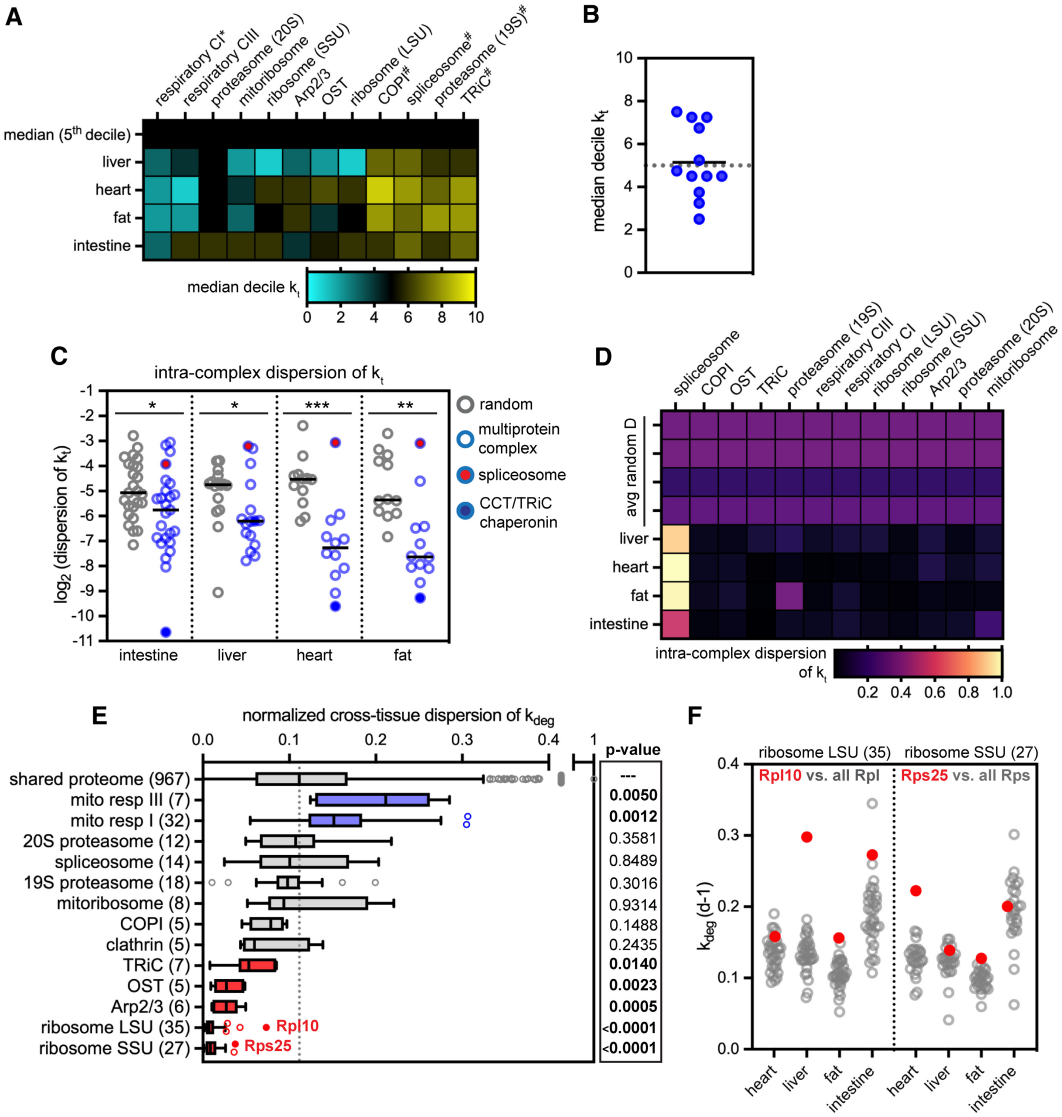
Analysis of protein turnover for subunits of multiprotein complexes The median decile of *k*_t_ for each multiprotein complex with at least five subunits detected. *indicates complexes that turn over significantly more slowly, and ^#^indicates complexes that turn over significantly faster than the proteome median in all tissues.Median decile *k*_t_ for multiprotein complexes that turn over significantly faster than the proteome median in all tissues. Median decile *k*_t_ for multiprotein complexes (black bar) does not deviate from the proteome median (5^th^ decile).Intracomplex dispersion was computed for complexes with at least five subunits detected (blue) and a random dispersion value was calculated by computing dispersion for an equivalent number of randomly chosen proteins (gray). Between 12 and 25 multiprotein complexes analyzed per tissue (see Source Data). Black bar, median. Multiprotein complexes exhibit a significantly lower intracomplex dispersion than would be expected by chance (*P < 0.05; **P < 0.01; ***P < 0.001, Mann–Whitney test). The spliceosome (red) is an outlier with high intracomplex dispersion; the TRiC/chaperonin complex (solid blue) is an outlier with low intracomplex dispersion.Heatmap of intracomplex dispersion across tissues for 12 multiprotein complexes with at least five subunits detected in all tissues.Analysis of normalized cross‐tissue dispersion of *k*_deg_ by multiprotein complex compared across liver, heart, and fat datasets. *P*‐values indicate significance of deviation from proteome mean (Mann–Whitney test). While the small and large subunits of the ribosome have extremely low cross‐tissue dispersion, outlier subunits Rps25 and Rpl10 have more variability across tissues (solid red). Box (Tukey) plot center line indicates median; boxes indicate 25^th^ to 75^th^ percentiles; whiskers indicate 1.5× interquartile range; points indicate outlier values.
*k*_deg_ values of ribosome subunits. See also Dataset EV7. Source data are available online for this figure.

To evaluate the extent of variability in *k*_deg_ across tissues, we determined the normalized cross‐tissue dispersion (*D*) of *k*_deg_ for the 967 proteins shared across the liver, fat, and heart datasets (see Materials and Methods; Appendix Fig S14; Dataset EV7). We then used this metric to dissect variability in protein lifetime across tissues, focusing on constituents of cellular organelles (Fig 5), multiprotein complexes (Fig 6) and pathways (Appendix Fig S15).

Among proteins that are residents of specific subcellular organelles, components of the actin cytoskeleton and residents of the nucleus have much less variable lifetimes than the proteome as a whole (Fig 5A and B). In contrast, constituents of peroxisomes, lipid droplets, and mitochondria have significantly more variable lifetimes across tissues than the proteome as a whole (Fig 5A; Appendix Fig S16), implying that the degradative flux of these organelles varies from tissue to tissue. These organelles can be degraded by specialized variants of autophagy termed pexophagy (Dunn *et al*, 2005), lipophagy (Singh *et al*, 2009), and mitophagy (Youle & Narendra, 2011), respectively.

Peroxisomes play major roles in lipid catabolism, and their biogenesis is induced by signaling through peroxisome proliferator agonist receptors (PPARs) and other mechanisms. Upon removal of biogenesis‐promoting signals, excess peroxisomes are degraded by pexophagy (Monastyrska & Klionsky, 2006). This process was first documented in the liver; consistently, we observe rapid turnover of peroxisomal proteins in this tissue. Our data indicate that peroxisomes are also degraded rapidly in the intestine but are degraded more slowly in the heart and adipose tissue (Appendix Fig S16).

Lipid droplets (LDs) are the major intracellular sites of lipid storage. In response to nutrient deprivation, LDs mobilize lipids either by lipolysis to generate fatty acids or by lipophagy, which involves delivery of both the protein and lipid components of LDs to the lysosome (Zechner *et al*, 2017). Lipophagic flux is high in the liver (Singh *et al*, 2009), and we observe rapid degradation of LD proteins in this organ (Appendix Fig S16). In contrast, LD proteins are longer lived in the white adipose tissue and in the heart. This is somewhat unexpected, as adipose tissue rapidly mobilizes free fatty acids when nutrients are low (Lafontan & Langin, 2009), while heart tissue depends on fat oxidation for energy (Pascual & Coleman, 2016). This outcome indicates that lipophagic flux is lower in these tissues and suggests that fatty acids are instead mobilized from LDs by lipolysis while sparing LD‐resident proteins from turnover.

Mitochondria are long‐lived organelles in many tissues, including the brain (Price *et al*, 2010; Fornasiero *et al*, 2018), heart (Lau *et al*, 2016), and skeletal muscle (Bomba‐Warczak *et al*, 2021; Krishna *et al*, 2021). Consistent with these recent studies, we find that mitochondria are long‐lived in the heart (median mitochondrial protein *t*_1/2_ of 18.1 days vs. 5.8 days for total proteome). Mitochondria are also long‐lived in the white adipose tissue (median mitochondrial protein *t*_1/2_ of 10.5 days vs. 6.8 days for total proteome). Surprisingly, however, mitochondria turn over more rapidly in the liver (median mitochondrial protein *t*_1/2_ of 3.5 days vs. 2.9 days for total proteome; Appendix Fig S16). This finding suggests major differences in mitochondrial regulation and function in this organ but is consistent with a previous report of high mitophagy flux in the liver using an *in vivo* reporter system (McWilliams *et al*, 2016).

We achieved high coverage of the mitochondrial proteome, making it possible to inspect the turnover of mitochondrial subcompartments across tissues (Fig 5B–E). Proteins of the mitochondrial outer membrane (MOM) and the intermembrane space (IMS) generally turn over more rapidly than proteins of internal compartments such as the mitochondrial inner membrane (MIM) and the matrix. This disparity is most apparent in the heart and liver (Fig 5C and D). A previous analysis of protein lifetimes in the brain similarly reported more rapid turnover of MOM proteins (Fornasiero *et al*, 2018). Notably, the MOM and IMS are accessible to the cytosol while the MIM and matrix are sequestered. These data suggest that selective degradation of MOM/IMS proteins occurs at a significant rate in many tissues, including the heart, liver, and brain (Fornasiero *et al*, 2018). This could be achieved by extraction and delivery to the proteasome, piecemeal autophagy, and/or sequestration into mitochondrial‐derived vesicles (Winter & Becker, 2021). In contrast, all mitochondrial subcompartments exhibit coherent turnover in white adipose tissue (Fig 5E). This could indicate that mitochondria are degraded more frequently by organellar autophagy (mitophagy) than by selective degradation of mitochondrial components in this tissue. Interestingly, mitophagy plays major roles in the differentiation and maintenance of white adipocytes, which characteristically have lower numbers of mitochondria (Altshuler‐Keylin *et al*, 2016). Altogether, our data indicate that both the overall flux of mitochondrial turnover and the mechanisms used to achieve turnover vary across tissues.

### Multiprotein complex subunits have coherent lifetimes within and across tissues

It has been suggested that participation in stable multiprotein complexes might protect proteins from degradation and extend protein half‐life (McShane *et al*, 2016; Mallik & Kundu, 2018). We evaluated the degradation kinetics of 12 multiprotein complexes for which at least five subunits were detected in all four tissues and found that in general, multiprotein complex subunits do not exhibit significantly lower degradation rates than the proteome median (Fig 6A and B). This indicates that participation in a stable multiprotein complex is not sufficient to dramatically extend protein lifetime compared to the proteome average. It is important to note, however, that this steady‐state measurement cannot determine whether nascent complex subunits are selectively degraded if they fail to assemble correctly after synthesis (McShane *et al*, 2016).

Some multiprotein complexes have been reported to exhibit coherent subunit turnover, perhaps reflecting their stable association from biogenesis to degradation (Mathieson *et al*, 2018). To determine whether complex subunits exhibit more similar turnover rates than would be expected by random chance, we calculated the intratissue *k*_t_ dispersion (*d*) for multiprotein complexes for which at least five subunits were detected (12–24 complexes per tissue). For comparison, we calculated *d* for an equivalent number of randomly chosen proteins. Comparing these values indicated that most multiprotein complexes turn over coherently (Fig 6C and D). Consistent with a previous report (Mathieson *et al*, 2018), we also find that the CCT/TriC chaperonin complex is an outlier whose subunits have extremely consistent turnover rates (low *d*, Fig 6C, solid blue). Other multiprotein complexes with highly coherent turnover include the ribosome, proteasome, oligosaccharyltransferase (OST) complex, and the mitochondrial respiratory chain complexes (Fig 6D). In striking contrast, the spliceosome is an outlier whose components have widely varying turnover rates (high *d*; Fig 6C, solid red; Fig 6D). Since spliceosome assembly is cyclical and coupled with catalytic activity (Matera & Wang, 2014), we asked whether individual spliceosome subcomplexes exhibit coherent turnover rates. We evaluated spliceosome subcomplexes in the intestine and liver, where we had coverage of at least five subunits of the exon junction complex, the U2 subcomplex, and the A complex. Interestingly, even within these smaller subcomplexes we saw high intrasubunit dispersion in turnover rates (Appendix Fig S17). We speculate that this unusually high intracomplex variability reflects the transience of interactions between many spliceosomal subunits; it has been estimated that > 30 proteins are exchanged during some catalytic steps of the splicing cycle (Hegele *et al*, 2012).

We next asked whether multiprotein complex lifetimes are consistent or variable across tissues by calculating the cross‐tissue *k*_deg_ dispersion (*D*) of individual subunits across the liver, heart, and fat. Components of mitochondrial respiratory chain complexes were the only complex subunits that had significantly higher *D* than the proteome median (Fig 6E), which is likely due to the dramatic differences in mitochondrial lifetime across tissues (Fig 5). Apart from these outliers, other multiprotein complex subunits had average or significantly lower than average values of *D*. We noted that the small and large subunits of the ribosome had extremely low *D* values (Fig 6E), and that the stability of the small and large subunits tracked very closely with each other (Fig 6F). The ribosome also has a very consistent half‐life across fibroblasts derived from a range of mammalian species (Swovick *et al*, 2020), indicating that ribosome turnover is very tightly controlled by unknown mechanisms. However, Rpl10 and Rps25 had much more variable *k*_deg_ values than other ribosomal proteins (Fig 6E and F). Interestingly, Rpl10 association is a key late regulatory step in large subunit biogenesis (Bussiere *et al*, 2012), and Rps25 is incorporated only in a subset of ribosomes that are endowed with unique translational specificity (Shi *et al*, 2017). Rpl10 turns over faster than other large subunit components in the liver and intestine, while Rps25 turns over faster than most small subunit components in the heart (Fig 6F). These data suggest nodes of ribosome biogenesis control that vary across tissues.

## Discussion

Here we report the development of TRAIL, a multiplexed ^15^N isotope‐labeling workflow that enables simultaneous measurements of protein lifetime and cellular lifetime from the same tissue. To our knowledge, this is the first study to advance a method for deriving cell turnover rates from ^15^N labeling. Mass spectrometric quantification of isotope incorporation into nucleosides provides high precision, sensitivity, and accuracy. In contrast to other frequently used approaches for quantifying cell turnover, ^15^N has no detectable toxicity, even through multiple generations of continuous labeling in mice (McClatchy *et al*, 2007; Savas *et al*, 2012), opening the possibility of extending TRAIL to accurately define the turnover rates of slowly proliferating cell types.

By sensitively measuring cell turnover and protein turnover in parallel, TRAIL adds a critical layer of context to analysis of proteostasis. We have unambiguously determined that protein lifetimes vary widely across tissues, and that sequence features as well as cell turnover and additional environmental factors shape protein lifetime. Our data suggest that long‐lived proteins experience a very different life cycle in postmitotic versus proliferative tissues. Cell and protein turnover flux occur at comparable rates in the proliferative intestine, such that the proteome is renewed roughly every 3 days as the epithelium renews (Fig 4). In contrast, protein turnover outpaces cell turnover in slowly proliferative tissues, and lifetimes of individual proteins spread over a broader dynamic range. In this context, protein turnover is both sequence‐selective (Fig 3) and coordinated across multiprotein complex subunits (Fig 6). We speculate that only in this context would long‐lived proteins and complexes meaningfully “age”—meaning that they accumulate oxidative damage, misfold, and lose their function, which would in turn lead to age‐linked tissue dysfunction. We have also uncovered evidence that the rate of organelle degradation, perhaps by autophagy of peroxisomes, lipid droplets, and mitochondria, varies widely across tissues (Fig 5). Why do some proteins and organelles turn over at such variable rates? It is possible that protein damage occurs more rapidly in some tissues than in others, perhaps linked to the variable rate of production of reactive oxygen species and other damaging agents during normal cellular metabolism. A second, nonexclusive possibility is that the activity and/or selectivity of protein folding and/or degradation machineries varies across cell types and tissues (Vonk *et al*, 2020). Intriguingly, *in vivo* reporters of the proteasome and of autophagy do suggest variable flux across tissues (Mizushima *et al*, 2004; Jenkins *et al*, 2020).

In the future, we anticipate that TRAIL can be applied to explore the consequences of aging and disease on tissue homeostasis. However, there are some limitations of our approach to consider. Our continuous labeling approach must assume maintenance of homeostatic balance over the time frame of the experiment—an assumption that is more likely to be valid over shorter timescales and in healthy tissues, but may not prove to be true over longer timescales or in diseased tissues. Our approach also does not address the contribution of different cell types to bulk measurements of cell turnover or protein turnover. Importantly, these bulk tissue measurements report a weighted average of the turnover behavior of the most abundant cell types in each tissue. We surmise that our data most accurately reflect the turnover of proteins that are broadly expressed in most cell types of the tissues analyzed. Overall, we were able to profile the 1,500–3,000 most abundant proteins per tissue, and we find very few cell type‐specific proteins in these datasets (Appendix Fig S13). Future studies may involve computational deconvolution or sorting of individual abundant cell types from tissues of interest in order to generate cell type‐resolved maps of cell and proteome lifetime.

## Materials and Methods

### Reagents and Tools table

**Table:** 

Reagent/Resource	Reference or Source	Identifier or Catalog Number
**Experimental Models**		
C57BL/6J (*M. musculus*)	Jackson Labs	n/a
**Chemicals, Enzymes and other reagents**		
^14^N‐labeled mouse chow	Silantes	231004650
^15^N‐labeled mouse chow	Silantes	231304650
TRIzol	Invitrogen	15596026
Benzonase	EMD Millipore	70664
Shrimp alkaline phosphatase	NEB	M0371
Phosphodiesterase I	Abnova	P5263
Pierce™ Protein BCA Assay	Thermo Fisher	23225
TMTpro 16plex labeling kit	Thermo Fisher	A44520
S‐Trap Columns	Protifi	
Spin‐X UF Concentrators	Corning	431478
**Software**		
Proteome Discoverer	Thermo Scientific	
XCalibur	Thermo Scientific	
R Version 4.1.0	https://www.r‐project.org/	
**Other**		
Fusion Lumos Tribrid MS	Thermo Fisher	
Q‐Exactive MS	Thermo Fisher	

### Methods and Protocols

#### Metabolic labeling of mice and tissue isolation

We designed a 6‐timepoint, 32‐day SILAM labeling time course (0, 2, 4, 8, 16, and 32 days of labeling) with a total of three animals of both sexes per labeled timepoint, and two animals for the day 0 (unlabeled) timepoint. Time courses were performed in male and female wild‐type C57Bl/6 mice at approximately 9 weeks of age. ^14^N and ^15^N mouse chow was obtained from Silantes. Animals were first habituated to the chow formulation by feeding ^14^N (normisotopic) food for 1 week and monitoring animal weight. Animals maintained normal weight through the duration of the time course. Animals were then transitioned to ^15^N chow throughout the labeling period (roughly 3 g/animal/day). Animals were then sacrificed by CO_2_ inhalation followed by cervical dislocation, followed by tissue dissection and flash freezing by submersion in liquid nitrogen. These animal experiments were performed in compliance with relevant ethical regulations and with approval by the Institutional Animal Care and Use Committee at UCSF (IACUC protocol number AN178187, PI: A.B.).

#### Protein extraction and sample preparation for LC–MS/MS

##### Protein extraction

Approximately 30 mg of frozen tissue was excised on dry ice with a clean razorblade and placed in a fresh tube. 100 μl of protein extraction buffer (PEB: 5% SDS, 100 mM TEAB, protease and phosphatase inhibitors, pH ~ 7) was added to the tube. The tissue was rapidly minced with clean dissection scissors on ice for 30–60 s until no large pieces remained. PEB was added to bring the final volume to 600 μl, then the sample was transferred to a Dounce homogenizer. The sample was homogenized for ~ 40 strokes with the tight pestle, then was transferred to a clean microcentrifuge tube. The sample was then probe sonicated at 4C (10% amplitude, 10 s, 2 cycles) before being centrifuged (21,000 *g*, 11 min, 4C). The supernatant was transferred to a clean tube, and aliquots were separated for proteomics and protein quantification by microBSA assay (Pierce).

##### Trypsinization

Samples were diluted to 1 mg/ml in 5% SDS, 100 mM TEAB, and 25 μg of protein from each sample was reduced with dithiothreitol to 2 mM, followed by incubation at 55°C for 60 min. Iodoacetamide was added to 10 mM and incubated in the dark at room temperature for 30 min to alkylate the proteins. Phosphoric acid was added to 1.2%, followed by six volumes of 90% methanol, 100 mM TEAB. The resulting solution was added to S‐Trap micros (Protifi), and centrifuged at 4,000 *g* for 1 min. The S‐Traps containing trapped protein were washed twice by centrifuging through 90% methanol, 100 mM TEAB. 1 μg of trypsin was brought up in 20 μl of 100 mM TEAB and added to the S‐Trap, followed by an additional 20 μl of TEAB to ensure the sample did not dry out. The cap to the S‐Trap was loosely screwed on but not tightened to ensure the solution was not pushed out of the S‐Trap during digestion. Samples were placed in a humidity chamber at 37°C overnight. The next morning, the S‐Trap was centrifuged at 4,000 *g* for 1 min to collect the digested peptides. Sequential additions of 0.1% TFA in acetonitrile and 0.1% TFA in 50% acetonitrile were added to the S‐trap, centrifuged, and pooled. Samples were frozen and dried down in a Speed Vac (Labconco) prior to TMTpro labeling.

##### TMT labeling

Samples were reconstituted in TEAB to 1 mg/ml, then labeled with TMTpro 16plex reagents (Thermo Fisher) following the manufacturers protocol. Briefly, TMTpro tags were removed from the −20°C freezer and allowed to come to room temperature, after which acetonitrile was added. Individual TMT tags were added to respective samples, and the reaction was allowed to occur at room temperature for 1 h. 5% hydroxylamine was added to quench the reaction, after which the samples for each experiment were combined into a single tube. Since we performed quantitation on the unlabeled peptides, 0 day samples were added to four of the unused channels, increasing the signal for the unlabeled peptides. TMTpro‐tagged samples were frozen, dried down in the Speed Vac, and then desalted using homemade C18 spin columns to remove excess tag prior to high pH fractionation.

##### High pH fractionation

Homemade C18 spin columns were activated with two 50‐μl washes of acetonitrile via centrifugation, followed by equilibration with two 50‐μl washes of 0.1% TFA. Desalted, TMTpro‐tagged peptides were brought up in 50 μl of 0.1% TFA and added to the spin column. After centrifugation, the column was washed once with water, then once with 10 mM ammonium hydroxide. Fractions were eluted off the column with centrifugation by stepwise addition of 10 mM ammonium hydroxide with the following concentrations of acetonitrile: 2, 3.5, 5, 6.5, 8, 9.5, 11, 12.5, 14, 15.5, 17, 18.5, 20, 21.5, 27, and 50%. The 16 fractions were concatenated down to 8 by combining fractions 1 and 9, 2 and 10, 3 and 11, etc. Fractionated samples were frozen, dried down in the Speed Vac, and brought up in 0.1% TFA prior to mass spectrometry analysis.

#### LC–MS/MS analysis

##### Data collection

Peptides from each fraction were injected onto a homemade 30‐cm C18 column with 1.8‐μm beads (Sepax), with an Easy nLC‐1200 HPLC (Thermo Fisher), connected to a Fusion Lumos Tribrid mass spectrometer (Thermo Fisher). Solvent A was 0.1% formic acid in water, while solvent B was 0.1% formic acid in 80% acetonitrile. Ions were introduced to the mass spectrometer using a Nanospray Flex source operating at 2 kV. The gradient began at 3% B and held for 2 min, increased to 10% B over 7 min, increased to 38% B over 94 min, then ramped up to 90% B in 5 min and was held for 3 min, before returning to starting conditions in 2 min and re‐equilibrating for 7 min, for a total run time of 120 min. The Fusion Lumos was operated in data‐dependent mode, employing the MultiNotch Synchronized Precursor Selection MS3 method to increase quantitative accuracy (McAlister *et al*, 2014). The cycle time was set to 3 s. Monoisotopic Precursor Selection (MIPS) was set to Peptide. The full scan was done over a range of 400–1,500 *m/z*, with a resolution of 120,000 at *m/z* of 200, an AGC target of 4e5, and a maximum injection time of 50 ms. Peptides with a charge state between 2 and 5 were picked for fragmentation. Precursor ions were fragmented by collision‐induced dissociation (CID) using a collision energy of 35% and an isolation width of 1.0 *m/z*. MS2 scans were collected in the ion trap with an AGC target of 1e4 and a maximum injection time of 35 ms. MS3 scans were performed by fragmenting the 10 most intense fragment ions between 400–2,000 *m/z*, excluding ions that were 40 *m/z* less and 10 *m/z* greater than the precursor peptide, using higher energy collisional dissociation (HCD). MS3 ions were detected in the Orbitrap with a resolution of 50,000 at *m/z* 200 over a scan range of 100–300 *m/z*. The isolation width was set to 2 Da, the collision energy was 60%, the AGC was set to 1e5, and the maximum injection time was set to 100 ms. Dynamic exclusion was set to 45 s.

##### Data analysis

Raw data were searched using the SEQUEST search engine within the Proteome Discoverer software platform, version 2.4 (Thermo Fisher), using the Uniprot mouse database (downloaded January 2020). Trypsin was selected as the enzyme allowing up to two missed cleavages, with an MS1 mass tolerance of 10 ppm, and an MS2 mass tolerance of 0.6 Da. Carbamidomethyl on cysteine, and TMTpro on lysine and peptide N terminus were set as a fixed modifications, while oxidation of methionine was set as a variable modification. Percolator was used as the FDR calculator, filtering out peptides which had a q‐value greater than 0.01. Reporter ions were quantified using the Reporter Ions Quantifier node, with an integration tolerance of 20 ppm, and the integration method being set to “most confident centroid”; the average reporter ion signal‐to‐noise threshold was set to 5. Protein abundances were calculated by summing the signal to noise of the reporter ions from each identified peptide, while excluding any peptides with an isolation interference of > 30%, or SPS matches < 65%.

##### Kinetic model

The kinetic model applied in this study has been previously described (Welle *et al*, 2016). Briefly, we are assuming that protein synthesis is a zero order process, occurs at a constant fractional rate, and that that the total protein concentration of each cell does not change during the experimental time course. The dilution of the protein pool due to cell division can be modeled as a first‐order exponential process. Thus, the fractional turnover of unlabeled proteins during the labeling time course can be regarded as a first‐order kinetic process that can be modeled based on the following exponential equation:

(1) $$\text{fraction unlabeled protein}\;\left(t\right)=e^{-k_{t}*t}$$

And:

$$k_{t}=k_{\deg}+k_{\mathrm{div}}$$

where $k_{t}$ is the clearance rate (observed rate of fractional labeling), $k_{\deg}$ is the rate of protein degradation and $k_{\mathrm{div}}$ is the rate of cell division.

The determination of *k*_t_ values were conducted as previously described (Welle *et al*, 2016) using the decay of the TMT reporter signals of unlabeled proteins. Protein‐level TMT reporter abundances for unlabeled proteins for each replicate experiment were first normalized by dividing by the intensity of the t0 reporter and then the replicate experiments were aggregated in a single kinetic curve. In fitting the exponential decay curves of the unlabeled protein signals, a constant fractional baseline at infinite time was incorporated in the fitting equation. The equation used for fitting the curves was therefore: $\text{intensity}=\text{baseline}+\left(1-\text{baseline}\right)*e^{-k_{t}*\text{time}}$. The goodness of fit for least squares fits were assessed by determining the *R* ^2^, *P*‐value and *t*‐statistic of the fits (see Dataset EV1). For subsequent analyses, only protein *k*_t_ measurements that were obtained from all three replicate experiments, incorporated data from four or more peptide spectral matches (PSMs), and had *t*‐statistic values greater than three were considered.

#### Nucleic acid extraction and sample preparation for LC–MS/MS

##### Genomic DNA extraction

Approximately 30 milligrams of frozen tissue was excised on dry ice with a clean razorblade and placed in a fresh tube. 100 μl of TRIzol reagent (Invitrogen) was added and the tissue was rapidly minced with clean dissection scissors on ice for 30–60 s until no large pieces remained. An additional 400 μl of TRIzol was added, and the sample was then transferred to a Dounce homogenizer. The tissue was subjected to ~40 strokes with the tight pestle until smooth, then transferred back to the original tube. The sample was incubated for at least 5 min before the addition of 100 μl chloroform followed by mixing and a further 3 min of incubation. The sample was then centrifuged (12,000 *g*, 15 min, 4C) and the upper RNA‐containing aqueous layer was discarded. 150 μl of absolute ethanol was added to the remaining sample, then inverted several times to mix. After 3 min of incubation at room temperature, the sample was centrifuged (2,000 *g*, 5 min, 4C). The protein‐containing supernatant was removed, then the DNA‐containing pellet was resuspended in 500 μl of absolute ethanol and incubated for 30 min. The sample was then centrifuged (2,000 *g*, 5 min, 4C), and the supernatant discarded. Sequential washes were then repeated with 95, 85, and 75% ethanol, after which the pellet was air‐dried for 5–10 min. The pellet was then resuspended in 200 μl nuclease‐free water (Ambion) at 56C, then incubated at 56C with shaking for 30 min to resuspend the pure DNA. The sample was centrifuged (12,000 *g*, 10 min, 4C), then the supernatant containing pure DNA was moved to a clean tube. DNA concentration was determined with a NanoDrop spectrophotometer.

##### Digestion of genomic DNA to short oligonucleotides

3–5 micrograms of pure genomic DNA was diluted to a 50 μl volume in nuclease‐free water, then combined with 50 μl of 2× Dinucleotide Buffer (DB: 5 mU/μl benzonase, 40 mU/μl shrimp alkaline phosphatase, 20 mM Tris pH 7.9, 100 mM NaCl, 20 mM MgCl_2_). Samples were incubated overnight at 37C. Spin‐X UF Concentrators (Corning) were rinsed with 200 μl buffer (20 mM Tris pH 7.9, 100 mM NaCl, 20 mM MgCl_2_), then samples were applied and centrifuged through (12,000 *g*, 5 min, RT). The eluate was collected for analysis.

##### Digestion of genomic DNA to mononucleosides

We extracted mononucleosides from genomic DNA similarly to a previously described method (Quinlivan & Gregory, 2008) with some modifications. 1–3 micrograms of pure genomic DNA was diluted to a 50 μl volume in nuclease‐free water, then combined with 50 μl of 2× Mononucleoside Buffer (MB: 5 mU/μl benzonase, 40 mU/μl shrimp alkaline phosphatase, 60 uU/μl phosphodiesterase I, 20 mM Tris pH 7.9, 100 mM NaCl, and 20 mM MgCl_2_). Samples were incubated overnight at 37C. Spin‐X UF Concentrators (Corning) were rinsed with 200 μl buffer (20 mM Tris pH 7.9, 100 mM NaCl, 20 mM MgCl_2_), then samples were applied and centrifuged through (12,000 *g*, 5 min, RT). The eluate was collected for analysis.

#### Mononucleoside and dinucleoside LC–MS/MS

Mononucleotide analyses were carried out by adapting a previously described method (Su *et al*, 2014) using a Dionex Ultimate 3000 UHPLC coupled with a Q Exactive Plus mass spectrometer (Thermo Scientific). After purification, analytes were separated on a Hypersil Gold 2.1 × 150 mm column, protected by a 2.1 × 10 mm Hypersil Gold guard column (Thermo Scientific). The mobile phases were A: 0.1% formic acid in water, and B: 0.1% formic acid in acetonitrile. The flow rate was set to 400 μl/min, and the column oven was set to 36°C. 10 μl of each sample was injected, and the analytes were eluted using the following gradient: 0 min, 0% B; 6 min, 0% B; 8.5 min, 80% B; 9.5 min, 80% B; 10 min, 0% B; 13 min, 0% B. The Q Exactive Plus was operated in positive mode with a heated electrospray ionization (HESI) source. The spray voltage was set to 3.5 kV, the sheath gas flow rate was set to 40, and the auxiliary gas flow rate set to 7, while the capillary temperature was set to 320°C. A parallel reaction monitoring (PRM) method was used to quantify the unlabeled nucleotide, along with all of its N15 isotopes in a single scan. This was accomplished by using wide (8 *m/z*) isolation widths when selecting the nucleotides for fragmentation. By employing this method, we were able to quantify the level of labeling by looking at the intensity of each N15‐labeled base in the MS2 scan. Fragment ions were detected in the Orbitrap with a resolution of 70,000 at *m/z* 200. Using a high‐resolution MS2 scan allowed us to resolve N15 and C13 isotopes. Peak areas from the fragment ions were extracted with a 10 ppm mass tolerance using the LC Quan node of the XCalibur software (Thermo Scientific).

Dinucleotide analyses were carried out using the same instrumentation, column, mobile phases, column temperature, and flow rate employed by the mononucleotide experiments. The gradient was changed to optimize dinucleotide separation as follows: 0 min, 5% B; 0.5 min, 5% B; 2.5 min, 90% B; 3.25 min, 90% B; 3.5 min, 5% B; 5.5 min, 5% B. The Q Exactive Plus was operated using the same tune settings as the mononucleotide experiment. However, instead of a PRM method, a full scan method from 500–650 *m/z* was developed to quantify the dinucleotides dCdC, TT, dAdA, and dGdG, along with their corresponding N15 isotopes. Precursor ions were detected in the Orbitrap with a resolution of 140,000 at *m/z* 200, using the high‐resolution MS1 scan to try to separate N15 and C13 isotopes as much as possible. Peak areas from the fragment ions were extracted with a 10 ppm mass tolerance using the LC Quan node of the XCalibur software (Thermo Scientific).

#### Measurement of *k*_div_

To accurately measure rates of cell division (*k*_div_) while factoring in the effects of incomplete labeling and nucleotide recycling, we considered the time‐dependent labeling patterns of mononucleotides and dinucleotides derived from genomic DNA. Upon initiation of ^15^N labeling, newly synthesized DNA strands can incorporate nucleotides from a precursor pool with potentially complex mixture of partially labeled species (Appendix Fig S8A). For example, a newly incorporated deoxyadenosine (dA) can be derived from fully ^15^N‐labeled nucleotides derived from the dietary source, partially labeled species (containing one to four ^15^N atoms) derived by biosynthesis from incompletely labeled ^15^N precursors, and completely unlabeled nucleotides derived from recycling. As an example, a typical labeling pattern for dA from one of our intestine samples is shown in Appendix Fig S10B showing the shift in the isotopologue distribution over time. After correcting for the natural isotopic distribution, the peaks with heavier nonmonoisotopic masses (+1, +2, +3, etc.) can be assumed to have been derived from newly synthesized strands. However, the monoisotopic peak (0) can potentially have been derived from both the original unlabeled strand, as well as newly synthesized strands that had incorporated recycled unlabeled nucleotides. Therefore, it may not be possible to accurately determine the ratio of new to old strands (and hence *k*_div_) from the mononucleotide data alone. The labeling pattern of dinucleotides (dAdA) resolves this ambiguity. The isotopologue distribution of labeled (nonmonoisotopic) peaks in the dinucleotides spectra are dependent on the composition of the nucleotide precursor pool. The red envelope depicted in the dAdA spectra is the pattern that would be expected if the precursor pool was composed solely of the labeled (nonmonoisotopic) species observed in the corresponding mononucleotide spectra (i.e., new strands did not contain any recycled unlabeled nucleotides and the monoisotopic peaks observed in the mononucleotide spectra were derived solely from old strands). If a significant fraction of the monoisotopic peaks observed in the mononucleotide spectra represents recycled nucleotides within new strands, then the isotopologue distribution of labeled nucleotides would shift accordingly (Appendix Fig S10C). Through regression analyses, we determined that within all tissues and timepoints analyzed in this study, the isotopologue distributions of the dinucleotide data could be best modeled based on the assumption that newly synthesized strands had very low levels of fully unlabeled nucleotides. Hence, the fractional population of labeled nonmonoisotopic peaks within dinucleotide and mononucleotide data were consistent with each other (Appendix Fig S10D) and could be used to determine the fractional population of new strands. For each tissue, fractional labeling of mononucleotide and dinucleotides for all four bases were combined and the aggregated dataset was fit to a single exponential equation to determine first‐order rate constant for division (*k*_div_). These data appear in Dataset EV4.

#### Analysis of proteomic data

##### Quality filtering and analysis of *k*_t_ values

Proteomic data were acquired in the form of TMT replicates containing full 6‐timepoint time courses. Within each TMT replicate, proteins were filtered to retain only those detected with at least three peptide spectral matches (PSMs) in all timepoints. Proteins that met these criteria were then filtered within each TMT replicate based on goodness of fit using the *t*‐statistic. The *t*‐statistic is equal to the turnover rate (*k*_t_) divided by the standard error of that value. This metric determines to what extent measurement error influences *k*_t_. We applied a minimum *t*‐statistic cutoff of 3, meaning that the magnitude of the turnover rate *k*_t_ is at least three times the magnitude of the standard error. Between 50 and 63% of detected proteins passed these coverage and goodness‐of‐fit criteria (Appendix Fig S3). Along with the sample size, the *t*‐statistic can be used to determine a *P*‐value that indicates the probability that the turnover rate reported has a meaningful nonzero value. The *k*_t_, standard error, *t*‐statistic, and *P*‐value for each protein are reported in Dataset EV1. The *k*_t_, standard error, and sample size were used to perform per‐protein statistical tests across tissues, to identify proteins with significantly different turnover kinetics between tissues. These data are reported in Dataset EV1.

Filtered *k*_t_ values for each tissue were separated into deciles. Proteins in the top decile (fastest *k*_t_) and bottom decile (slowest *k*_t_) were subjected to gene ontology analysis to identify biological processes (GO:BP) and cellular components (GO:CC) that were overrepresented, using the Gprofiler tool (Raudvere *et al*, 2019). Redundant GO terms were filtered using ReVIGO (Supek *et al*, 2011), then subjected to hierarchical clustering and presented in heatmap format with cell values corresponding to the significance of enrichment for each term.

##### Analysis of protein sequence feature correlations with *k*_t_ values

For proteins whose *k*_t_ values passed the coverage and goodness‐of‐fit criteria described above, protein sequence features were evaluated as follows. Hydrophobicity was quantified by a grand average of the hydropathy (GRAVY) score (Kyte & Doolittle, 1982). Molar abundance of amino acid classes, isoelectric point, and molecular weight were extracted using Pepstats (Madeira *et al*, 2022; https://www.ebi.ac.uk/Tools/seqstats/emboss_pepstats/). The correlation of each of these parameters to *k*_t_ was evaluated by calculating the Spearman correlation coefficient. Intrinsically disordered regions (IDRs) were defined by identifying stretches of at least 40 amino acids having IUPRED2 (Mészáros *et al*, 2018) disorder scores > 0.5; IDRs of at least 40 amino acids in length have been previously shown to correlate with shorter protein lifetimes in cultured cells and in yeast (van der Lee *et al*, 2014; Fishbain *et al*, 2015). A validated list of mouse IDPs was sourced from the DisPROT database (Quaglia *et al*, 2021).

##### Determination and analysis of cell cycle‐corrected *k*_deg_ values

Cell cycle‐corrected protein *k*_deg_ values were determined by subtracting the cell doubling time (*k*_div_) for each tissue from the apparent protein turnover rate (*k*_t_) determined in that tissue. In the intestine, a significant proportion of the proteome had *Kdeg* rates very similar to *k*_div_. Gene ontology analyses of this subset of ~400 proteins was performed to identify biological processes (GO:BP) and cellular components (GO:CC) that were overrepresented, using Gprofiler (Raudvere *et al*, 2019). Redundant GO terms were filtered using ReVIGO (Supek *et al*, 2011), and a bubble plot of significance of enrichment versus similarity (semantic space) was generated using Prism (GraphPad), where bubble sizes correspond to the number of proteins mapped to a term. To analyze trends in turnover for intrinsically disordered proteins (IDPs), a list of curated and experimentally validated IDPs from the DisProt database (Quaglia *et al*, 2021) was cross‐referenced to *k*_deg_ values.

##### Cross‐tissue dispersion of *k*_deg_ values

Cross‐tissue dispersion (*D*) was calculated on a protein‐by‐protein basis for all proteins detected in the liver, heart, and fat tissues. *D = variance / mean*, where variance (V) is the average of the squared differences of each *k*_deg_ value from the mean *k*_deg_ value. Because *D* is normalized to the mean *k*_deg_ value, it is independent of the magnitude of *k*_deg_ (see Appendix Fig S14). *D* values are reported in Dataset EV7. Analysis of *D* by organelle was performed using annotations from MitoCarta (Rath *et al*, 2020) for mitochondrial proteins, from a recent proximity labeling study for lipid droplets (Bersuker *et al*, 2018), and manually curated annotations from UniProt for all other organelles. Only UniProt annotations that listed a specific organelle as the first affiliation were retained to limit multilocalizing proteins.

##### Intracomplex dispersion of *k*_deg_ values for multiprotein complexes

A mouse proteome multiprotein complex subunit annotation set from ComplexPortal (Meldal *et al*, 2018) was used to search for multiprotein complexes with at least five subunits detected in the liver, heart, intestine, or fat. Intracomplex dispersion for these complexes was determined by calculating the dispersion (*d = variance/mean*) of *k*_deg_ values for all subunits detected in a tissue.

##### Determination of relative protein abundance within tissues

To evaluate relative protein abundance within tissues, technical replicate unlabeled wild‐type (WT) samples from each multiplexed TMT run were first channel normalized, then the geometric mean was calculated to determine mean normalized intensities for each biological replicate. Protein abundance was then length‐normalized by dividing each protein's normalized intensity by the number of amino acids. Finally, samples were normalized for comparison across biological replicates by normalizing each channel to the maximum value detected. The geometric mean abundance was calculated by determining the geometric mean of the length‐ and channel‐normalized protein abundance. These relative abundance values were used to explore the relationship between protein abundance and protein half‐life (Fig 3; Appendix Fig S5).

## Author contributions

**John Hasper:** Data curation; formal analysis; investigation; methodology; writing – original draft; project administration. **Kevin Welle:** Data curation; software; formal analysis; validation; investigation; methodology. **Jennifer Hryhorenko:** Formal analysis; investigation; methodology. **Sina Ghaemmaghami:** Conceptualization; resources; data curation; software; formal analysis; supervision; validation; investigation; visualization; methodology; writing – original draft; project administration; writing – review and editing. **Abigail Buchwalter:** Conceptualization; resources; data curation; software; formal analysis; supervision; funding acquisition; validation; investigation; visualization; methodology; writing – original draft; project administration; writing – review and editing.

## Disclosure and competing interests statement

The authors declare that they have conflict of interest.

## Supporting information



Appendix S1

Dataset EV1

Dataset EV2

Dataset EV3

Dataset EV4

Dataset EV5

Dataset EV6

Dataset EV7

Source Data for Figure 3

Source Data for Figure 6

## Data Availability

LC–MS/MS data have been deposited in the ProteomeXchange Consortium via the PRIDE partner repository under the ID PXD033649 (http://www.ebi.ac.uk/pride/archive/projects/PXD033649).

## References

[msb202211393-bib-0001] Ahmad K , Henikoff S (2002) The histone variant H3.3 Marks active chromatin by replication‐independent nucleosome assembly. Mol Cell 9: 1191–1200 12086617 10.1016/s1097-2765(02)00542-7

[msb202211393-bib-0002] Altshuler‐Keylin S , Shinoda K , Hasegawa Y , Ikeda K , Hong H , Kang Q , Yang Y , Perera RM , Debnath J , Kajimura S (2016) Beige adipocyte maintenance is regulated by autophagy‐induced mitochondrial clearance. Cell Metab 24: 402–419 27568548 10.1016/j.cmet.2016.08.002 PMC5023491

[msb202211393-bib-0003] Asher E , Payne CM , Bernstein C (2009) Evaluation of cell death in EB V‐transformed lymphocytes using agarose gel electrophoresis, light microscopy and electron microscopy: II. Induction of non‐classic apoptosis (“Para‐apoptosis”) by tritiated thymidine. Leuk Lymphoma 19: 107–119 10.3109/10428199509059664 8574155

[msb202211393-bib-0004] Azimifar SB , Nagaraj N , Cox J , Mann M (2014) Cell‐type‐resolved quantitative proteomics of murine liver. Cell Metab 20: 1076–1087 25470552 10.1016/j.cmet.2014.11.002

[msb202211393-bib-0005] Bersuker K , Peterson CWH , To M , Sahl SJ , Savikhin V , Grossman EA , Nomura DK , Olzmann JA (2018) A proximity labeling strategy provides insights into the composition and dynamics of lipid droplet proteomes. Dev Cell 44: 97–112 29275994 10.1016/j.devcel.2017.11.020 PMC5764092

[msb202211393-bib-0006] Bomba‐Warczak E , Edassery SL , Hark TJ , Savas JN (2021) Long‐lived mitochondrial cristae proteins in mouse heart and brain. J Cell Biol 220: e202005193 34259807 10.1083/jcb.202005193 PMC8282663

[msb202211393-bib-0007] Bussiere C , Hashem Y , Arora S , Frank J , Johnson AW (2012) Integrity of the P‐site is probed during maturation of the 60S ribosomal subunit. J Cell Biol 197: 747–759 22689654 10.1083/jcb.201112131 PMC3373404

[msb202211393-bib-0008] Cook AJL , Gurard‐Levin ZA , Vassias I , Almouzni G (2011) A specific function for the histone chaperone NASP to fine‐tune a reservoir of soluble H3‐H4 in the histone supply chain. Mol Cell 44: 918–927 22195965 10.1016/j.molcel.2011.11.021

[msb202211393-bib-0009] D'Angelo MA , Raices M , Panowski SH , Hetzer MW (2009) Age‐dependent deterioration of nuclear pore complexes causes a loss of nuclear integrity in postmitotic cells. Cell 136: 284–295 19167330 10.1016/j.cell.2008.11.037 PMC2805151

[msb202211393-bib-0010] Darwich AS , Aslam U , Ashcroft DM , Rostami‐Hodjegan A (2014) Meta‐analysis of the turnover of intestinal epithelia in preclinical animal species and humans. Drug Metab Dispos 42: 2016–2022 25233858 10.1124/dmd.114.058404

[msb202211393-bib-0011] Dörrbaum AR , Kochen L , Langer JD , Schuman EM (2018) Local and global influences on protein turnover in neurons and glia. Elife 7: e34202 29914620 10.7554/eLife.34202 PMC6008053

[msb202211393-bib-0012] Drake JC , Peelor FF , Biela LM , Watkins MK , Miller RA , Hamilton KL , Miller BF (2013) Assessment of mitochondrial biogenesis and mTORC1 signaling during chronic rapamycin feeding in male and female mice. J Gerontol A Biol Sci Med Sci 68: 1493–1501 23657975 10.1093/gerona/glt047 PMC3814233

[msb202211393-bib-0013] Drigo RAE , Lev‐Ram V , Tyagi S , Ramachandra R , Deerinck T , Bushong E , Phan S , Orphan V , Lechene C , Ellisman MH *et al* (2019) Age mosaicism across multiple scales in adult tissues. Cell Metab 30: 343–351.e3 31178361 10.1016/j.cmet.2019.05.010 PMC7289515

[msb202211393-bib-0014] Dunn WA Jr , Cregg JM , Kiel JAKW , van der Klei IJ , Oku M , Sakai Y , Sibirny AA , Stasyk OV , Veenhuis M (2005) Pexophagy: the selective autophagy of peroxisomes. Autophagy 1: 75–83 16874024 10.4161/auto.1.2.1737

[msb202211393-bib-0015] Emont MP , Jacobs C , Essene AL , Pant D , Tenen D , Colleluori G , Vincenzo AD , Jørgensen AM , Dashti H , Stefek A *et al* (2022) A single‐cell atlas of human and mouse white adipose tissue. Nature 603: 926–933 35296864 10.1038/s41586-022-04518-2 PMC9504827

[msb202211393-bib-0016] Fishbain S , Inobe T , Israeli E , Chavali S , Yu H , Kago G , Babu MM , Matouschek A (2015) Sequence composition of disordered regions fine‐tunes protein half‐life. Nat Struct Mol Biol 22: 214–221 25643324 10.1038/nsmb.2958 PMC4351145

[msb202211393-bib-0017] Fornasiero EF , Mandad S , Wildhagen H , Alevra M , Rammner B , Keihani S , Opazo F , Urban I , Ischebeck T , Sakib MS *et al* (2018) Precisely measured protein lifetimes in the mouse brain reveal differences across tissues and subcellular fractions. Nat Commun 9: 71–17 30315172 10.1038/s41467-018-06519-0 PMC6185916

[msb202211393-bib-0018] Guan S , Price JC , Prusiner SB , Ghaemmaghami S , Burlingame AL (2011) A data processing pipeline for mammalian proteome dynamics studies using stable isotope metabolic labeling. Mol Cell Proteomics 10: M111.010728 10.1074/mcp.M111.010728 PMC3237081 21937731

[msb202211393-bib-0019] Guan S , Price JC , Ghaemmaghami S , Prusiner SB , Burlingame AL (2012) Compartment modeling for mammalian protein turnover studies by stable isotope metabolic labeling. Anal Chem 84: 4014–4021 22444387 10.1021/ac203330z PMC3923578

[msb202211393-bib-0020] Hegele A , Kamburov A , Grossmann A , Sourlis C , Wowro S , Weimann M , Will CL , Pena V , Lührmann R , Stelzl U (2012) Dynamic protein‐protein interaction wiring of the human spliceosome. Mol Cell 45: 567–580 22365833 10.1016/j.molcel.2011.12.034

[msb202211393-bib-0021] Hu P , Liu J , Zhao J , Wilkins BJ , Lupino K , Wu H , Pei L (2018) Single‐nucleus transcriptomic survey of cell diversity and functional maturation in postnatal mammalian hearts. Genes Dev 32: 1344–1357 30254108 10.1101/gad.316802.118 PMC6169839

[msb202211393-bib-0022] Jenkins EC , Shah N , Gomez M , Casalena G , Zhao D , Kenny TC , Guariglia SR , Manfredi G , Germain D (2020) Proteasome mapping reveals sexual dimorphism in tissue‐specific sensitivity to protein aggregations. EMBO Rep 21: e48978 32090465 10.15252/embr.201948978 PMC7132179

[msb202211393-bib-0023] Koyuncu S , Loureiro R , Lee HJ , Wagle P , Krueger M , Vilchez D (2021) Rewiring of the ubiquitinated proteome determines ageing in *C. elegans* . Nature 596: 285–290 34321666 10.1038/s41586-021-03781-z PMC8357631

[msb202211393-bib-0024] Krishna S , Drigo RAE , Capitanio JS , Ramachandra R , Ellisman M , Hetzer MW (2021) Identification of long‐lived proteins in the mitochondria reveals increased stability of the electron transport chain. Dev Cell 56: 2952–2965 34715012 10.1016/j.devcel.2021.10.008 PMC8664086

[msb202211393-bib-0025] Kyte J , Doolittle RF (1982) A simple method for displaying the hydropathic character of a protein. J Mol Biol 157: 105–132 7108955 10.1016/0022-2836(82)90515-0

[msb202211393-bib-0026] Lafontan M , Langin D (2009) Lipolysis and lipid mobilization in human adipose tissue. Prog Lipid Res 48: 275–297 19464318 10.1016/j.plipres.2009.05.001

[msb202211393-bib-0027] Lau E , Cao Q , Ng DCM , Bleakley BJ , Dincer TU , Bot BM , Wang D , Liem DA , Lam MPY , Ge J *et al* (2016) A large dataset of protein dynamics in the mammalian heart proteome. Sci Data 3: 160015 26977904 10.1038/sdata.2016.15 PMC4792174

[msb202211393-bib-0028] van der Lee R , Lang B , Kruse K , Gsponer J , de Groot NS , Huynen MA , Matouschek A , Fuxreiter M , Babu MM (2014) Intrinsically disordered segments affect protein half‐life in the cell and during evolution. Cell Rep 8: 1832–1844 25220455 10.1016/j.celrep.2014.07.055 PMC4358326

[msb202211393-bib-0029] Liao Q , Chiu NHL , Shen C , Chen Y , Vouros P (2007) Investigation of enzymatic behavior of Benzonase/alkaline phosphatase in the digestion of oligonucleotides and DNA by ESI‐LC/MS. Anal Chem 79: 1907–1917 17261027 10.1021/ac062249q

[msb202211393-bib-0030] Macallan DC , Fullerton CA , Neese RA , Haddock K , Park SS , Hellerstein MK (1998) Measurement of cell proliferation by labeling of DNA with stable isotope‐labeled glucose: studies *in vitro*, in animals, and in humans. Proc Natl Acad Sci U S A 95: 708–713 9435257 10.1073/pnas.95.2.708 PMC18485

[msb202211393-bib-0031] MacDonald RA (1961) Lifespan of liver cells: autoradiographic study using tritiated thymidine in normal, cirrhotic, and partially hepatectomized rats. Arch Intern Med 107: 335–343 13764742 10.1001/archinte.1961.03620030023003

[msb202211393-bib-0032] Madeira F , Pearce M , Tivey ARN , Basutkar P , Lee J , Edbali O , Madhusoodanan N , Kolesnikov A , Lopez R (2022) Search and sequence analysis tools services from EMBL‐EBI in 2022. Nucleic Acids Res 50: W276–W279 35412617 10.1093/nar/gkac240 PMC9252731

[msb202211393-bib-0033] Malliaras K , Zhang Y , Seinfeld J , Galang G , Tseliou E , Cheng K , Sun B , Aminzadeh M , Marbán E (2013) Cardiomyocyte proliferation and progenitor cell recruitment underlie therapeutic regeneration after myocardial infarction in the adult mouse heart. EMBO Mol Med 5: 191–209 23255322 10.1002/emmm.201201737 PMC3569637

[msb202211393-bib-0034] Mallik S , Kundu S (2018) Topology and oligomerization of mono‐ and oligomeric proteins regulate their half‐lives in the cell. Structure 26: 869–878 29804822 10.1016/j.str.2018.04.015

[msb202211393-bib-0035] Marrero MC , Barrio‐Hernandez I (2021) Toward understanding the biochemical determinants of protein degradation rates. ACS Omega 6: 5091–5100 33681549 10.1021/acsomega.0c05318 PMC7931188

[msb202211393-bib-0036] Marrero MC , van ADJ D , de Ridder D (2017) Sequence‐based analysis of protein degradation rates. Proteins 85: 1593–1601 28547871 10.1002/prot.25323

[msb202211393-bib-0037] Martin‐Perez M , Villén J (2017) Determinants and regulation of protein turnover in yeast. Cell Syst 5: 283–294 28918244 10.1016/j.cels.2017.08.008 PMC5935796

[msb202211393-bib-0038] Matera AG , Wang Z (2014) A day in the life of the spliceosome. Nat Rev Mol Cell Biol 15: 108–121 24452469 10.1038/nrm3742 PMC4060434

[msb202211393-bib-0039] Mathieson T , Franken H , Kosinski J , Kurzawa N , Zinn N , Sweetman G , Poeckel D , Ratnu VS , Schramm M , Becher I *et al* (2018) Systematic analysis of protein turnover in primary cells. Nat Commun 9: 1–10 29449567 10.1038/s41467-018-03106-1 PMC5814408

[msb202211393-bib-0040] Matsuda M , Hayashi H , Garcia‐Ojalvo J , Yoshioka‐Kobayashi K , Kageyama R , Yamanaka Y , Ikeya M , Toguchida J , Alev C , Ebisuya M (2020) Species‐specific segmentation clock periods are due to differential biochemical reaction speeds. Science 369: 1450–1455 32943519 10.1126/science.aba7668

[msb202211393-bib-0041] McAlister GC , Nusinow DP , Jedrychowski MP , Wühr M , Huttlin EL , Erickson BK , Rad R , Haas W , Gygi SP (2014) MultiNotch MS3 enables accurate, sensitive, and multiplexed detection of differential expression across cancer cell line proteomes. Anal Chem 86: 7150–7158 24927332 10.1021/ac502040v PMC4215866

[msb202211393-bib-0042] McClatchy DB , Dong M‐Q , Wu CC , Venable JD , Yates JR (2007) 15N metabolic labeling of mammalian tissue with slow protein turnover. J Proteome Res 6: 2005–2010 17375949 10.1021/pr060599n PMC2527585

[msb202211393-bib-0043] McShane E , Sin C , Zauber H , Wells JN , Donnelly N , Wang X , Hou J , Chen W , Storchova Z , Marsh JA *et al* (2016) Kinetic analysis of protein stability reveals age‐dependent degradation. Cell 167: 803–815 27720452 10.1016/j.cell.2016.09.015

[msb202211393-bib-0044] McWilliams TG , Prescott AR , Allen GFG , Tamjar J , Munson MJ , Thomson C , Muqit MMK , Ganley IG (2016) Mito‐QC illuminates mitophagy and mitochondrial architecture *in vivo* . J Cell Biol 214: 333–345 27458135 10.1083/jcb.201603039 PMC4970326

[msb202211393-bib-0045] Meldal BHM , Bye‐A‐Jee H , Gajdoš L , Hammerová Z , Horáčková A , Melicher F , Perfetto L , Pokorný D , Lopez MR , Türková A *et al* (2018) Complex portal 2018: extended content and enhanced visualization tools for macromolecular complexes. Nucleic Acids Res 47: gky1001 10.1093/nar/gky1001 PMC6323931 30357405

[msb202211393-bib-0046] Mészáros B , Erdős G , Dosztányi Z (2018) IUPred2A: context‐dependent prediction of protein disorder as a function of redox state and protein binding. Nucleic Acids Res 46: W329–W337 29860432 10.1093/nar/gky384 PMC6030935

[msb202211393-bib-0047] Miller BF , Reid JJ , Price JC , Lin H‐JL , Atherton PJ , Smith K (2020) CORP: the use of deuterated water for the measurement of protein synthesis. J Appl Physiol 128: 1163–1176 32213116 10.1152/japplphysiol.00855.2019

[msb202211393-bib-0048] Mizushima N , Yamamoto A , Matsui M , Yoshimori T , Ohsumi Y (2004) *In vivo* analysis of autophagy in response to nutrient starvation using transgenic mice expressing a fluorescent autophagosome marker. Mol Biol Cell 15: 1101–1111 14699058 10.1091/mbc.E03-09-0704 PMC363084

[msb202211393-bib-0049] Monastyrska I , Klionsky DJ (2006) Autophagy in organelle homeostasis: peroxisome turnover. Mol Aspects Med 27: 483–494 16973210 10.1016/j.mam.2006.08.004 PMC1993912

[msb202211393-bib-0050] Neese RA , Misell LM , Turner S , Chu A , Kim J , Cesar D , Hoh R , Antelo F , Strawford A , McCune JM *et al* (2002) Measurement *in vivo* of proliferation rates of slow turnover cells by 2H2O labeling of the deoxyribose moiety of DNA. Proc Natl Acad Sci U S A 99: 15345–15350 12424339 10.1073/pnas.232551499 PMC137719

[msb202211393-bib-0051] Neff NF , May AP , Wyss‐Coray T , Batson J , Botvinnik O , Chen MB , Chen S , Green F , Jones RC , Maynard A *et al* (2018) Single‐cell transcriptomics of 20 mouse organs creates a Tabula Muris. Nature 562: 367–372 30283141 10.1038/s41586-018-0590-4 PMC6642641

[msb202211393-bib-0052] Pascual F , Coleman RA (2016) Fuel availability and fate in cardiac metabolism: a tale of two substrates. Biochim Biophys Acta 1861: 1425–1433 26993579 10.1016/j.bbalip.2016.03.014 PMC4983230

[msb202211393-bib-0053] Price JC , Guan S , Burlingame A , Prusiner SB , Ghaemmaghami S (2010) Analysis of proteome dynamics in the mouse brain. Proc Natl Acad Sci U S A 107: 14508–14513 20699386 10.1073/pnas.1006551107 PMC2922600

[msb202211393-bib-0054] Quaglia F , Mészáros B , Salladini E , Hatos A , Pancsa R , Chemes LB , Pajkos M , Lazar T , Peña‐Díaz S , Santos J *et al* (2021) DisProt in 2022: improved quality and accessibility of protein intrinsic disorder annotation. Nucleic Acids Res 50: D480–D487 10.1093/nar/gkab1082 PMC8728214 34850135

[msb202211393-bib-0055] Quinlivan EP , Gregory JF 3rd (2008) DNA digestion to deoxyribonucleoside: a simplified one‐step procedure. Anal Biochem 373: 383–385 18028864 10.1016/j.ab.2007.09.031 PMC2239294

[msb202211393-bib-0056] Rath S , Sharma R , Gupta R , Ast T , Chan C , Durham TJ , Goodman RP , Grabarek Z , Haas ME , Hung WHW *et al* (2020) MitoCarta3.0: an updated mitochondrial proteome now with sub‐organelle localization and pathway annotations. Nucleic Acids Res 49: gkaa1011 10.1093/nar/gkaa1011 PMC7778944 33174596

[msb202211393-bib-0057] Raudvere U , Kolberg L , Kuzmin I , Arak T , Adler P , Peterson H , Vilo J (2019) G:profiler: a web server for functional enrichment analysis and conversions of gene lists (2019 update). Nucleic Acids Res 47: W191–W198 31066453 10.1093/nar/gkz369 PMC6602461

[msb202211393-bib-0058] Reichard P (1988) Interactions between deoxyribonucleotide and DNA synthesis. Annu Rev Biochem 57: 349–374 3052277 10.1146/annurev.bi.57.070188.002025

[msb202211393-bib-0059] Reome JB , Johnston DS , Helmich BK , Morgan TM , Dutton‐Swain N , Dutton RW (2000) The effects of prolonged administration of 5‐bromodeoxyuridine on cells of the immune system. J Immunol 165: 4226–4230 11035055 10.4049/jimmunol.165.8.4226

[msb202211393-bib-0060] Richter ML , Deligiannis IK , Yin K , Danese A , Lleshi E , Coupland P , Vallejos CA , Matchett KP , Henderson NC , Colome‐Tatche M *et al* (2021) Single‐nucleus RNA‐seq2 reveals functional crosstalk between liver zonation and ploidy. Nat Commun 12: 4264 34253736 10.1038/s41467-021-24543-5 PMC8275628

[msb202211393-bib-0061] Rigamonti A , Brennand K , Lau F , Cowan CA (2011) Rapid cellular turnover in adipose tissue. PLoS One 6: e17637 21407813 10.1371/journal.pone.0017637 PMC3047582

[msb202211393-bib-0062] Rolfs Z , Frey BL , Shi X , Kawai Y , Smith LM , Welham NV (2021) An atlas of protein turnover rates in mouse tissues. Nat Commun 12: 6778 34836951 10.1038/s41467-021-26842-3 PMC8626426

[msb202211393-bib-0063] Ross AB , Langer JD , Jovanovic M (2021) Proteome turnover in the spotlight: approaches, applications, and perspectives. Mol Cell Proteomics 20: 100016 33556866 10.1074/mcp.R120.002190 PMC7950106

[msb202211393-bib-0064] Savas JN , Toyama BH , Xu T , Yates JR 3rd , Hetzer MW (2012) Extremely long‐lived nuclear pore proteins in the rat brain. Science 335: 942 22300851 10.1126/science.1217421 PMC3296478

[msb202211393-bib-0065] Sender R , Milo R (2021) The distribution of cellular turnover in the human body. Nat Med 27: 45–48 33432173 10.1038/s41591-020-01182-9

[msb202211393-bib-0066] Shi Z , Fujii K , Kovary KM , Genuth NR , Röst HL , Teruel MN , Barna M (2017) Heterogeneous ribosomes preferentially translate distinct subpools of mRNAs genome‐wide. Mol Cell 67: 71–83 28625553 10.1016/j.molcel.2017.05.021 PMC5548184

[msb202211393-bib-0067] Singh R , Kaushik S , Wang Y , Xiang Y , Novak I , Komatsu M , Tanaka K , Cuervo AM , Czaja MJ (2009) Autophagy regulates lipid metabolism. Nature 458: 1131–1135 19339967 10.1038/nature07976 PMC2676208

[msb202211393-bib-0068] Su D , Chan CTY , Gu C , Lim KS , Chionh YH , McBee ME , Russell BS , Babu IR , Begley TJ , Dedon PC (2014) Quantitative analysis of ribonucleoside modifications in tRNA by HPLC‐coupled mass spectrometry. Nat Protoc 9: 828–841 24625781 10.1038/nprot.2014.047 PMC4313537

[msb202211393-bib-0069] Supek F , Bošnjak M , Škunca N , Šmuc T (2011) REVIGO summarizes and visualizes long lists of gene ontology terms. PLoS One 6: e21800 21789182 10.1371/journal.pone.0021800 PMC3138752

[msb202211393-bib-0070] Swovick K , Welle KA , Hryhorenko JR , Seluanov A , Gorbunova V , Ghaemmaghami S (2020) Cross‐species comparison of proteome turnover kinetics*. Mol Cell Proteomics 17: 580–591 10.1074/mcp.RA117.000574 PMC5880112 29321186

[msb202211393-bib-0071] Taylor A , Davies KJA (1987) Protein oxidation and loss of protease activity may lead to cataract formation in the aged lens. Free Radic Biol Med 3: 371–377 3322949 10.1016/0891-5849(87)90015-3

[msb202211393-bib-0072] Taylor RC , Dillin A (2011) Aging as an event of proteostasis collapse. Cold Spring Harb Perspect Biol 3: a004440 21441594 10.1101/cshperspect.a004440 PMC3101847

[msb202211393-bib-0073] Thompson ACS , Bruss MD , Price JC , Khambatta CF , Holmes WE , Colangelo M , Dalidd M , Roberts LS , Astle CM , Harrison DE *et al* (2016) Reduced *in vivo* hepatic proteome replacement rates but not cell proliferation rates predict maximum lifespan extension in mice. Aging Cell 15: 118–127 26541492 10.1111/acel.12414 PMC4717272

[msb202211393-bib-0074] Toyama BH , Savas JN , Park SK , Harris MS , Ingolia NT , Yates JR 3rd , Hetzer MW (2013) Identification of long‐lived proteins reveals exceptional stability of essential cellular structures. Cell 154: 971–982 23993091 10.1016/j.cell.2013.07.037 PMC3788602

[msb202211393-bib-0075] Toyama BH , Drigo RAE , Lev‐Ram V , Ramachandra R , Deerinck TJ , Lechene C , Ellisman MH , Hetzer MW (2019) Visualization of long‐lived proteins reveals age mosaicism within nuclei of postmitotic cells. J Cell Biol 218: 433–444 30552100 10.1083/jcb.201809123 PMC6363465

[msb202211393-bib-0076] Vonk WIM , Rainbolt TK , Dolan PT , Webb AE , Brunet A , Frydman J (2020) Differentiation drives widespread rewiring of the neural stem cell chaperone network. Mol Cell 78: 329–345 32268122 10.1016/j.molcel.2020.03.009 PMC7288733

[msb202211393-bib-0077] Welle KA , Zhang T , Hryhorenko JR , Shen S , Qu J , Ghaemmaghami S (2016) Time‐resolved analysis of proteome dynamics by tandem mass tags and stable isotope labeling in cell culture (TMT‐SILAC) Hyperplexing. Mol Cell Proteomics 15: 3551–3563 27765818 10.1074/mcp.M116.063230 PMC5141271

[msb202211393-bib-0078] Winter D , Becker T (2021) Surveying the mitochondrial proteome. Nat Cell Biol 23: 1216–1217 34873282 10.1038/s41556-021-00801-y

[msb202211393-bib-0079] Wu RS , Tsai S , Bonner WM (1982) Patterns of histone variant synthesis can distinguish go from G1 cells. Cell 31: 367–374 7159927 10.1016/0092-8674(82)90130-1

[msb202211393-bib-0080] Wu C , Ba Q , Lu D , Li W , Salovska B , Hou P , Mueller T , Rosenberger G , Gao E , Di Y *et al* (2021) Global and site‐specific effect of phosphorylation on protein turnover. Dev Cell 56: 111–124 33238149 10.1016/j.devcel.2020.10.025 PMC7855865

[msb202211393-bib-0081] Youle RJ , Narendra DP (2011) Mechanisms of mitophagy. Nat Rev Mol Cell Biol 12: 9–14 21179058 10.1038/nrm3028 PMC4780047

[msb202211393-bib-0082] Zecha J , Gabriel W , Spallek R , Chang Y‐C , Mergner J , Wilhelm M , Bassermann F , Kuster B (2022) Linking post‐translational modifications and protein turnover by site‐resolved protein turnover profiling. Nat Commun 13: 165 35013197 10.1038/s41467-021-27639-0 PMC8748498

[msb202211393-bib-0083] Zechner R , Madeo F , Kratky D (2017) Cytosolic lipolysis and lipophagy: two sides of the same coin. Nat Rev Mol Cell Biol 18: 671–684 28852221 10.1038/nrm.2017.76

