# Supplementary material for: Turnover and replication analysis by isotope labeling (TRAIL) reveals the influence of tissue context on protein and organelle lifetimes
Source: Mol Syst Biol. 2023 Mar 17;19(4):e11393. doi: 10.15252/msb.202211393 (PMC10090950; doi:10.15252/msb.202211393)
Supplement: Supplementary file 1 — Appendix S1 [file MSB-19-e11393-s001.pdf]

Appendix to Hasper et al., 2023, containing 15 Supplementary Figures and legends:

Appendix Fig. S1	page 1
Appendix Fig. S2	page 2
Appendix Fig. S3	page 3
Appendix Fig. S4	page 5
Appendix Fig. S5	page 7
Appendix Fig. S6	page 8
Appendix Fig. S7	page 9
Appendix Fig. S8	page 10
Appendix Fig. S9	page 11
Appendix Fig. S10	page 12
Appendix Fig. S11	page 13
Appendix Fig. S12	page 14
Appendix Fig. S13	page 15
Appendix Fig. S14	page 16
Appendix Fig. S15	page 17
Appendix Fig. S16	page 18
Appendix Fig. S17	page 19

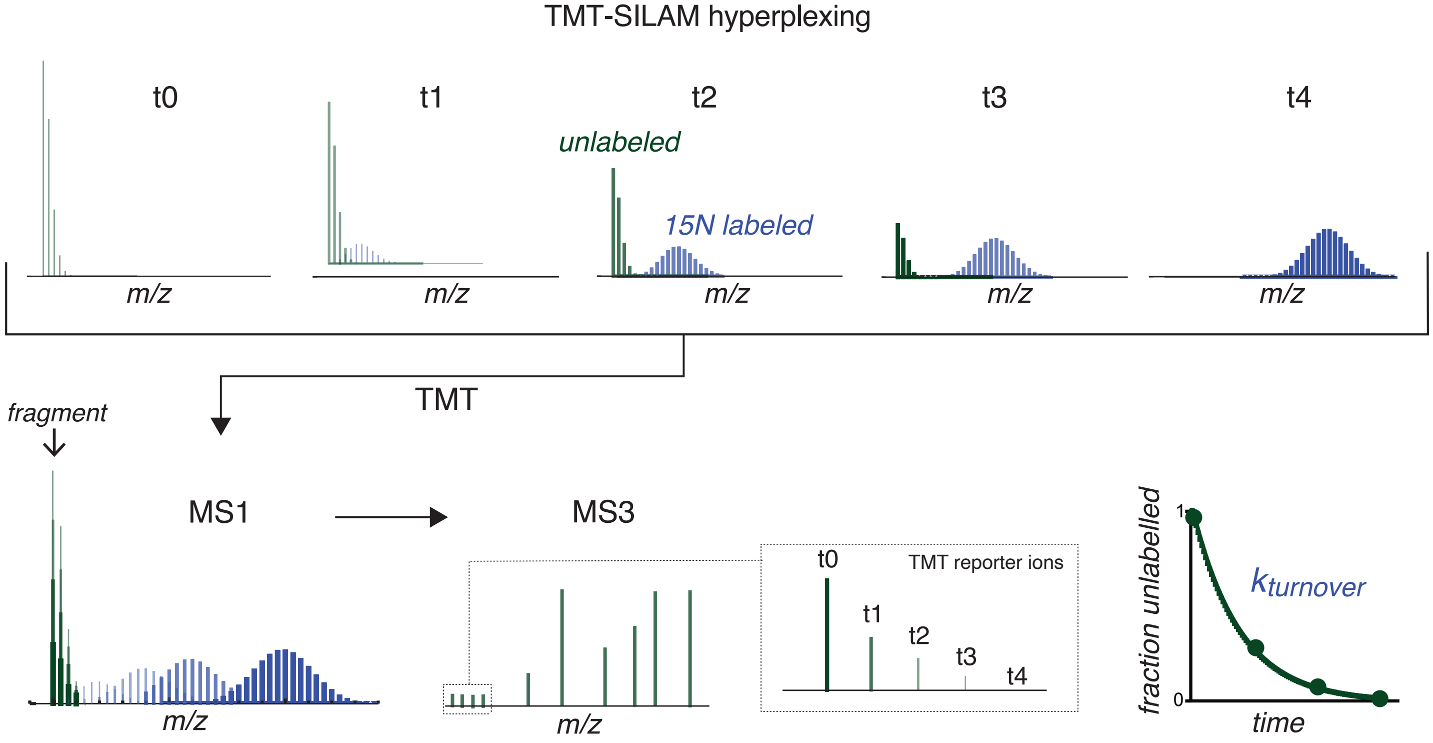

Appendix Fig. S1. Schematic of TMT-SILAM hyperplexed determination of kt by tracking the rate of loss of unlabeled peptides over time. Samples from multiple timepoints are hyperplexed by conjugation with unique TMT tags and combined into a single sample before LC-MS/MS. A peptide detected in multiple time points will have a single m/z in MS1, but will fragment into reporter ions with distinct masses in MS2/MS3. Quantification of SILAM-unlabeled MS3 peaks reveals the kinetics of loss of the unlabeled peptides, which can be fit to determine kt.

Appendix Figure S2

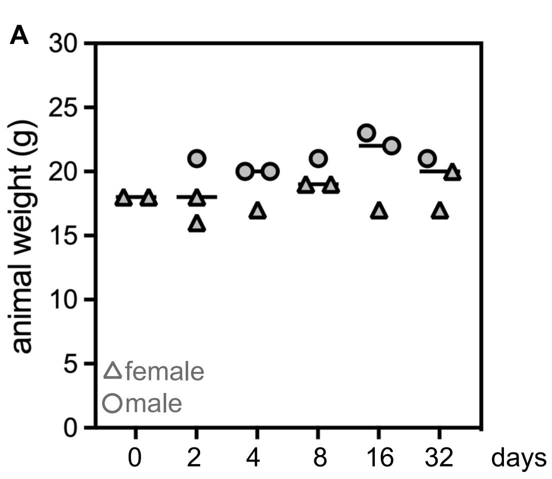

(A) Weights of animals on date of sacrifice for TRAIL timecourse. (B) Weight of 3 animals over 32 days of labeling.

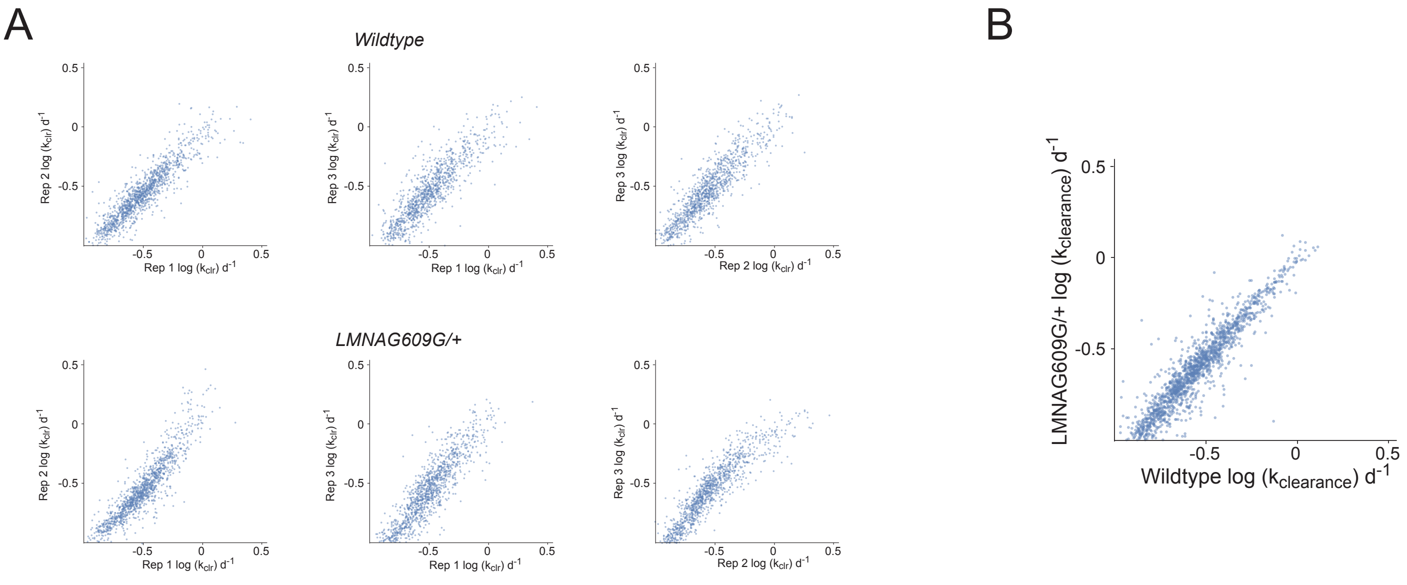

C

	proteins detected	proteins quantified	median PSMs quantified per protein	median k_{clr} (d-1)	median k_{clr} std. error(d-1)
liver	3,390	1,577	21.5	0.282	0.016
heart	2,652	1,187	24.6	0.106	0.007
fat	2,875	1,104	23.0	0.115	0.010
intestine	4,329	2,019	22.5	0.395	0.026

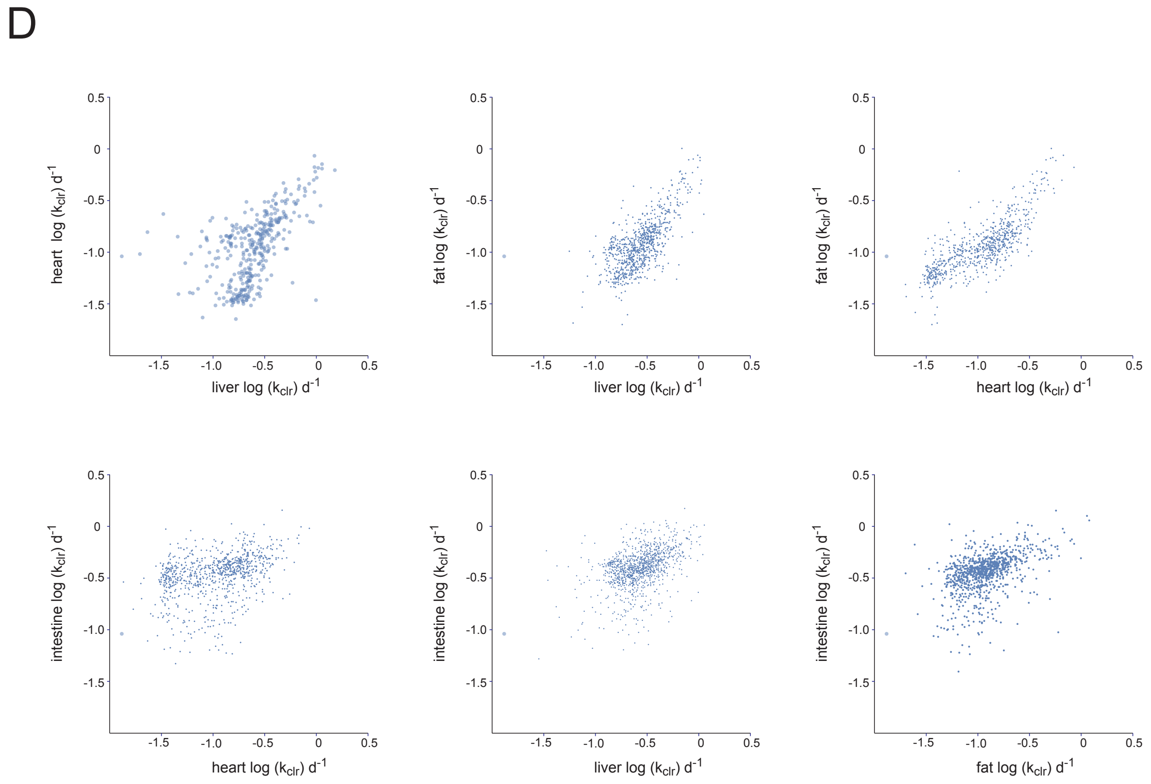

Appendix Fig. S3. (A) Representative scatterplots showing high concordance of k_t values determined across 3 replicate experiments each in wild type (top) and progeroid (*LMNA*$^{G609G/+}$, bottom) liver. (B) Scatterplot showing correlation in k_t values between wild type and progeroid liver. (C) Overview of proteins detected and quantified in each tissue. (D) Pairwise correlation of k_t values for proteins detected in liver, heart, fat, and intestine.

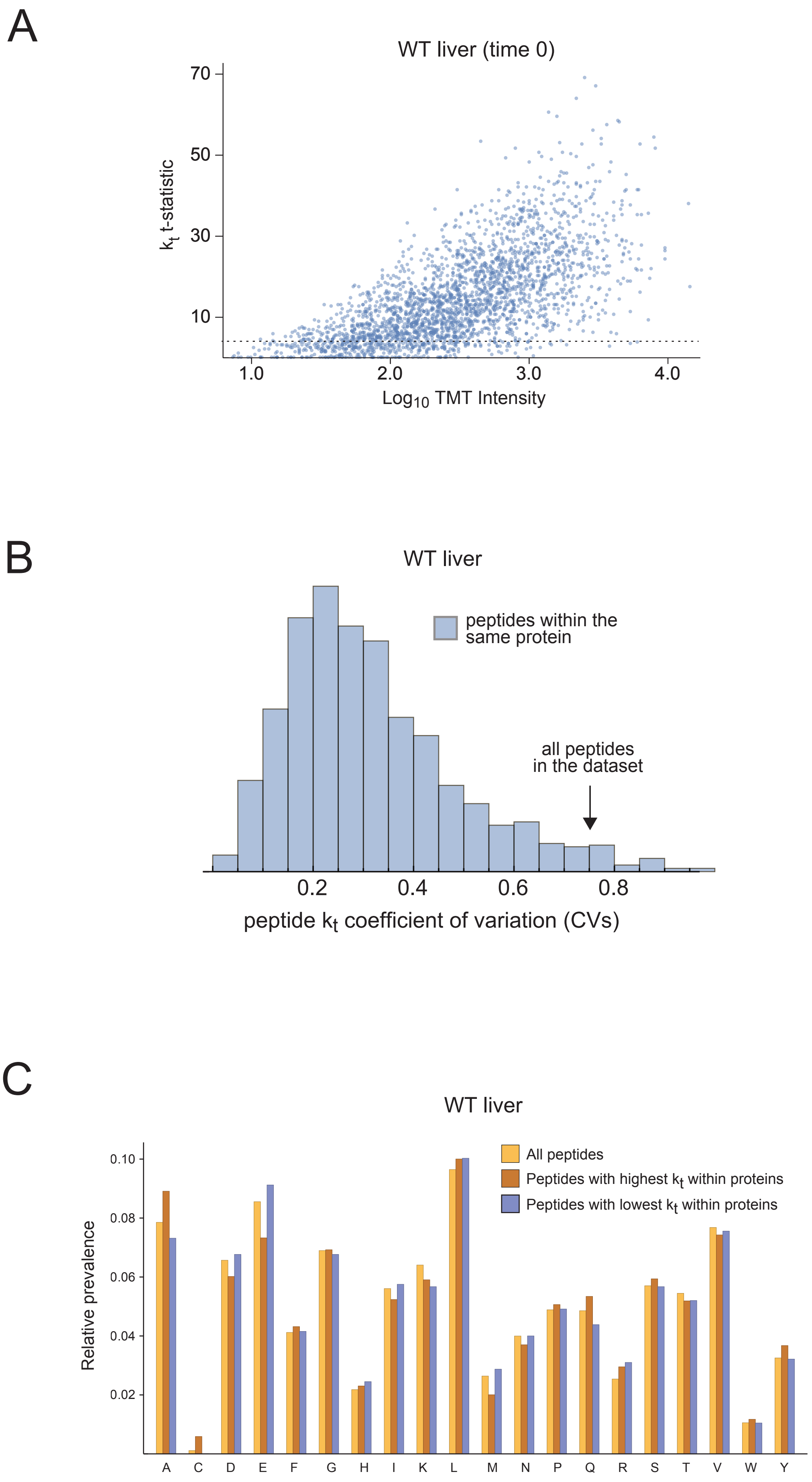
A
WT liver (time 0)
k_t t-statistic
70
50
30
10
1.0
2.0
3.0
4.0
Log_{10} TMT Intensity
B
WT liver
peptides within the same protein
all peptides in the dataset
0.2
0.4
0.6
0.8
peptide k_t coefficient of variation (CVs)
C
WT liver
All peptides
Peptides with highest k_t within proteins
Peptides with lowest k_t within proteins
Relative prevalence
0.10
0.08
0.06
0.04
0.02
A C D E F G H I K L M N P Q R S T V W Y

Appendix Figure S4. (A) Higher intensity reporter ions lead to rate measurements with lower relative errors. As an example, the plot shows that the intensities of TMT reporter ions for WT liver (t=0) measurements are positively correlated with the t-statistics of k_t measurements. A similar trend is observed for all of our rate measurements. Thus, as expected, higher intensity ions result in more precise k_t measurements However, as described in the text, we use strict criteria (>3 PSMs, 2 or more replicate measurements and t-stat>3 as shown by the dotted line in the plot) to filter our measurements prior to downstream analyses. (B) The variabilities of k_t measurements for peptides mapped to the same protein are significantly lower than the peptides in the dataset at large. This can be shown by the fact that the distribution of coefficient of variations (CVs) of peptides within the same proteins is significantly lower than all peptides in our dataset. The figure demonstrates this for WT liver data and the same trend holds for all tissues. (C) Amino acid composition of peptides does not significantly influence the magnitude of k_t measurements. To demonstrate this, we analyzed the prevalence of amino acids within the fastest and slowest labeling peptides within the proteins in our dataset. As can be seen in the plot for the WT liver data, there is no significant enrichment for specific amino acids within fast or slow labeling peptides. The same trend holds for other tissues.

Appendix Figure S5.

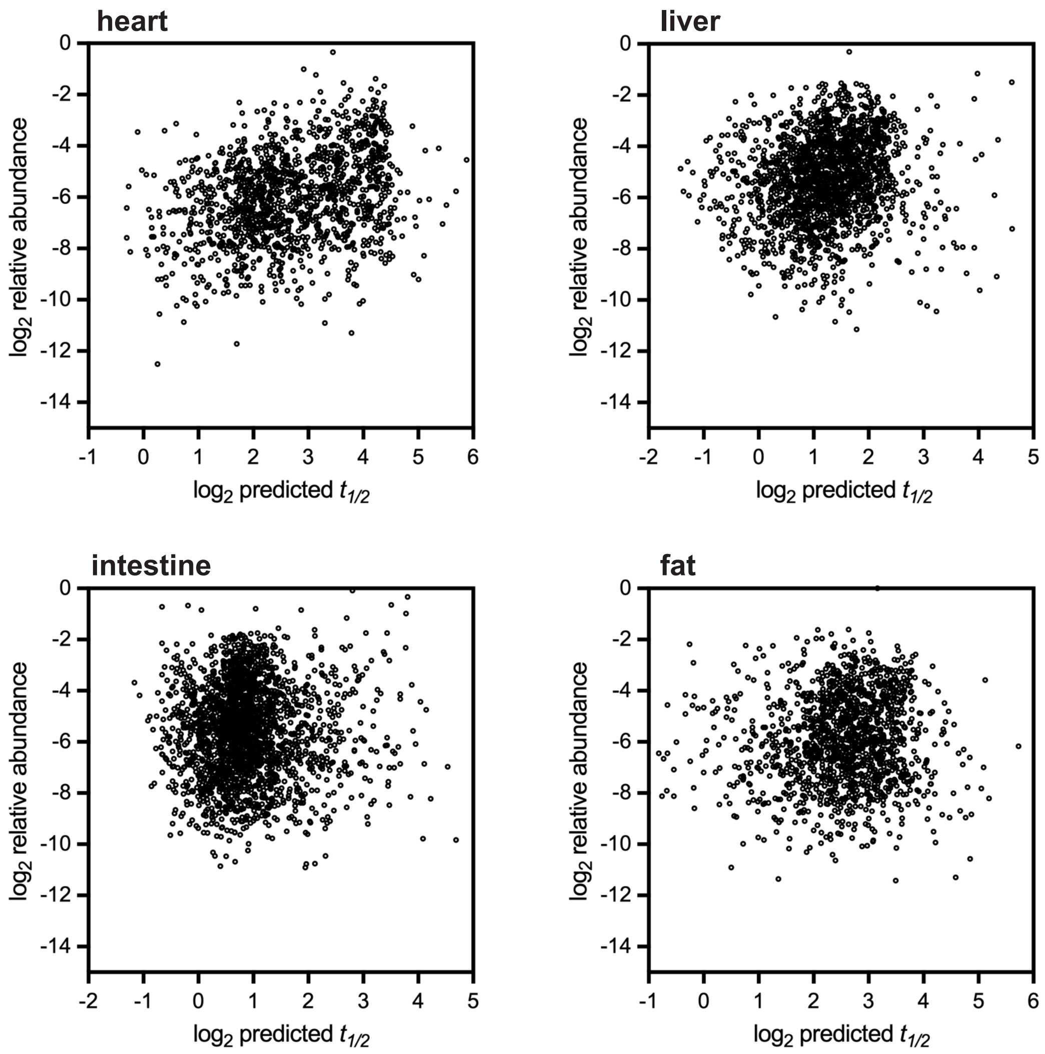

Analysis of correlation between relative protein abundance and protein half-life in wild-type heart, liver, intestine and fat.

Appendix Figure S6.

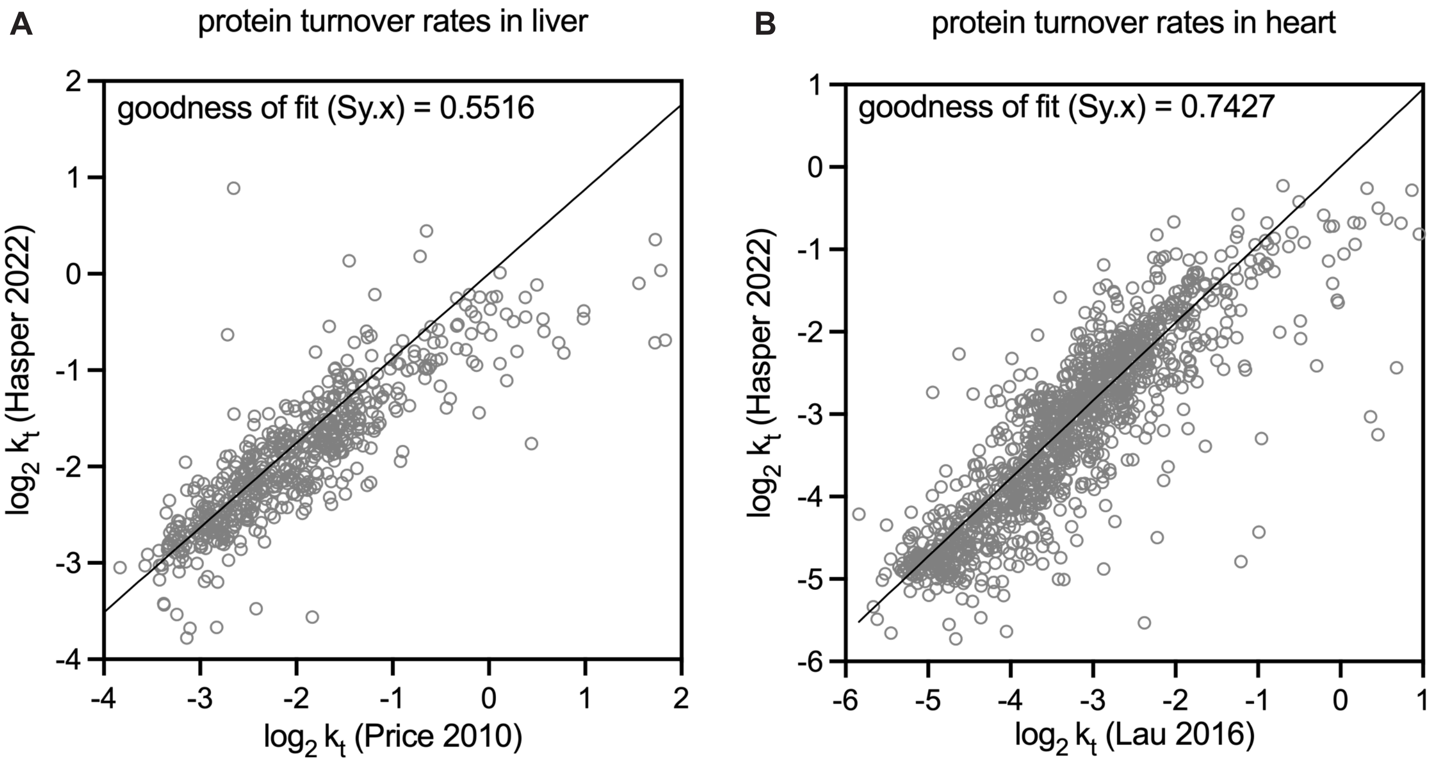

Comparison of protein turnover rates determined in this study to previously published protein turnover data determined by ^{15}N labeling of the mouse liver (A, Price et al., *PNAS* 2010) and by D_2O labeling of the mouse heart (B, Lau et al., *Sci Data* 2016).

Appendix Figure S7.

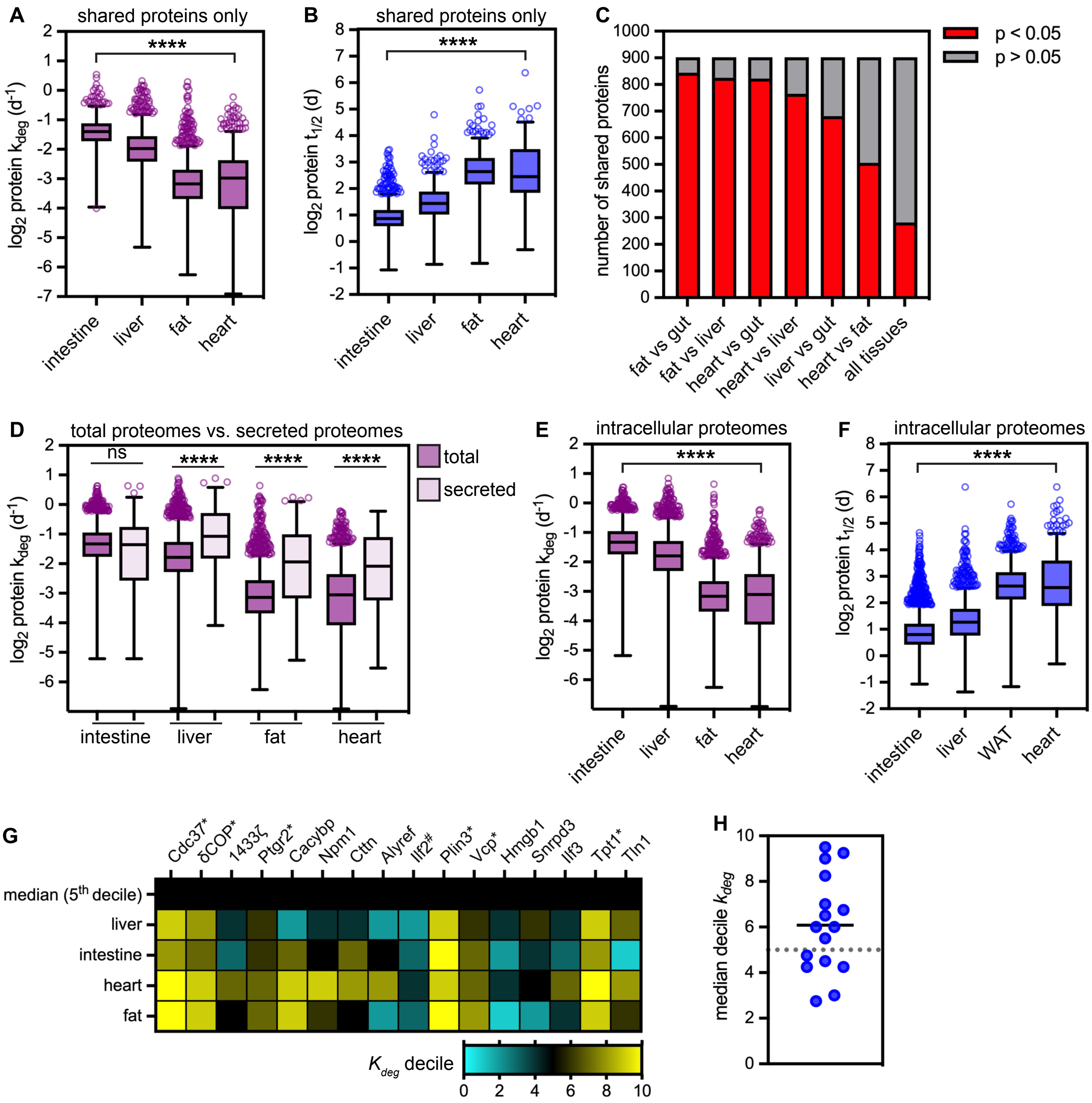

(A) Degradation rates (k_t) and (B) predicted half-lives ($t_{1/2}$) determined by SILAM-TMT of 902 proteins detected across intestine, liver, fat, and heart. **** indicates that all medians are significantly different ($p < 0.0001$, Kruskal -Wallis test). Median $t_{1/2}$ for the shared proteomes of these four tissues are 1.8 days (intestine), 2.7 days (liver), 6.3 days (fat), and 5.5 days (heart). (C) Proportion of proteins whose values of k_t are significantly different across the indicated pairwise comparisons. Significance determined by t-test. (D) Comparison of turnover rates of secreted proteins (149 in intestine; 103 in liver; 99 in fat; and 96 in heart) versus total tissue proteomes (data reproduced from Figure 3A). **** indicates that distributions are significantly different in the heart, liver, and fat ($p <0.0001$, Mann-Whitney test), but not in the intestine. (E) k_t and (F) predicted $t_{1/2}$ for intracellular proteomes. **** indicates that all medians are significantly different ($p <0.0001$, Kruskal-Wallis test). Median half-lives for the intracellular proteomes of these four tissues are 1.7 days (intestine, n = 2570), 2.4 days (liver, n = 1996), 6.2 days (fat, n = 1511), and 6 days (heart, n = 1539). (G) Heatmap of k_t deciles for 16 intrinsically disordered proteins (IDPs). *indicates proteins whose k_t rates are significantly faster than the proteome median across all tissues; #indicates proteins whose k_t rates are significantly slower than the proteome median across all tissues. IDP annotations from the DisProt database. (H) Median decile k_t value for 16 IDPs across 4 tissues is not significantly different from the k_t median (5th decile).

Appendix Fig. S8.

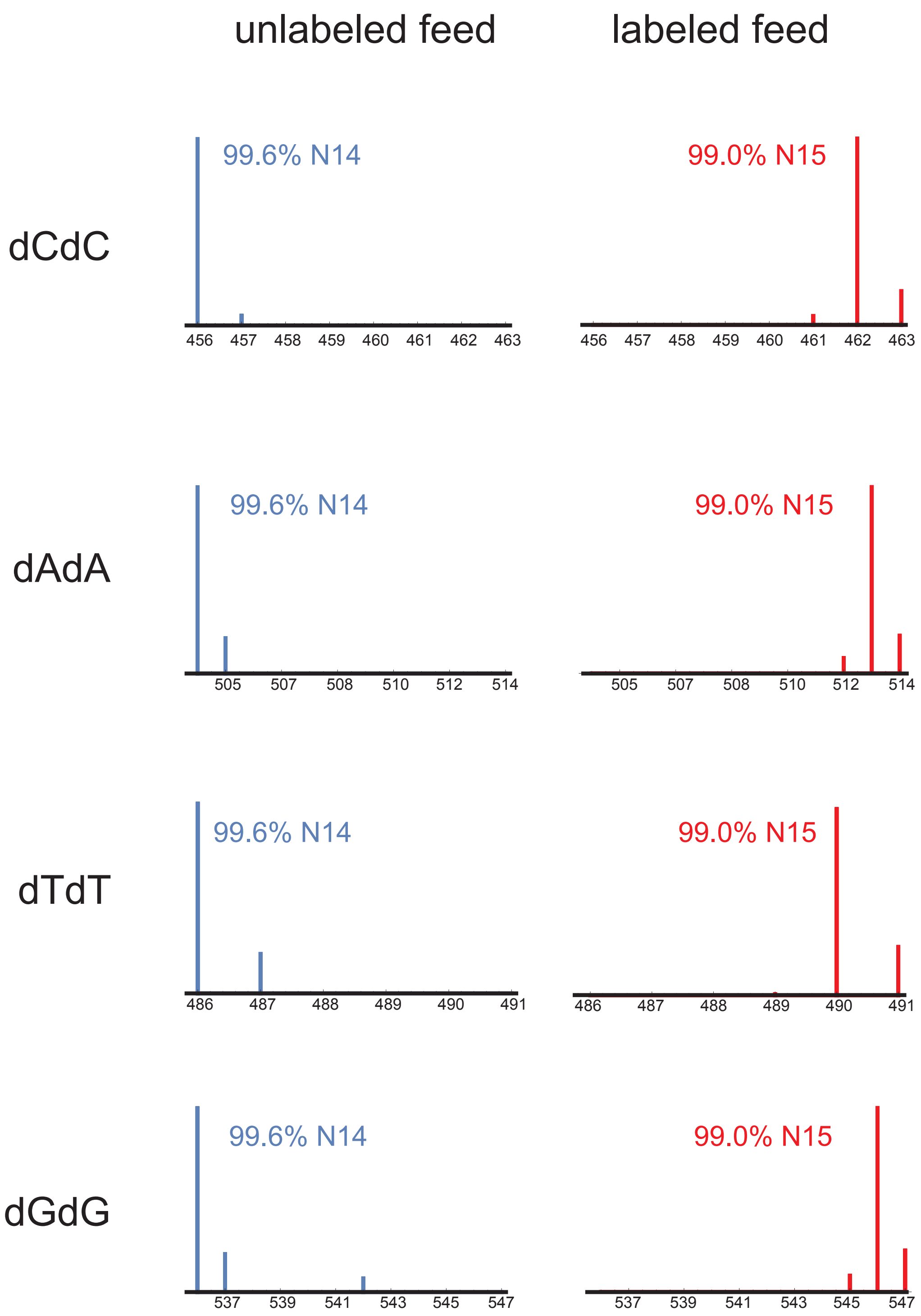

Analysis of ^{15}N vs. ^{14}N isotope abundance in dinucleotides isolated from unlabeled chow versus ^{15}N-labeled SILAM chow.

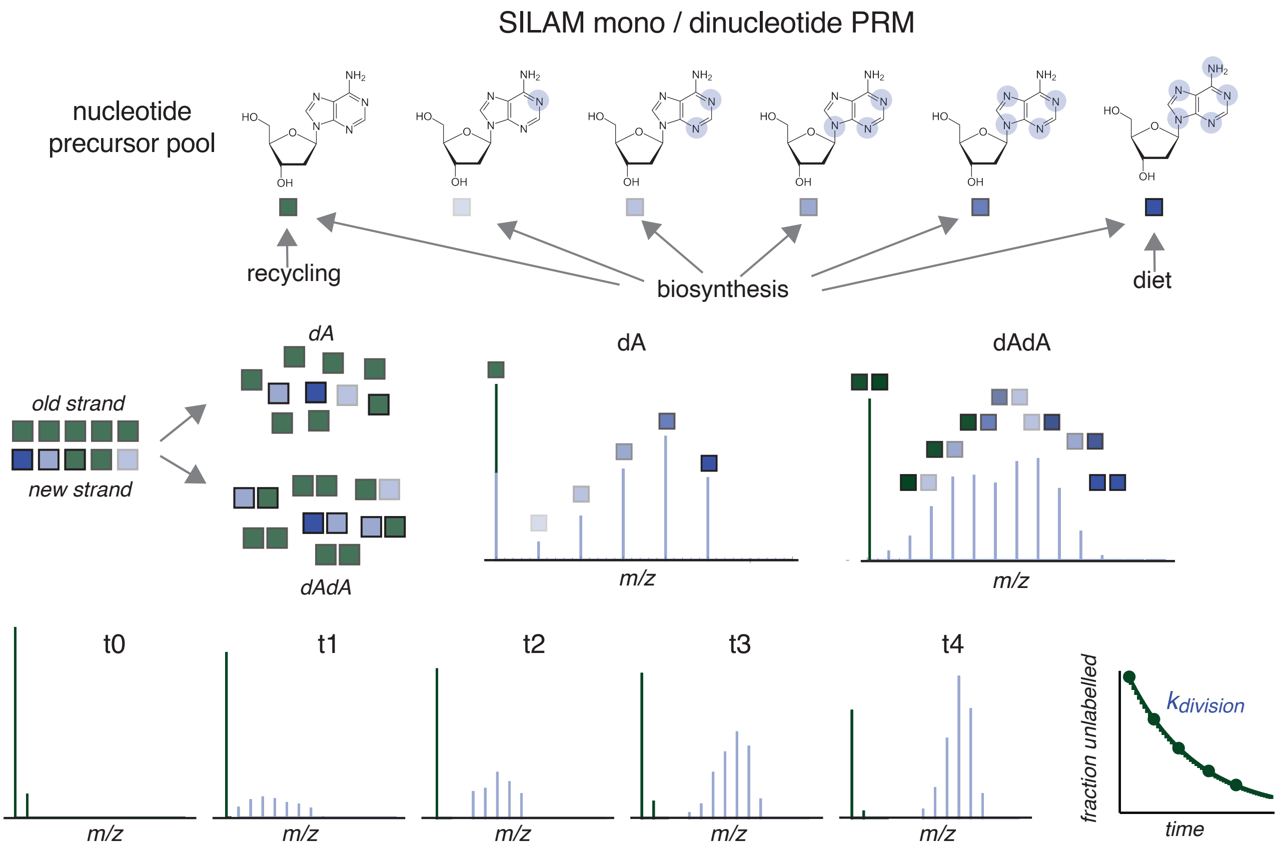

Appendix Fig. S9. Schematic of SILAM mono and dinucleotide PRM. The relative isotope abundance (RIA) of the nucleotide precursor pool reflects the rates of precursor uptake, biosynthesis, and recycling of pre-existing nucleotides. By quantifying the abundances of unlabeled, partially labeled, and fully labeled di-nucleotides in genomic DNA, the extent of unlabeled nucleotide recycling can be inferred and can be used to determine cell division rates (kdiv).

Appendix Figure S10.

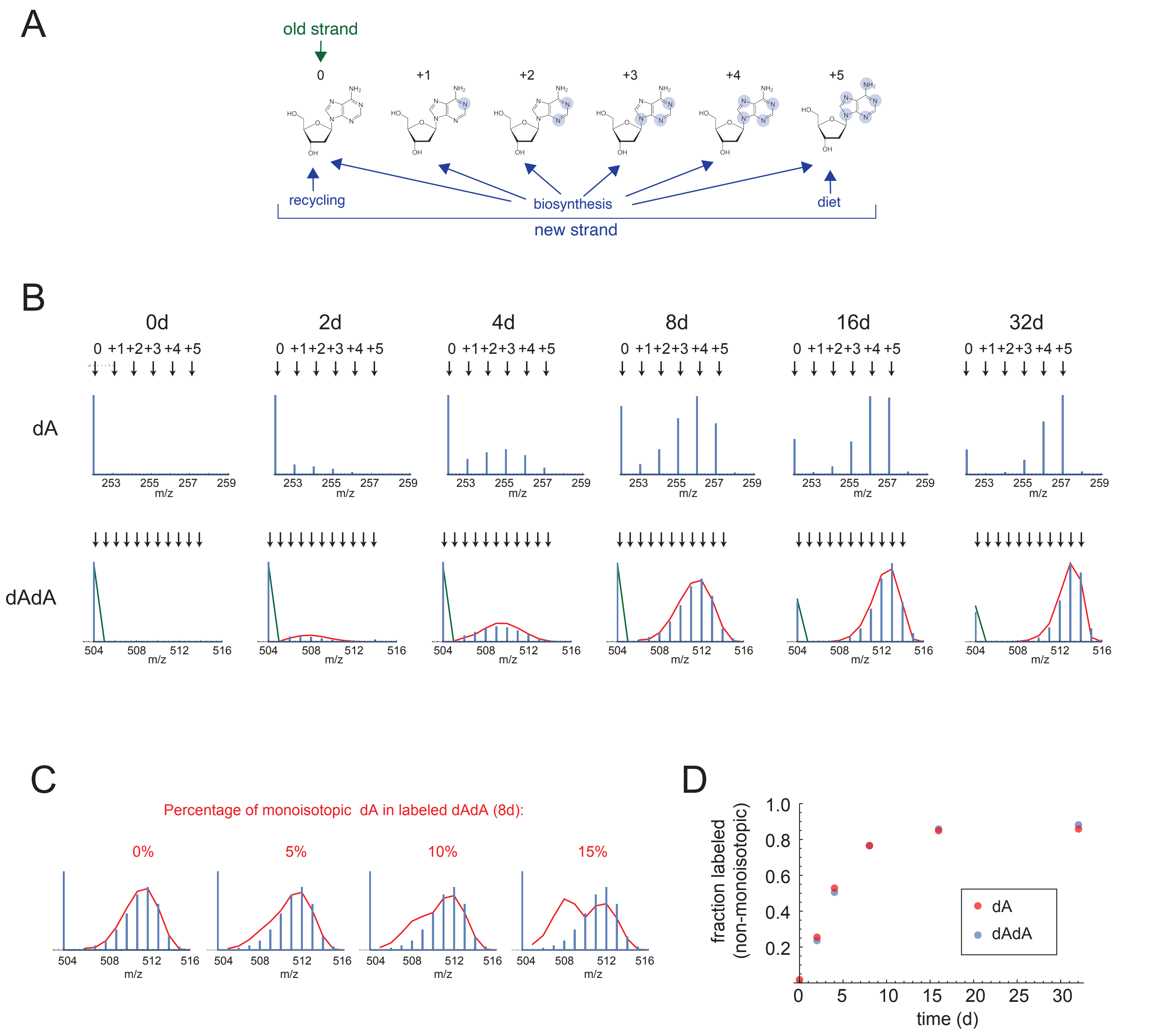

(A) Diagram of sources of nucleotides that may be used in vivo for genomic DNA synthesis during a SILAM experiment. (B) Example labeling pattern for deoxyadenosine (dA, top) and dAdA dinucleotides (bottom) over 32 days of labeling in mouse intestine; note that the unlabeled peak is monoisotopic while the labeled peaks gradually increase in *m/z* over time. Red line indicates the distribution of labeled peaks that would be expected if the precursor pool were composed entirely of labeled species derived from either biosynthesis or diet as diagrammed in (A), with no contributioon from the unlabeled pool. (C) Fitting the observed distribution of dAdA by assuming 0-15% the source nucleotide comes from recycled unlabeled dA. Note that the best curve fit is obtained for the 0% recycling condition. (D) Close agreement of label incorporatioon observed between mononucleosides (dA) and dinucleotides (dAdA) over 32 days of labeling in wild type intestine.

Appendix Fig. S11

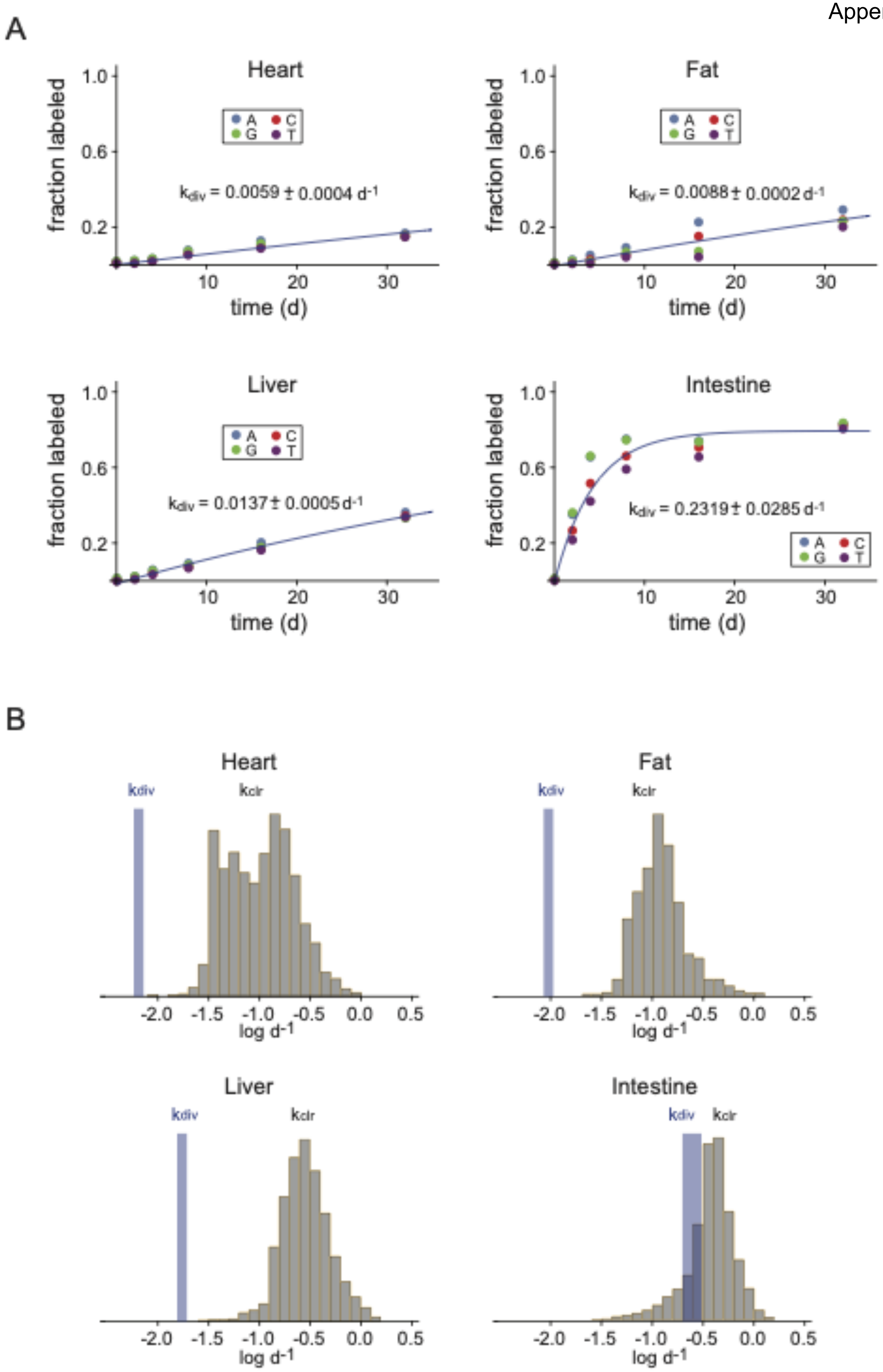

(A) Curve fits for mononucleosides isolated from genomic DNA in heart, fat, liver, and intestine. (B) Comparison of proteome k_t values to cell k_{div} values in each tissue.

Appendix Fig. S12

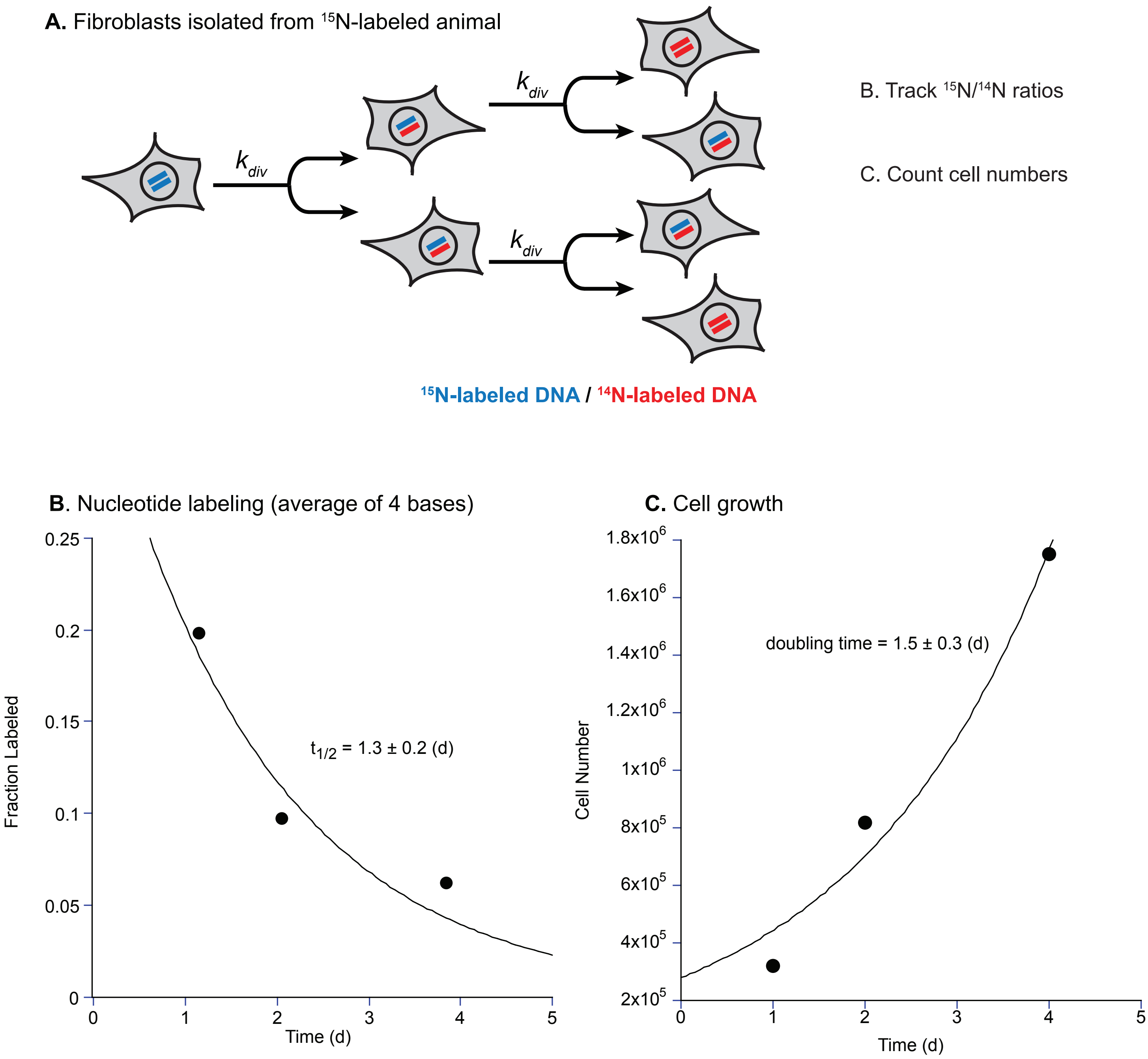

(A) Schematic of cell turnover benchmarking experiment. Primary fibroblasts were isolated from the ear of a mouse labeled with 15N for 256 days. Cells were subcultured, and genomic DNA was extracted at 3 timepoints to track cell division rates by 15N/14N labeling ratios (B). At each timepoint, cell numbers were also quantified (C). Both datasets were fit to determine cell division rates and cell doubling times.

Appendix Figure S13.

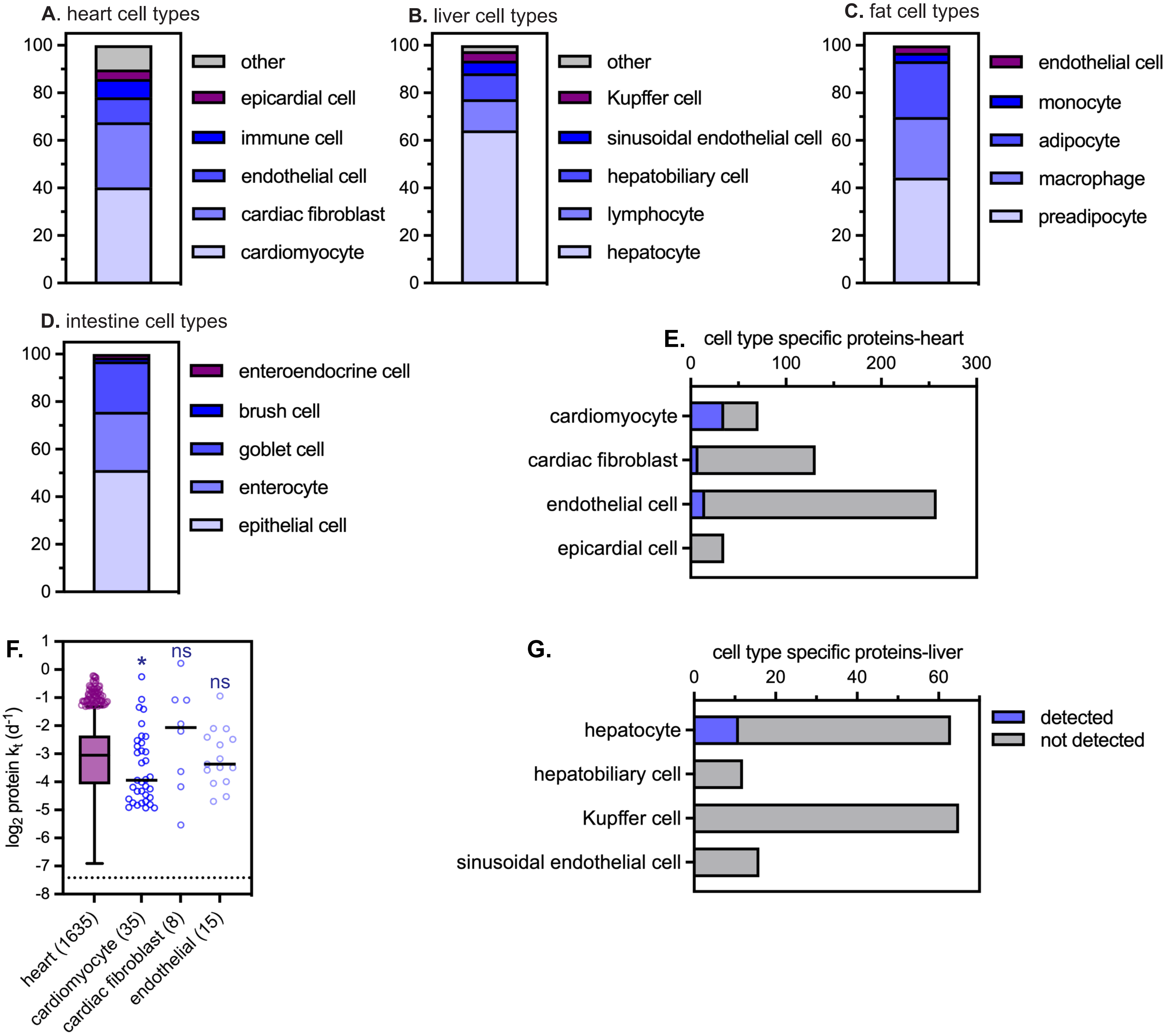

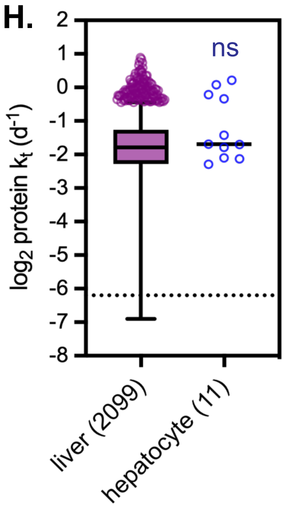

Major cell types of the heart (A), liver (B), white adipose tissue (C), and intestine (D), based on single cell sequencing-based analyses. Detection of cell-type-specific proteins in the heart (E) and liver (G) and comparison of turnover rate of cardiomyocyte, cardiac fibroblast, or endothelial-specific proteins to the bulk heart proteome (F); and of hepatocyte-specific proteins to the bulk liver proteome (H). * indicates $p < 0.05$ by Mann-Whitney test.

Appendix Figure S14

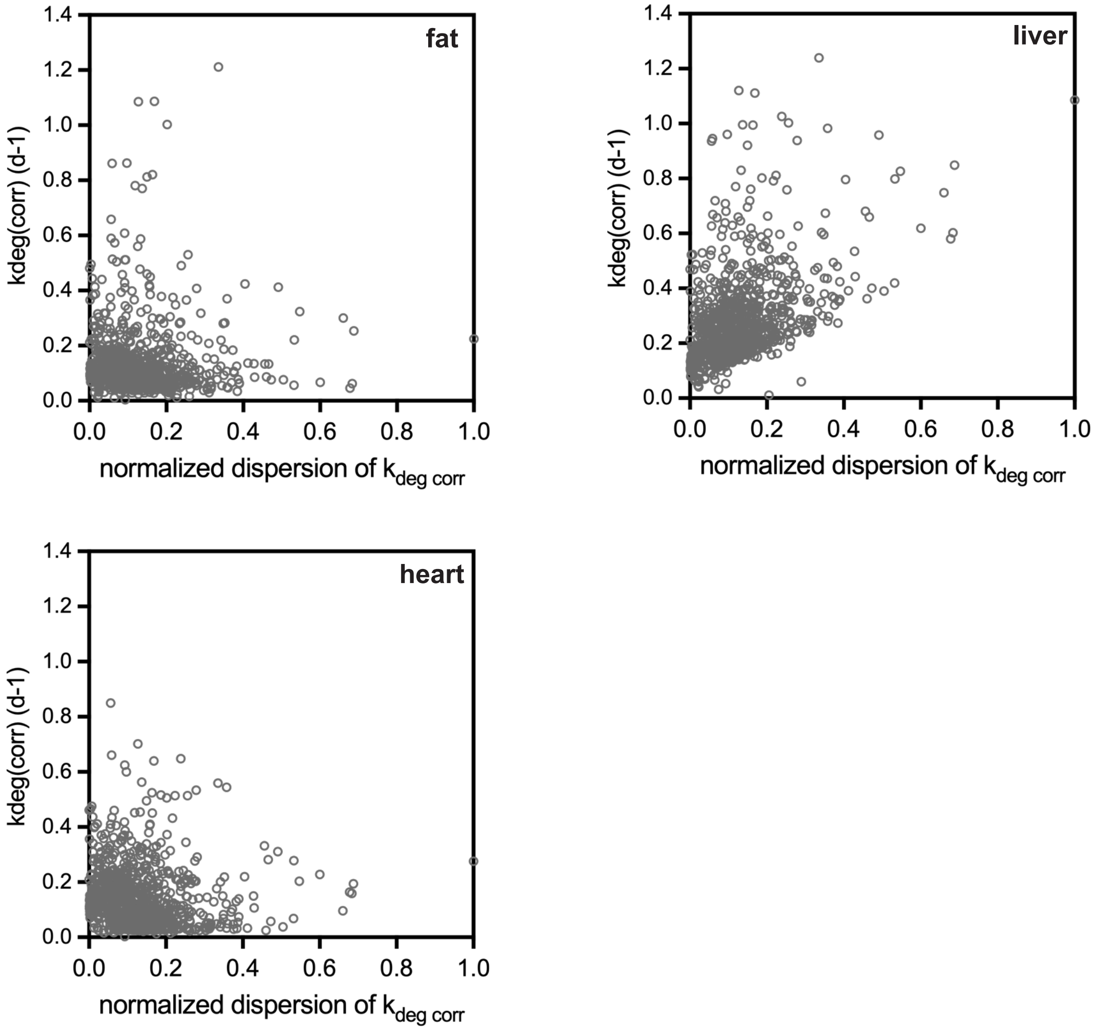

Normalized cross-tissue dispersion of k_{deg} is not correlated to the magnitude of k_{deg} in each tissue.

Appendix Fig. S15.

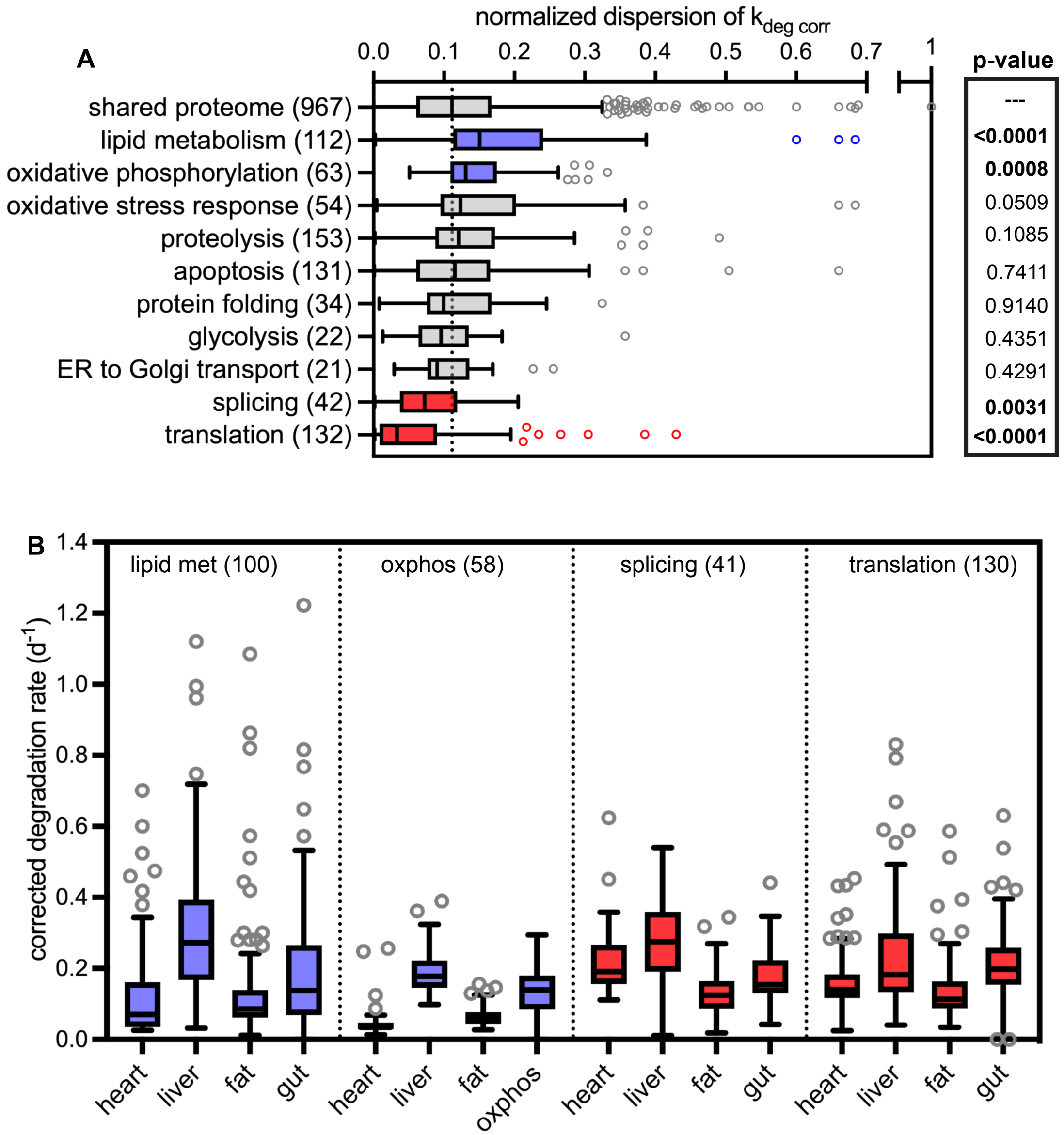

(A) Analysis of normalized cross-tissue dispersion of k_{deg} by KEGG pathway.
Blue indicates metabolic pathways with components that have significantly highervariability in their degradation rates across tissues vs. the proteome median; red indicates metabolic pathways with significantly lower variability across tissues vs. the proteome median. Dotted line indicates proteome median. Significance determined by Mann-Whitney test. (B) k_{deg} values of protein components of selected KEGG pathways across tissues, including proteins detected in the intestine for comparison. Blue indicates a subset has a significantly elevated median dispersion of k_{deg} values across tissues; red indicates a subset has a significantly decreased dispersion of k_{deg} values across tissues.

Appendix Figure S16.

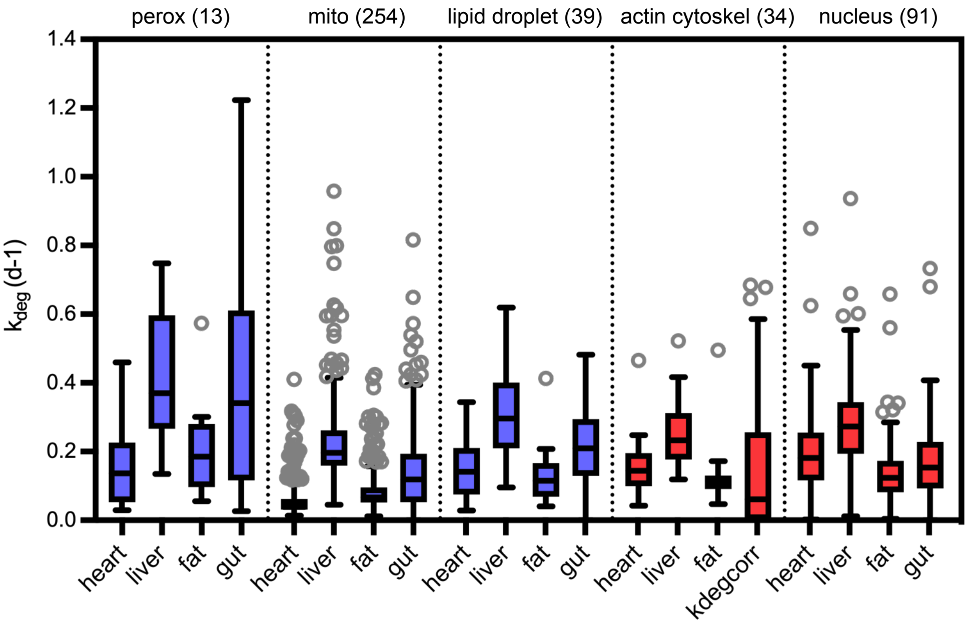

k_{deg} values of protein components of selected organelles across tissues, including proteins detected in the intestine for comparison. Blue indicates a subset has a significantly elevated median dispersion of k_{deg} values across tissues; red indicates a subset has a significantly decreased dispersion of k_{deg} values across tissues.

Appendix Figure S17.

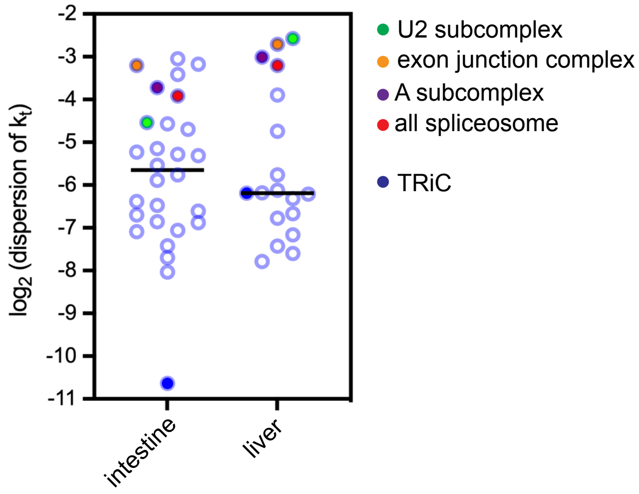

Analysis of intra-complex dispersion for multiprotein complexes
and for the U2, exon junction, and A sub-complexes of the spliceosome, which were detected with at least 5 subunits detected in the intestine and liver.
