# Supplementary material for: Turnover and replication analysis by isotope labeling (TRAIL) reveals the influence of tissue context on protein and organelle lifetimes
Source: Mol Syst Biol. 2023 Mar 17;19(4):e11393. doi: 10.15252/msb.202211393 (PMC10090950; doi:10.15252/msb.202211393)
Supplement: Supplementary file 11 [file MSB-19-e11393-s009.pdf]

molecular systems biology

Turnover and replication analysis by isotope labeling reveals control of protein lifetime in vivo

John Hasper, Kevin Welle, Jennifer Hryhorenko, Sina Ghaemmaghami, and Abigail Buchwalter
DOI: 10.15252/msb.202211393

Corresponding author(s): Abigail Buchwalter (abigail.buchwalter@ucsf.edu)

Review Timeline:

Submission Date:	12th Oct 22
Editorial Decision:	9th Nov 22
Revision Received:	9th Jan 23
Editorial Decision:	6th Feb 23
Revision Received:	13th Feb 23
Accepted:	16th Feb 23

Editor: Maria Polychronidou

Transaction Report:

(Note: With the exception of the correction of typographical or spelling errors that could be a source of ambiguity, letters and reports are not edited. Depending on transfer agreements, referee reports obtained elsewhere may or may not be included in this compilation. Referee reports are anonymous unless the Referee chooses to sign their reports.)

Thank you again for submitting your work to Molecular Systems Biology. We have now heard back from the three reviewers who agreed to evaluate your study. As you will see below, the reviewers raise substantial concerns, which preclude the publication of the manuscript in its current form.

While reviewer #3 is more supportive, reviewers #1 and #2 point out that the study remains somewhat preliminary. They mention that several conclusions need to be better supported and that as it stands the level of biological insight remains limited. Nevertheless, the reviewers acknowledge that the approach and findings are a potentially relevant contribution to the field and they provide constructive suggestions on how to revise and extend the study in order to address their concerns and make it suitable for publication in MSB. Taken together and considering that the reviewers acknowledge the overall relevance, we would like to offer you a chance to address the issues raised in a major revision. Without repeating all the points listed below, some of the more fundamental issues are the following:

- Reviewer #1 mentions that the lack of cell type resolution in the presented approach should be addressed, as it represents a limitation.

- Reviewers #1 and #2 mention that follow up analyses are required in order to enhance the level of biological insight provided by the study. Both of them provide suggestions in this regard, and this is an important point to address experimentally during revision.

Reviewer #1:

The manuscript by Hasper et al. investigated protein turnover in mouse organs using stable isotope labelling coupled to mass spectrometry. The authors devised a strategy by which they can estimate in the same experiment the rate of cell division within a tissue and half-life of proteins. Labelling was achieved by feed mice a N15 diet over a 32-day period and protein half-lives were estimated using Tandem Mass Tags (TMT)-based quantitative mass spectrometry. The use of N15 labelling to estimate protein half-lives is not novel and it was used previously by the same authors and other groups. The authors show that their new data based on TMT yields estimates of protein half-lives that are consistent with previous studies. The major novelty of the study is the parallel estimation of cell division rates, which is achieved by quantifying the incorporation of N15 in di-nucleotides obtained from DNA extracted from the organs investigated. Using these estimates, the authors were able to deconvolute the contribution of cell division to the observed protein half-lives and correct them across organs to obtain protein degradation rates (kdeg), which are independent of differences in cell division between organs. Using these new values, the authors demonstrate that differences in protein degradation rates remain across organs even after correcting for differences in cell division rates. They also pinpoint specific organelles and protein complexes that display higher variation of protein degradation rates across organs. They also show that even within the same organelle, i.e., mitochondrion, different proteins exhibit distinct patterns of protein degradation across organs. Although the manuscript presents an interesting approach to address a so far unexplored question, i.e., the contribution of cell division to protein turnover in mammalian organs, the lack of cell type resolution and the limited novel biological insights derived from the data preclude the publication of this work in its present form.

Major criticisms

• A major limitation of the presented approach is that cell division rates are calculated for bulk tissue, however different cell types within the same organ can have drastic differences in cell division rates. The intestine is the most prominent example with stem and progenitor cells having high division rates, while secretory cells and enterocytes are largely post-mitotic under homeostatic conditions. Therefore, correcting all the proteins, irrespectively from which cell types they are from, for the same value could lead to erroneously corrected protein degradation rates. A potential solution would be to estimate cell division rates for different cell populations by isolating them using FACS and correct cell type specific proteins (determined for example from available single cell RNAseq data) for that value.
• The level of biological insights derived from the data is limited. Although the reviewer appreciates the importance of estimating the contribution of cell division to protein half-lives (even with the caveats stated above), the novel findings presented in Figures 6 and 7 remain somehow limited and largely unexplained from a functional and mechanistic point of view. The potential explanations provided for why turnover of protein complexes and organelle differ between tissue remain highly speculative without further experiments.

Minor criticisms
• In Figure 7C, the spliceosome appears to be an outlier among protein complexes by showing higher dispersion. However, spliceosomes are composed of different individual complexes that should be investigated individually (see for example Hegele et al. Mol Cell 2012). Differences in turnover of these complexes could be explain why spliceosomes, considered as whole, display higher dispersion.

• The authors should investigate in their dataset the behaviour of previously determined Non-Exponentially Degraded (NED) members of protein complexes (McShane et al., Cell 2016 and Zavodszky et al., Science 2022), and assess their protein degradation behaviour relative to other complex members across organs.
• Why is intestine missing in panel 5E and F?
• Could the author speculate why protein complexes do not show lower dispersion in the intestine compared to a random set of proteins? Might this relate to differences among cell types of the intestine (see also major criticism above)?
• The "Analysis of Proteomic Data" paragraph mentions analysis of a set of progeroid animals in the TMT set. However, data from progeroid mice are not presented in the manuscript except for Supplementary Figure 2. Can the authors clarify?
• Was there any cut-off applied on the signal-to-noise ratios of the reporter ions used for analysis? If so, how was this cut-off determined?

Reviewer #2:

In this manuscript, the sought to explore how protein lifespan is regulated in different tissues depending on changes in proliferation rate and physicochemical features. To this end, the authors developed a method to analyse both protein lifespan and cell lifetimes, which they called "turnover and replication analysis by 15N isotope labeling (TRAIL)". Their data indicates that that sequence-based selectivity in protein turnover is negatively correlated with proliferation rate of tissues. Importantly, their analysis shows that protein and organelle turnover still vary non-randomly after correcting for cell proliferation rates. Moreover, they further expand their analysis to the organelle and multiprotein complex level. Their data suggest that some organelles such as mitochondria have variable lifetime across tissues, while multiprotein complexes have similar lifetimes across tissues.

In summary, the manuscript establishes the TRAIL method that can have important implications in future research to study how aging, disease and the environment affect protein, organelle and cell homeostasis. The authors also provide rich datasets on the links between protein lifespan and cell proliferation as well as organelle function. As such, I think the manuscript is of interest for a broad audience and can have important implications in different fields. On the other hand, one of the main concerns is that the authors made some strong statements based on their proteomics analysis that would need functional validation. Thus, further experiments would be really appreciated and necessary to support the conclusions. However, I understand that this is likely not the aim of the authors for this manuscript and not the main scope of the journal. Then, the authors should at least tone down the conclusions and make clear that further experiments are needed. The manuscript is very interesting, but there are some important points that I hope the authors can address:

- The authors should provide the principal component analysis (PCA) to show the abundance variation between samples. This is particularly important because in their experimental setup they used 2 female and 1 male mouse at each time point (except time zero, where they used 2 mice). Thus, it is important to show that the variation between different gender does not have an impact on their experimental design.

- In supplementary figure 10, the authors tested how accurately TRAIL can reports cell division with an experiment using isolated fibroblasts from the mouse ear. Although this experiment gives general confirmation about methodology, the proliferative rate of isolated fibroblasts is really low. Therefore, to better validate the methodology, the author should add cell lines with a higher proliferative rate such as immortalized MEFs, HEK or HELA cells.

- Figure 5C presents the proportion of proteome depending on the comparisons k div rates and kt rates. However, this representation excludes the proteins which have slow turnover rates that exceed the rate of cell division. The author should provide a graph representing all proteome including k div < k t rates.

- With the data presented in Figure 5, the authors concluded that long-lived proteins are non-selectively diluted by cell division in highly proliferative tissues but are slowly and selectively decreased in less proliferative tissues. However, this conclusion is too broad and the authors do not provide sufficient data for this conclusion.

- In the section entitled: "multiprotein complex subunits have coherent lifetimes within and across tissues", the authors conclude that assembly into stable multiprotein complex is not sufficient to dramatically extend protein lifetime. I think the authors cannot conclude this based on the data presented. The abundance of these subunits is not necessarily correlated directly with functional multiprotein complex formation.

Reviewer #3:

The paper performs extensive analysis of protein turnover across different mouse tissues by using isotopic labelling. A new technological development is the estimation of cell doubling time in tissues by leveraging the fact that 15N gets incorporated into DNA. This is an elegant strategy as it also allows to gain a better understanding of true protein turnover rate in tissues. The analysis of organelle and complex specific turnovers is well executed and well placed within the existing literature, uncovering new trends as well as corroborating previous findings. I found the manuscript easy to read and clearly structured and the

methods well described. I only have minor comments and would recommend publication once they are addressed.

Minor comments:
It would good to discuss the turnover of nuclear pore proteins in different tissues observed in this work as it has been a long-discussed topic in the field whether some npc do not turn over at all.
One way to estimate cell division time is to look at the turnover of the ca 5% slowest turnover proteins. It would be interesting to compare the results using this metric with TRAIL.

We would like to thank the reviewers and editorial staff of *Molecular Systems Biology* for taking the time to thoughtfully review our manuscript. Our revised manuscript includes new analyses and edits to increase the clarity and impact of our study. Specific responses to reviewer critiques follow below, with reviewer comments in black and our responses in blue. Corresponding edits in the manuscript text are highlighted in yellow in the revised manuscript document.

Reviewer 1 – major critiques

1. A major limitation of the presented approach is that cell division rates are calculated for bulk tissue, however different cell types within the same organ can have drastic differences in cell division rates. The intestine is the most prominent example with stem and progenitor cells having high division rates, while secretory cells and enterocytes are largely post-mitotic.. therefore, correcting all the proteins irrespectively of the cell types from which they are from.. could lead to erroneously corrected protein degradation rates.

We thank the reviewer for raising this important point; we have performed some new analyses to address this question. First, a clarification: measurements of cell turnover are reflective both of proliferation (e.g. of stem/progenitor cells in the intestine) and of cell death (e.g. shedding / apoptosis of terminally differentiated enterocytes in the intestine). At homeostasis, when organ size is unchanged, cell proliferation and cell death occur at equal rates and contribute equally to overall observed cell turnover. Importantly for our study, both cell division and cell death contribute to protein turnover.

Bulk cell turnover rates are a weighted average of turnover of all the cell types in a tissue, such that both relative abundance and turnover rate influence the overall turnover detected. We referred to single cell atlases such as *Tabula Muris* to estimate the 4-5 most abundant cell types in each tissue analyzed, and these estimates appear in a new Appendix Figure (Appendix Fig. S11). In the cases where proteins or transcripts unique to these cell types have been clearly defined, we were able to ask whether cell-type-specific proteins are detected in our datasets. Overall, we found that we could detect a small number (<40) of proteins that are unique to the most abundant cell type in a tissue, and could not detect proteins that are uniquely expressed by rare cell types. This indicates that overall our datasets report the 1000-2000 most abundant proteins per tissue, which are broadly expressed and are not cell-type-restricted. It is thus reasonable to evaluate how the tissue average of cell turnover relates to the tissue average of protein turnover, as we do in this study.

We could detect 35 cardiomyocyte-specific proteins in our heart proteome dataset, and we observed that this group of proteins do turn over significantly more slowly than the bulk heart proteome (median k_t for heart proteome, 0.12 d^{-1}; median k_t for cardiomyocyte proteome, 0.06 d^{-1}; $p = 0.0360$, Mann-Whitney test). This data is now included in Appendix Figure S11. As cardiomyocytes are thought to be very long-lived cells, it could be that proteins expressed in this cell type are also uniquely long-lived. However, it is important to note that the heart cell turnover rate (0.006 d^{-1}) is 1-2 orders of magnitude lower than heart protein turnover rates, which returns us to a major finding of our study: that cell turnover and protein turnover occur at comparable rates in proliferative tissues, while protein turnover occurs more rapidly than cell turnover in non-proliferative tissues.

These points are now discussed in the Results section of the revised text (lines 267-286).

2. A potential solution would be to estimate cell division rates for different cell populations by isolating them using FACS and correct cell type specific proteins for that value.

We agree with the reviewer that achieving cell-type-resolved measurements of cell turnover and protein turnover would be impactful and exciting. However, the specific experiment the reviewer proposes would have limited utility, as we have established that very few cell-type-specific proteins are quantified within our datasets (see Appendix Figure S11). To achieve a cell-type-resolved measurement of cell and protein turnover would require FACS isolation and mass spectrometric analysis of cells from tissues over 6 timepoints and within 3 replicates per timepoint – a very significant undertaking in terms of hours at the bench, yield of cells needed for each sample, and costs of instrument and analysis time.

To our knowledge, no one has yet performed a cell-type-resolved metabolic labeling timecourse by mass spectrometry of any primary cell type isolated from tissue. Cell-type-resolved measurements of proteome composition (that is, a single endpoint measurement) are in nascent stages by leaders in the proteomics field but have not yet been broadly adopted. The major bottleneck here is the amount of input material required; as proteomic technologies become more sensitive this will become more attainable. We are actively working on achieving cell type resolution with TRAIL, but meeting this long-term goal still requires major improvements in sample yield and detection sensitivity, putting this goal beyond the scope of this manuscript.

3. The level of biological insights derived from the data is limited, although the reviewer appreciates the importance of estimating the contributions of cell division to protein half-lives.

The major point of our study is to evaluate the relative contributions of “nature” (meaning sequence features) and “nurture” (meaning tissue environment, including cell division) on the turnover of broadly expressed proteins. The question of cell type resolution is irrelevant to this main point. Our work significantly advances the conversation in the protein homeostasis field, which has focused on the forces that influence protein turnover rate in model organisms (e.g. yeast) or in cultured cells; we test how the predictions of these studies apply *in vivo*. We show that intrinsic sequence features (hydrophobicity, disorder) cannot alone control protein turnover *in vivo*, but that tissue environment and cell division rate also shape protein turnover. The implication of these observations is that proteins, multiprotein complexes, and organelles “age” differently in each tissue.

<u>Reviewer 1 – minor critiques</u>

1. In Figure 7C, the spliceosome appears to be an outlier among protein complexes by showing higher dispersion. However, spliceosomes are composed of different individual complexes that should be investigated individually.

Thank you for this suggestion and for this very useful reference! Using the annotations from Hegele et al. *Mol Cell* 2012, we were able to parse protein turnover by spliceosome subcomplex. We focused this analysis on the liver and intestine, where we had especially good coverage of the spliceosome and detected 3 subcomplexes with at least 5 components: the exon junction complex, the U2 complex, and the A complex. Interestingly, even within these smaller subcomplexes we saw high intra-subunit dispersion in turnover rates. This data now appears in Appendix Figure S15. We speculate that this unusually high variability reflects the transience of interactions between many spliceosomal subunits – as Hegele et al. point out, > 30 proteins are exchanged during each of several catalytic steps in the splicing cycle. We discuss this data in the revised Results section, lines 424-431.

2. The authors should investigate in their dataset the behavior of non-exponentially degraded (NED) members of protein complexes.

NED behavior of protein complex subunits is a very intriguing question. NED proteins exhibit two distinct phases of decay: they are less stable shortly post-synthesis, then become relatively more stable as they mature. McShane et al. proposed that NED behavior arises for subunits of multiprotein complexes that are degraded more readily as nascent proteins but are protected from degradation once they are assembled into a complex. We do not expect to be able to resolve the first phase of rapid turnover kinetics for nascent NED proteins, as our shortest timepoint analyzed was 2 days of labeling. It is likely that we can only quantify the second, slower phase of turnover of NED proteins. For this reason, we cite McShane et al. briefly in the manuscript (see line 404 and 411) but do not explicitly raise the discussion of NED proteins.

Nevertheless, in response to the reviewer's query, we asked whether NED proteins have turnover rates that are unique in any way compared to other proteins subunits of multiprotein complexes. We performed this analysis on the liver proteome, where we had especially high coverage. Overall, we found that NED proteins turn over at similar rates to other complex partners; this is consistent with McShane et al.'s observation that while the first phase of NED protein turnover was often faster than other multiprotein complex partners, the second phase of NED protein turnover was similar to other complex partners.

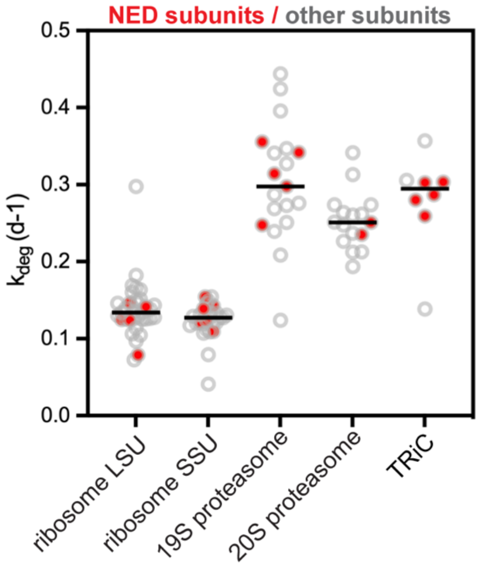

When we evaluated the cross-tissue dispersion of turnover for NED proteins, we noticed that these proteins do have a low variability in turnover rate across tissues. Since many of the NED proteins are multiprotein complex subunits, we infer that this observation is a correlate of our finding that multiprotein complexes generally have less variable turnover across tissues than other segments of the proteome.

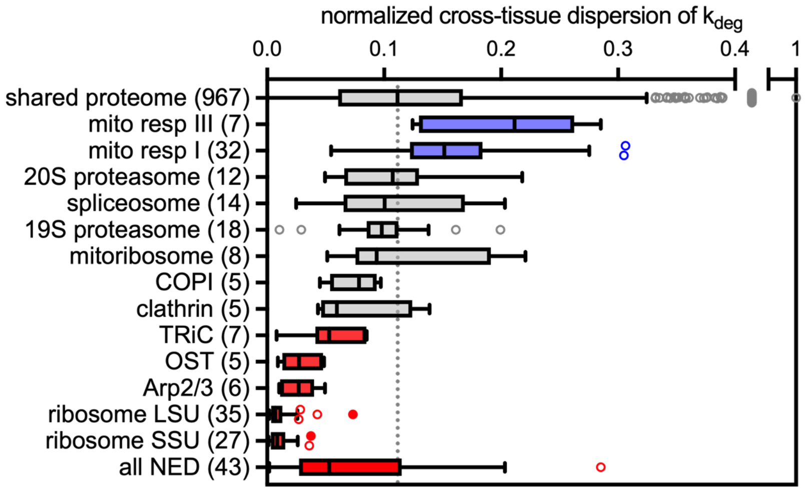

3. Why is intestine missing in panel 5E and 5F?

We find that cell turnover and protein turnover occur at comparable rates in the proliferative intestine, while protein turnover occurs an order of magnitude more rapidly than cell turnover in non-proliferative tissues. As a consequence of this, correcting protein turnover rates in the intestine for the rate of cell turnover causes predicted protein degradation rates of many proteins to approach zero. This effect introduces major error into dispersion analyses. We have adjusted Figure 5 and the accompanying text to make more clear that the relationship between protein turnover and cell turnover is fundamentally different in proliferative tissues than in non-proliferative tissues.

4. Why do protein complexes not show lower dispersion in the intestine compared to a random set of proteins? Might this relate to differences among cell types of the intestine?

Thank you for raising this point. We realized that there was a minor error in our analysis of these data which once corrected revealed that multiprotein complexes in the intestine *actually do* show low intra-complex dispersion. In our original analysis, we had erroneously used cell cycle-corrected *kdeg* values to compute intra-complex dispersion, and not uncorrected *kt* values. This introduced error into the intestine analysis because the cell cycle-corrected turnover of some proteins in the intestine approaches zero after correction. The corrected analysis is now shown in Fig. 7C.

The proteomic datasets we acquired have coverage of broadly expressed and generally not of cell type-specific proteins (see response to Major Critique 1 above); further, the multiprotein complexes analyzed are essential to the function of every cell type (e.g. mitochondrial respiratory chain, ribosomes, etc).

5. The “Analysis of Proteomic Data” paragraph mentions analysis of progeroid animals…. Can the authors clarify?

We have also applied TRAIL to a mouse model of Hutchinson-Gilford progeria syndrome (HGPS) and had at an earlier point in time planned to include that data in this manuscript. While TRAIL has revealed some interesting features of progeria that we are planning to report soon, this line of experiments took a very different direction than the focus of this manuscript and we felt the

best course of action was to present that work in a separate manuscript. We thank the reviewer for catching this confusing discrepancy in the text and the supplement; we have corrected these in the revised manuscript.

6. Was there any signal to noise cutoff for reporter ions in TMT?

Yes; we used Proteome Discoverer for TMT data analysis, and set the average reporter ion signal-to-noise threshold to 5, such that only spectra with an average reporter S/N value above this threshold are quantified. The average reporter ion S/N value is an average of all reporter peaks in the spectrum. We have added this information to the Methods under the LC-MS/MS analysis section.

<u>Reviewer 2 major critiques</u>

1. the authors should provide principal component analysis to show the abundance variation between samples. This is particularly important because in their experimental setup they used 2 female and 1 male mouse at each time point… thus it is important to show that the variation between different sexes does not have an impact on their experimental design.

While the reviewer requests PCA to show abundance variation between samples, we do not actually have quantitative protein abundance data for each sample from each timecourse, because timecourse samples are a mixture of 15N and 14N labeled proteins which complicates abundance quantification. We did use the unlabeled (day 0) samples to evaluate relative protein abundance and to compare the relationship between protein abundance and protein turnover rate. This comparison, which shows little to no correlation, is shown in Appendix Figure S4. While we are not able to say whether protein abundances vary significantly between sexes, this analysis suggests that a change in protein abundance would not correlate with a change in protein turnover rate.

As shown in Appendix Figure S1, we used similar overall numbers of male and female mice for labeling timepoints: day 2 (2 female, 1 male), day 4 (2 male, 1 female); day 8 (2 female, 1 male), day 16 (2 male, 1 female), and day 32 (2 female, 1 male); this adds up to 8 female and 7 male mice. Curve fitting and determination of kinetic parameters was performed on pooled data. This experimental setup and analysis approach precludes sex-resolved PCA or other analyses. We recognize the importance of considering sex as a biological variable and intentionally chose this study design to balance priorities for these expensive and labor-intensive experiments. On the one hand, this approach allowed us to evaluate average protein turnover behavior across both sexes rather than biasing our analysis by restricting to a single sex. On the other hand, we realized that providing a sufficiently powered analysis of sex differences in protein turnover would require doubling the size of the animal cohort, which was not feasible. Whether differences in protein turnover rate contribute to differences between sexes is an intriguing question that is outside the scope of this current study.

2. In Appendix Figure S10, the authors tested how accurately TRAIL can report cell division with an experiment using isolated fibroblasts from the mouse ear. Although this experiment gives general confirmation about methodology, the proliferative rate of isolated fibroblasts is low… to better validate the methodology, the authors should add cell lines with a higher proliferative rate.

While we agree that it would be interesting to directly compare TRAIL and cell numbers to additional cell types, TRAIL cannot be applied to cells in culture as the reviewer suggests. This is because unlike other types of metabolic labeling that can be applied in cell culture (e.g. SILAC,

which provides labeled amino acids for proteome labeling), it is not currently possible to make defined cell culture media containing ^{15}N at 100% isotope abundance in order to label both DNA and proteins. That aside, the major goal of our study was to develop techniques for quantifying protein and cell turnover within tissues, which TRAIL achieves. The goal of the experiment shown in Appendix Figure S10 was to benchmark the technique in a primary cell type. The doubling time of the isolated fibroblasts that we analyzed was between 1.3-1.5 days. We were able to track these cells over several population doublings and make a direct comparison between TRAIL and cell numbers, validating our methodology.

3. Figure 5C…excludes the proteins which have slow turnover rates that exceed the rate of cell division. The authors should provide a graph representing all proteome including *kdiv* < *kt* rates.

We have made some changes to Figure 5 and to the accompanying text to increase clarity. In Panels 5A and 5B, we now show a direct comparison between cell turnover and proteome turnover. We compare cell kdiv to the distribution of protein kt in each tissue, and also to the longest-lived decile of proteins in each tissue. This comparison clearly shows that the longest-lived proteins in the intestine turn over at a rate equivalent to or even slower than the rate of cell division in the intestine; in contrast, the longest-lived proteins in the liver, heart, and fat still turn over faster than the rate of cell division.

Panel 5C actually quantifies all proteins with a turnover rate *kt* equivalent to or slower than cell *kdiv.* We have adjusted the legend and labels to make this more clear; about 15% of the intestine proteome turns over equivalently to or even slower than the rate of cell division.

4. With the data presented in Figure 5, the authors concluded that long-lived proteins are non-selectively diluted by cell division in highly proliferative tissues but are slowly and selectively decreased in less proliferative tissues. However, this conclusion is too broad and the authors do not provide sufficient data for this conclusion.

We have followed the reviewer's advice to tone down our interpretations of this data in the text in the Abstract (lines 22-26), in the Results (lines 290-296) and in the Discussion (lines 464-467). What is clear from our data is that the relative magnitude of protein turnover and cell turnover are comparable in the intestine, while protein turnover occurs an order of magnitude more rapidly than cell turnover in the liver, heart, and fat. We have made this comparison more explicit by directly comparing *kdiv* and *kt* in figure panels 5A and 5B. Separately, we could find various sequence features that correlate significantly with protein turnover rate in the slowly proliferative liver, heart, and fat, yet none of these features correlate with protein turnover rates in the highly proliferative intestine (Figure 4A). Based on this observation, we conclude that protein turnover is less sequence-selective in the intestine. While it is tempting to speculate that sequence specificity of turnover is lower because non-selective dilution by cell division is higher, we agree with the reviewer's critique that we have not experimentally supported this claim, and we refrain from making this claim in the revised manuscript.

5. The authors conclude that assembly into stable multiprotein complex is not sufficient to dramatically extend protein lifetime. I think the authors cannot conclude this based on the data presented. The abundance of these subunits is not necessarily correlated directly with functional multiprotein complex formation.

Please see also our response to Reviewer 1's question about non-exponentially decaying (NED) proteins above, which may be relevant to this question.

We agree that our data cannot determine whether assembly into a multiprotein complex stabilizes a protein *relative to its unassembled state.* Previous work referenced by Reviewer 1 and also within our manuscript (McShane et al., Cell 2016) indicates that some multiprotein complex subunits are more rapidly degraded as nascent proteins, but are stabilized by multiprotein complex assembly. However, our data do indicate that multiprotein complex subunits are not generally more long-lived *compared to the rest of the proteome*, which includes many proteins that do not participate in multiprotein complexes.

We have adjusted the language in the Results section entitled “Multiprotein complex subunits have coherent lifetime within and across tissues” to reflect this nuanced point (lines 408-410).

<u>Reviewer 3</u>

1. It would be good to discuss the turnover of nuclear pore complex proteins in different tissues observed in this work as it has been a long-discussed topic in the field whether some NPC do not turn over at all.

Thank you for this suggestion. We had sufficient coverage (>5 subunits) of the NPC to perform intra-complex dispersion analysis in the liver and intestine, and that data is included in Panel 7C. We had low coverage of the NPC in the fat and heart datasets.

Core components of the NPC have been previously shown to be long-lived in the brain, but not in the liver (Toyama et al., Cell 2013) and in cultured cells (Toyama et al., J Cell Biol 2019); however other data in cultured cells challenged this point (Mathieson et al., Nat Comms 2018). We had coverage of some subunits of the Nup93 subcomplex of the NPC, a core component of the NPC barrel that has been shown by SILAM (Toyama et al., 2013) and by microscopy-based pulse chase experiments (Toyama et al., 2019) to be especially long-lived.

Our data indicate that the Nup93 complex turns over at a rate similar to cell division in the intestine, fat, and heart, but turns over significantly faster than kdiv in the liver. The relatively short NPC lifetime in the liver has been reported before (Toyama et al Cell 2013) but it was assumed that this reflected the faster cell turnover of this tissue. However, our data show that this is not the case. We now include these data in Figure 5E and discuss these findings in the results section entitled “Protein degradation rates vary across tissues after cell cycle correction” (lines 314-323).

2. One way to estimate cell division time is to look at the turnover of the ~5% slowest turnover proteins. It would be interesting to compare the results using this metric with TRAIL.

Thank you for this suggestion. In response to comments from each of the reviewers, we have restructured Figure 5 and the accompanying text to increase clarity, including incorporating this suggestion. We now directly compare cell division kinetics determined by TRAIL DNA labeling to the turnover of the bottom 10% of the proteome. (We chose the bottom 10% rather than 5% because we see 10-15% of intestinal proteins turning over at a similar rate to cell division.)

This clearly illustrates the contrast between proliferative tissues – where cell turnover and protein turnover occur at similar rates of flux – versus slowly proliferative tissues, where cell turnover occurs orders of magnitude more slowly than almost all protein turnover. Notably, we highlight a few extremely long-lived proteins such as Histone H3.1 and core nuclear pore complex components that approach the rate of cell division.

1st Revision - Editorial Decision 6th Feb 2023

Thank you for sending us your revised manuscript. We have now heard back from the two reviewers who were asked to evaluate your revised study. As you will see below, the reviewers are satisfied with the performed revisions and support publication. Reviewer #1 only lists a remaining issue, which we would ask you to fix in a revision. We would also ask you to address some remaining editorial issues listed below.

Reviewer #1:

I am overall satisfied with the revised manuscript and the argumentations provided by the authors regarding the points I raised. One point that remains to be clarified is why the analysis of cell-type specific proteins in Appendix Figure S11 is presented only for liver and heart? Does this mean that cell-type specific proteins were not detected for intestine and fat? If so, please clarify. Additionally, it would be beneficial to flag cell-type specifc proteins in the Dataset EV1 and EV5, for transparency.

Reviewer #2:

The authors addressed all my concerns successfully, and the manuscript has been greatly improved. I support its publication

All editorial and formatting issues were resolved by the authors.

2nd Revision - Editorial Decision 16th Feb 2023

Thank you again for sending us your revised manuscript. We are now satisfied with the modifications made and I am pleased to inform you that your paper has been accepted for publication.

EMBO Press Author Checklist

Corresponding Author Name: Abigail Buchwalter	
Journal Submitted to: Molecular Systems Biology	
Manuscript Number: MSB-2022-11393R	

USEFUL LINKS FOR COMPLETING THIS FORM
The EMBO Journal - Author Guidelines
EMBO Reports - Author Guidelines
Molecular Systems Biology - Author Guidelines
EMBO Molecular Medicine - Author Guidelines

Reporting Checklist for Life Science Articles (updated January
This checklist is adapted from Materials Design Analysis Reporting (MDAR) Checklist for Authors. MDAR establishes a minimum set of requirements in transparent reporting in the life sciences (see Statement of Task: 10.31222/osf.io/9sm4x). Please follow the journal's guidelines in preparing your
Please note that a copy of this checklist will be published alongside your article.

Abridged guidelines for figures
1. Data
The data shown in figures should satisfy the following conditions:
- the data were obtained and processed according to the field's best practice and are presented to reflect the results of the experiments in an accurate and unbiased manner.
- ideally, figure panels should include only measurements that are directly comparable to each other and obtained with the same assay.
- plots include clearly labeled error bars for independent experiments and sample sizes. Unless justified, error bars should not be shown for technical
- if n<5, the individual data points from each experiment should be plotted. Any statistical test employed should be justified.
- Source Data should be included to report the data underlying figures according to the guidelines set out in the authorship guidelines on Data

2. Captions
Each figure caption should contain the following information, for each panel where they are relevant:
- a specification of the experimental system investigated (eg cell line, species name).
- the assay(s) and method(s) used to carry out the reported observations and measurements.
- an explicit mention of the biological and chemical entity(ies) that are being measured.
- an explicit mention of the biological and chemical entity(ies) that are altered/varied/perturbed in a controlled manner.
- the exact sample size (n) for each experimental group/condition, given as a number, not a range;
- a description of the sample collection allowing the reader to understand whether the samples represent technical or biological replicates (including how many animals, litters, cultures, etc.).
- a statement of how many times the experiment shown was independently replicated in the laboratory.
- definitions of statistical methods and measures:
  - common tests, such as t-test (please specify whether paired vs. unpaired), simple χ2 tests, Wilcoxon and Mann-Whitney tests, can be unambiguously identified by name only, but more complex techniques should be described in the methods section;
  - are tests one-sided or two-sided?
  - are there adjustments for multiple comparisons?
  - exact statistical test results, e.g., P values = x but not P values < x;
  - definition of 'center values' as median or average;
  - definition of error bars as s.d. or s.e.m.

Please complete ALL of the questions below.
Select "Not Applicable" only when the requested information is not relevant for your study.

Materials

Newly Created Materials	Information included in the manuscript?	In which section is the information available? (Reagents and Tools Table, Materials and Methods, Figures, Data Availability Section)
New materials and reagents need to be available; do any restrictions apply?	Not Applicable	

Antibodies	Information included in the manuscript?	In which section is the information available? (Reagents and Tools Table, Materials and Methods, Figures, Data Availability Section)
For **antibodies** provide the following information:<br>- Commercial antibodies: RRID (if possible) or supplier name, catalogue number and or/clone number<br>- Non-commercial: RRID or citation	Not Applicable	

DNA and RNA sequences	Information included in the manuscript?	In which section is the information available? (Reagents and Tools Table, Materials and Methods, Figures, Data Availability Section)
**Short novel DNA or RNA including primers, probes**: provide the sequences.	Not Applicable	

Cell materials	Information included in the manuscript?	In which section is the information available? (Reagents and Tools Table, Materials and Methods, Figures, Data Availability Section)
**Cell lines:** Provide species information, strain. Provide accession number in repository **OR** supplier name, catalog number, clone number, and/**OR** RRID.	Not Applicable	
**Primary cultures:** Provide species, strain, sex of origin, genetic modification status.	Not Applicable	
Report if the cell lines were recently **authenticated** (e.g., by STR profiling) and tested for mycoplasma contamination.	Not Applicable	

Experimental animals	Information included in the manuscript?	In which section is the information available? (Reagents and Tools Table, Materials and Methods, Figures, Data Availability Section)
**Laboratory animals or Model organisms:** Provide species, strain, sex, age, genetic modification status. Provide accession number in repository **OR** supplier name, catalog number, clone number, **OR** RRID.	Yes	Reagents and Tools Table; Materials and Methods
**Animal observed in or captured from the field:** Provide species, sex, and age where possible**.**	Not Applicable	
Please detail **housing and husbandry conditions**.	Yes	Materials and Methods

Plants and microbes	Information included in the manuscript?	In which section is the information available? (Reagents and Tools Table, Materials and Methods, Figures, Data Availability Section)
**Plants:** provide species and strain, ecotype and cultivar where relevant, unique accession number if available, and source (including location for collected wild specimens).	Not Applicable	
**Microbes:** provide species and strain, unique accession number if available, and source.	Not Applicable	

Human research participants	Information included in the manuscript?	In which section is the information available? (Reagents and Tools Table, Materials and Methods, Figures, Data Availability Section)
If collected and within the bounds of privacy constraints report on age, sex and gender or ethnicity for all study participants.	Not Applicable	

Core facilities	Information included in the manuscript?	In which section is the information available? (Reagents and Tools Table, Materials and Methods, Figures, Data Availability Section)
If your work benefited from core facilities, was their service mentioned in the acknowledgments section?	Not Applicable	

Design

Study protocol	Information included in the manuscript?	In which section is the information available? (Reagents and Tools Table, Materials and Methods, Figures, Data Availability Section)
If study protocol has been **pre-registered, provide DOI in the manuscript**. For clinical trials, provide the trial registration number **OR** cite DOI.	Not Applicable	
Report the **clinical trial registration number** (at ClinicalTrials.gov or equivalent), where applicable.	Not Applicable	

Laboratory protocol	Information included in the manuscript?	In which section is the information available? (Reagents and Tools Table, Materials and Methods, Figures, Data Availability Section)
Provide DOI OR other citation details if **external detailed step-by-step protocols** are available.	Not Applicable	

Experimental study design and statistics	Information included in the manuscript?	In which section is the information available? (Reagents and Tools Table, Materials and Methods, Figures, Data Availability Section)
Include a statement about **sample size** estimate even if no statistical methods were used.	Yes	Materials and Methods
Were any steps taken to minimize the effects of subjective bias when allocating animals/samples to treatment (e.g. **randomization procedure**)? If yes, have they been described?	Yes	Materials and Methods
Include a statement about **blinding** even if no blinding was done.	Not Applicable	
Describe **inclusion/exclusion criteria** if samples or animals were excluded from the analysis. Were the criteria pre-established?<br><br>If sample or data points were omitted from analysis, report if this was due to attrition or intentional exclusion and provide justification.	Yes	Materials and Methods; Results
For every figure, are **statistical tests** justified as appropriate? Do the data meet the assumptions of the tests (e.g., normal distribution)? Describe any methods used to assess it. Is there an estimate of variation within each group of data? Is the variance similar between the groups that are being statistically compared?	Yes	Figure Legends

Sample definition and in-laboratory replication	Information included in the manuscript?	In which section is the information available? (Reagents and Tools Table, Materials and Methods, Figures, Data Availability Section)
In the figure legends: state number of times the experiment was **replicated** in laboratory.	Yes	Results, Materials and Methods
In the figure legends: define whether data describe **technical or biological replicates**.	Yes	Results, Materials and Methods

Ethics

Ethics	Information included in the manuscript?	In which section is the information available? (Reagents and Tools Table, Materials and Methods, Figures, Data Availability Section)
Studies involving **human participants**: State details of **authority granting ethics approval** (IRB or equivalent committee(s), provide reference number for approval.	Not Applicable	
Studies involving **human participants**: Include a statement confirming that **informed consent** was obtained from all subjects and that the experiments conformed to the principles set out in the WMA Declaration of Helsinki and the Department of Health and Human Services Belmont Report.	Not Applicable	
Studies involving **human participants:** For publication of **patient photos**, include a statement confirming that consent to publish was obtained.	Not Applicable	
Studies involving experimental **animals**: State details of **authority granting ethics approva**l (IRB or equivalent committee(s), provide reference number for approval. Include a statement of compliance with ethical regulations.	Yes	Materials and Methods
Studies involving **specimen and field samples:** State if relevant **permits** obtained, provide details of authority approving study; if none were required, explain why.	Not Applicable	

Dual Use Research of Concern (DURC)	Information included in the manuscript?	In which section is the information available? (Reagents and Tools Table, Materials and Methods, Figures, Data Availability Section)
Could your study fall under dual use research restrictions? Please check biosecurity documents and list of **select agents and toxins** (CDC): https://www.selectagents.gov/sat/list.htm	Not Applicable	
If you used a select agent, is the security level of the lab appropriate and reported in the manuscript?	Not Applicable	
If a study is subject to dual use research of concern regulations, is the name of the **authority granting approval and reference number** for the regulatory approval provided in the manuscript?	Not Applicable	

Reporting

The MDAR framework recommends adoption of discipline-specific guidelines, established and endorsed through community initiatives. Journals have their own policy about requiring specific guidelines and recommendations to complement MDAR.

Adherence to community standards	Information included in the manuscript?	In which section is the information available? (Reagents and Tools Table, Materials and Methods, Figures, Data Availability Section)
State if relevant guidelines or checklists (e.g., **ICMJE, MIBBI, ARRIVE, PRISMA**) have been followed or provided.	Not Applicable	
For **tumor marker prognostic studies,** we recommend that you follow the **REMARK** reporting guidelines (see link list at top right). See author guidelines, under 'Reporting Guidelines'. Please confirm you have followed these guidelines.	Not Applicable	
For **phase II and III randomized controlled trials**, please refer to the **CONSORT** flow diagram (see link list at top right) and submit the CONSORT checklist (see link list at top right) with your submission. See author guidelines, under 'Reporting Guidelines'. Please confirm you have submitted this list.	Not Applicable	

Data Availability

Data availability	Information included in the manuscript?	In which section is the information available? (Reagents and Tools Table, Materials and Methods, Figures, Data Availability Section)
Have **primary datasets** been deposited according to the journal's guidelines (see 'Data Deposition' section) and the respective accession numbers provided in the Data Availability Section?	Yes	Materials and Methods
Were **human clinical and genomic datasets** deposited in a public access-controlled repository in accordance to ethical obligations to the patients and to the applicable consent agreement?	Not Applicable	
Are **computational models** that are central and integral to a study available without restrictions in a machine-readable form? Were the relevant accession numbers or links provided?	Not Applicable	
If publicly available data were reused, provide the respective **data citations in the reference list**.	Yes	Figure Legends, Methods
